# Biparental incubation patterns in a high-Arctic breeding shorebird: how do pairs divide their duties?

**DOI:** 10.1093/beheco/art098

**Published:** 2013-10-29

**Authors:** Martin Bulla, Mihai Valcu, Anne L. Rutten, Bart Kempenaers

**Affiliations:** Department of Behavioural Ecology & Evolutionary Genetics, Max Planck Institute for Ornithology, Eberhard Gwinner Str. 7, 82319 Seewiesen, Germany

**Keywords:** Arctic, Calidris pusilla, continuous daylight, incubation pattern, incubation timing, negotiation, nest attendance, parental care division, semipalmated sandpiper, sexual conflict.

## Abstract

In biparental species, parents may be in conflict over how much they invest into their offspring. To understand this conflict, parental care needs to be accurately measured, something rarely done. Here, we quantitatively describe the outcome of parental conflict in terms of quality, amount, and timing of incubation throughout the 21-day incubation period in a population of semipalmated sandpipers (*Calidris pusilla*) breeding under continuous daylight in the high Arctic. Incubation quality, measured by egg temperature and incubation constancy, showed no marked difference between the sexes. The amount of incubation, measured as length of incubation bouts, was on average 51min longer per bout for females (11.5h) than for males (10.7h), at first glance suggesting that females invested more than males. However, this difference may have been offset by sex differences in the timing of incubation; females were more often off nest during the warmer period of the day, when foraging conditions were presumably better. Overall, the daily timing of incubation shifted over the incubation period (e.g., for female incubation from evening–night to night–morning) and over the season, but varied considerably among pairs. At one extreme, pairs shared the amount of incubation equally, but one parent always incubated during the colder part of the day; at the other extreme, pairs shifted the start of incubation bouts between days so that each parent experienced similar conditions across the incubation period. Our results highlight how the simultaneous consideration of different aspects of care across time allows sex-specific investment to be more accurately quantified.

## Lay summary

Parents may be in conflict over the care they provide to their offspring. To understand this conflict, an accurate description of who does what and when is necessary. We used an automated system to continuously monitor which parent incubated the eggs in an arctic breeding shorebird. Birds sat on the eggs around 11 h at a time, but females sat longer than males. In compensation, females were off-duty more when feeding was easier.

## INTRODUCTION

Parental care is beneficial to offspring, but it is also costly, both energetically and in terms of lost opportunities for other activities such as self-maintenance or mating. Whereas these costs are paid by each parent individually, the benefits are shared by both. This, Trivers (1972) argued, creates potential for conflict between the parents because it is advantageous to minimize one’s own investment while capitalizing on the benefits that arise from the investment of the partner.

Theoretical models predict the outcomes of this conflict by considering 2 strategies (reviewed by Lessells 2012). In the first, the amount of parental investment is fixed at a specific level or follows a behavioral rule that determines the level of care irrespective of previous care of the partner. In the second strategy, the level of care is a result of behavioral negotiation.

Most of these models are not tailored to a specific empirical system (van Dijk et al. 2012), so their assumptions and subsequent empirical tests often miss some of the complexity of parental care (e.g., by modeling or measuring the individual costs inaccurately). For example, most studies measure the total amount of care (reviewed by Harrison et al. 2009), but this is not the same as measuring costs of care because providing better quality of care or providing care during tougher conditions may lead to higher costs. Also, even if both pair members invest overall equally, there may be large differences between pairs in how they achieve this, resulting in variation in patterns of care (i.e., different outcomes of parental conflict).

Quantifying such between-individual variability in investment is essential to approximate the variation that selection can act on (Bolnick et al. 2011) or to identify different parental care strategies. Therefore, an important step in understanding parental conflict and its outcome is to describe the complexity of parental care (e.g., in terms of quality, amount, and timing), including its temporal and between-individual variation. However, such detailed descriptions are rare, although they would provide the basis for specific models and subsequent empirical tests of the processes behind the described patterns of parental care.

Parental care takes a variety of forms; in birds, incubation of eggs is crucial for successful reproduction (Deeming 2002a). Although previous studies have investigated sex differences in particular aspects of incubation (Kleindorfer et al. 1995; reviewed by Deeming 2002b; Auer et al. 2007), we are not aware of any study that examined incubation patterns in terms of incubation quality, amount, and timing simultaneously, quantitatively, and throughout the incubation period.

Here, we used a continuous recording system to quantitatively describe the incubation patterns in a population of semipalmated sandpipers (*Calidris pusilla*), a common, Arctic-breeding, socially monogamous shorebird. We quantified how parents divided their duties over the incubation period, considering both variation and central tendency. Specifically, we measured 4 aspects of incubation: incubation temperature and incubation constancy (both measures of quality), length of incubation bouts (amount), and distribution of incubation within a day, over the incubation period, and over the season (timing).

The biparental incubation system of semipalmated sandpipers is well suited for these investigations for 3 reasons. First, several factors that may confound the outcomes of parental conflict in other systems can be excluded here. 1) Variation in clutch size is limited: semipalmated sandpipers lay 4 (rarely 3) eggs (Hicklin and Gratto-Trevor 2010). 2) Spatial variation in environmental conditions in the high-Arctic breeding grounds is small compared with temperate habitats; our study site consists of a homogeneous tundra environment. 3) In our high-Arctic study site, the nonincubating parent seems to provide no other form of care because it leaves for feeding grounds up to 2–3 km away from the nest (Ashkenazie and Safriel 1979a; Jehl 2006; our own observations). Second, the extreme rates of energy expenditure in the high Arctic (Piersma et al. 2003) should elevate the conflict over parental care. Third, biparental incubation is a type of parental care that involves mutually exclusive behavior (Kosztolanyi et al. 2009) and therefore, unlike other forms of care such as offspring provisioning, parents cannot change their contribution independently of each other.

## METHODS

### Study area and species

We studied a population of semipalmated sandpipers near Barrow, Alaska (71°32′N, 156°65′W), between 1 June and 16 July 2011; Ashkenazie and Safriel (1979a) have described the area in detail. In brief, the site consists of polygonal soils with a high-Arctic tundra vegetation (sedges, mosses, and lichens). Ambient temperatures are generally low, below 5 °C (Supplementary Figure S1a). However, surface tundra temperatures can reach up to 28 °C (Supplementary Figure S1b). Barrow has continuous daylight: the sun never sets between mid-May and the end of July. Nevertheless, environmental conditions show consistent and substantial diel fluctuations: tundra temperatures are ~85% and light intensity ~90% lower at night than during the day (Supplementary Figure S1b and c). In contrast, diel fluctuations in wind speed are less pronounced (Supplementary Figure S1d). Diel fluctuations may also exist in predatory pressure because the Arctic fox (*Alopex lagopus*), the main mammalian predator of shorebird eggs (e.g., Liebezeit and Zack 2008), is active mainly during night hours (Eberhardt et al. 1982). However, this effect is absent or strongly reduced in our study site because of an intense fox removal program in the Barrow area (foxes are shot and trapped). As a result, there was a tendency for increased nest predation (probably by avian predators such as skuas, *Stercorarius* sp.) during the day (Supplementary Figure S2).

Semipalmated sandpipers are small shorebirds and are monomorphic in plumage, but with females on average slightly larger than males (Supplementary Figure S3). The birds arrive at the Barrow breeding ground between the end of May and early June; males immediately establish territories; pairs form within 3–6 days, and egg laying starts shortly after (Ashkenazie and Safriel 1979a). A complete clutch has 4, rarely 3, eggs and a 4-egg clutch is typically laid in 5 days (Sandercock 1998). The species is socially monogamous, and extrapair paternity is rare (our unpublished data). Both sexes develop 2 lateral brood patches, and both parents incubate. Incubation lasts 19–22 days. Chicks are precocial, and females tend to desert the family 2–8 days after hatching (Ashkenazie and Safriel 1979a; Hicklin and Gratto-Trevor 2010).

### Sampling of individuals

Nonincubating birds were captured with mist nets (*N* = 22) and incubating birds with spring traps (*N* = 125). Spring traps were triggered from a distance by fishing line, and the captured bird was released from the trap within approximately 20 s. No eggs were damaged by this catching method. Adults were marked with an aluminum US Geological Survey band, a unique combination of 4 color bands, and a green flag with embedded glass passive–integrated tag (Biomark: 9.0mm × 2.1mm, 0.087g, 134.2kHz, ISO FDXB, http://www.biomark.com/; Supplementary Picture S1). For the purpose of another project and following Warnock and Warnock (1993), 40 individuals were equipped with a radio transmitter (PicoPip Ag392, Biotrack, http://www.biotrack.co.uk/; 1.18 g, which was 4.4% of the mean and 5.1% of the smallest bird’s body mass). Briefly, the feathers above the uropygial gland of the bird were trimmed (short feather shafts left), and the transmitter was glued to the skin and shafts with Loctite® super glue. This technique is fast and has fewer behavioral effects compared with harness or implant techniques, and the transmitters drop off within a few weeks as the feathers regrow (reviewed by Warnock and Takekawa 2003). We took a small (ca. 50 µl) blood sample from a brachial vein for sexing, weighed each bird (to the nearest 0.1g) using a digital balance, and measured tarsus, culmen, and total head (to the nearest 0.1mm) with calipers and measured wing (to the nearest 0.5mm) with a ruler.

### Monitoring of incubation

Nests were found by systematically searching the tundra and by observing the behavior of birds flushed during laying or incubation (Sandercock and Gratto-Trevor 1997). The start of incubation and hatching was estimated by laying date for clutches found during laying and by measuring the height and inclination of the eggs floated in water for clutches found complete. This floating technique is based on the fact that eggs lose weight as the embryo develops: freshly laid eggs sink to the bottom of a water column and lay horizontal; as eggs develop, they rise with their blunt end and eventually float on the water surface (Liebezeit et al. 2007; median estimation of all floated eggs within the nest was used). Each nest was visited at the estimated hatching date to capture both parents to estimate their condition; to measure, bleed, and ring the chicks; and to determine the fate of the nest.

Incubation was monitored using a custom-made radio frequency identification device (RFID; designed by Calima Engineering, http://www.calima.de, in cooperation with the Max Planck Institute for Ornithology) in combination with a temperature probe (similar method used by Reneerkens et al. 2011). A thin antennae loop (ø 9cm) was fitted into the nest cup and connected to a datalogger approximately 0.5 m outside of the nest (Supplementary Picture S2a–c). This system registered the identity of a tagged bird on the nest every 5 s throughout the incubation period (for technical details, see legend in Supplementary Picture S2). To determine whether a bird was incubating, independent of the RFID reader, a minute external temperature probe (ø 2.5mm, 0.2 °C accuracy; Talk Thermistor, PB-5005-0M6) was placed in the middle of the nest between the 4 eggs and connected to a temperature logger (Tinytag Talk 2, TK-4023, Gemini Data Loggers, www.tinytag.info) placed 0.5 m outside of the nest. The probe was in level with the tops of the eggs (Supplementary Picture S2d) and secured with a toothpick. The logger recorded the temperature every 2min for the entire incubation period.

For 8 nests, the incubation behavior was also monitored by a video-recording system (custom designed by Jan Petrů, Czech Republic). An external lens (ø 2cm, length 4cm) and a microphone (ø 0.75cm, length 2.2cm) were positioned 1–3 m from the nest and were connected to the recorder hidden in the vegetation 5 m away from the nest. The recorder was supplied by a 12-V, 31-Ah, or 44-Ah battery hidden another 5 m away, allowing continuous recording for 2–4 days.

### Monitoring of egg incubation temperatures

To determine whether females and males differed in egg incubation temperatures, in 14 nests, instead of adding an external temperature probe, one egg was replaced with a solid egg (from PVC-U, painted to resemble a sandpiper egg; Supplementary Picture S3) with a high-resolution MSR® temperature probe (0.2 °C accuracy) positioned just under the egg surface. The fake egg was secured in the nest with a pin, and the probe was connected to an MSR® 145 datalogger (MSR® Electronics GmbH, Switzerland, http://www.msr.ch/en/) positioned outside the nest. Temperature was logged every 5 s throughout the incubation period. The fixed position of the probe in the fake egg and the fixed position of the fake egg in the nest allowed us to compare the within-nest sex differences in incubation temperatures.

### Disturbance

Data collection would be impossible without us walking through the study area. As a consequence, the birds were disturbed. To control for this disturbance, each field-worker carried a GPS (Garmin, Oregon 450) that recorded the person’s position whenever he/she moved 10 m within the study site. This allowed us to calculate the distance between each person and each nest at a given time. The probability of an incubating bird leaving the nest was under 10% whenever the closest person to the nest was further than 210 m away (our unpublished data). Therefore, we defined the absence or presence of disturbance at a given nest and at any one time based on whether a field-worker was present within 210 m of that nest.

### Tundra temperatures

The surface tundra temperature was recorded next to each nest in vegetation similar to that surrounding the specific nest cup. Two types of loggers were used: the MSR® 145 at the nests with a fake egg (recording interval 5 s; Supplementary Picture S2c and d) and the HOBO Pendant® Temperature Data Logger (0.47 °C accuracy, UA-002-64, Onset Computer Corporation, http://www.onsetcomp.com/) at all other nests (recording interval 1min). The housing of the MSR and HOBO logger differs in color. This could in principle affect the temperature recordings. However, both loggers recorded similar temperatures when placed next to each other (details are not presented), and the potential differences did not affect the extraction of incubation data (discussed in the next section).

### Extraction of incubation behavior

Egg temperatures were used to discriminate between incubation and nonincubation periods as described in detail in Supplementary Figure S4. Briefly, constant incubation temperatures higher than tundra temperatures were interpreted as continuous incubation; the start of incubation was determined from a steep increase, the interruption of incubation from a steep decrease in nest temperature (Supplementary Figure S4; see also Fig. 2 in Reneerkens et al. 2011). We automated the procedure using an R-script and validated the method by comparing the assigned incubation with plots of the raw data (Supplementary Figure S4) and with the video recordings.

The temperature-based determination of incubation/nonincubation was overlaid with the RFID data, which allowed assigning each incubation bout to a parent (Supplementary Figure S4). Subsequently, the length of each incubation bout was extracted as the total time allocated to a single parent. The constancy of incubation was calculated as the percentage of time a bird actually incubated within a given incubation bout (i.e., sat tightly on the eggs as opposed to egg rolling, nest maintenance, or being off the nest). The exchange gap duration was defined as the time between the departure of one parent and the return of the other parent.

### Timing of incubation-related events

The visualization of the raw RFID and temperature recordings allowed us to pinpoint the precise timing of desertion, depredation, or hatching; therefore, we adjusted the field data accordingly.

### Statistical analyses

R, version 2.15.2 (R Development Core Team 2012), was used for statistical analyses and the lme4 package (Bates and Maechler 2010) for the mixed-effects modeling.

#### Quality and amount

Sex differences in quality of incubation (incubation temperature and constancy) and amount of incubation (length of incubation bouts and exchange gaps) over the incubation period were tested by generalized linear mixed effect models (GLMMs) with the incu-bation feature as the dependent variable and with sex in interaction with day of incubation as fixed effects. Potentially confounding variables were added as fixed effects: disturbance (0, 1), start of incubation within the season (in interaction with day of incubation), body mass and size (both sex centered), and whether the bird carried a radio transmitter (0, 1). Culmen length was used as a proxy for body size (Ashkenazie and Safriel 1979b); culmen correlates with other body size measures (in our study, Pearson correlation coefficients [95% confidence interval {CI}]; tarsus: *r* = 0.51 [0.35–0.64], *t*_97_ = 5.9, *P* < 0.0001; total head: *r* = 0.87 [0.82–0.91], *t*_98_ = 17.9, *P* < 0.0001; wing: *r* = 0.5 [0.34–0.64], *t*_98_ = 5.7, *P* < 0.0001). All predictors (except sex) were mean centered (Schielzeth 2010). Incubation temperatures were *z*-transformed (mean centered and standard deviation scaled) within the nest and thus were made comparable between nests. The distribution of incubation constancy was normalized by arcsine transformation. The incubation constancy model was also controlled for the type of temperature probe (inside fake egg [0] or between the eggs [1]). The bout length model investigated also the sex-specific effect of the length of the previous (partner’s) incubation bout (off-nest bout of the focal bird) on the length of the current bout. The random structure of the models contained nest as a random intercept and *z*-transformed day of incubation as random slope. To follow current recommendations (Simmons et al. 2011), the Supplementary Tables report simple GLMMs without covariates. The results of all GLMMs include adjusted approximations of *P* values based on multiple comparisons (simultaneous inference) of predictors using the glht function from the multcomp package (Hothorn et al. 2008).

#### Timing

To investigate whether females and males incubate during different (cold—unfavorable for foraging—or warm—favorable for foraging) parts of the day, incubation period, or season (i.e., defined as start of incubation within the season), the following procedure was applied. We sampled the entire data set of approximately 8.4 million per 5-s recordings of incubation; an autocorrelation of the data points was avoided by stratifying the sample to 0.025% incubation data points per nest, with points at least 2.5h apart from each other. The sample was limited to the usable data (e.g., bouts with hatching or nests with only 4 incubation bouts were excluded; details are in the Sample sizes section). Binary coding was created (female incubation = 1 and male incubation = 0). The 5000 iterations of this process generated data sets with median (range) sample size of 1722 (1680–1768) incubation data points. These data were used as the binomial dependent variable in subsequent GLMM. We overcame the circular properties of time by converting it to radians and decomposing it into 2 linear variables: sin(rad) and cos(rad). Both sin(rad) and cos(rad) were entered in the model as explanatory variables in a 3-way interaction with the day of the incubation period and start of incubation within the season. Nest was included as a random intercept, and sin(rad) and cos(rad) were included as random slopes. The reported results of this exercise are summaries of the 5000 iterations (CIs are nonparametric).

### Sample sizes

The aim was to follow the entire breeding population on the study site for the entire incubation period. In total, we found 83 nests. We acquired the mass of both parents from 51 nests and morphometric measurements for both parents from 50 of those nests. Twenty-one nests were depredated and 3 nests deserted before or shortly after initiation of data collection; one nest was excluded because it was used to test the monitoring system (increased disturbance); an additional 7 nests were excluded because they were found only close to hatching. Thus, the basic data set, used for further analyses, consisted of over 8.9 million per 5-s readings from 51 nests, with median (range) = 20.4 (6.7–31.3) incubation days/nest. For these nests, we excluded the first 2 incubation bouts after first parent catching, the bout where the nest was deserted or depredated, and all bouts that ended within 6h before the start of hatching. We further excluded all nests with less than 4 incubation bouts (*N* = 3). Thus, the final data set consisted of 887 incubation bouts from 48 nests (median [range] = 18 [4–42] bouts/nest; median [range] start of incubation = 7 [1–26] June). Bouts for which the temperature recordings were missing were excluded from the analysis of incubation constancy and exchange gaps, leaving a total of 809 incubation bouts from 47 nests (median [range] = 16 [4–42] bouts/nest). Data sets were further reduced in the mixed models because only birds for which morphometric measurements were available were included. The data set for the models on the constancy of incubation consisted of 762 incubation bouts from 47 nests (median [range] = 15.5 [3–42] bouts/nest). This data set was further reduced in the model on the length of incubation bouts because to investigate the effect of the previous (partner’s) bout, a continuous data set is required. Hence, we excluded the first incubation bout in each nest (the previous bout is absent) and nests where only one bird was measured. This left 729 incubation bouts from 39 nests (median [range] = 18 [3–41] bouts/nest). The data set for the model on the incubation temperatures (only nests with a fake egg) consisted of 307 incubation bouts from 14 nests (median [range] = 21.5 [9–42] bouts/nest). The data sets are available from the Dryad Digital Repository (http://doi.org/10.5061/dryad.nh8f0).

## RESULTS

### Quality of incubation

**Figure 1:**
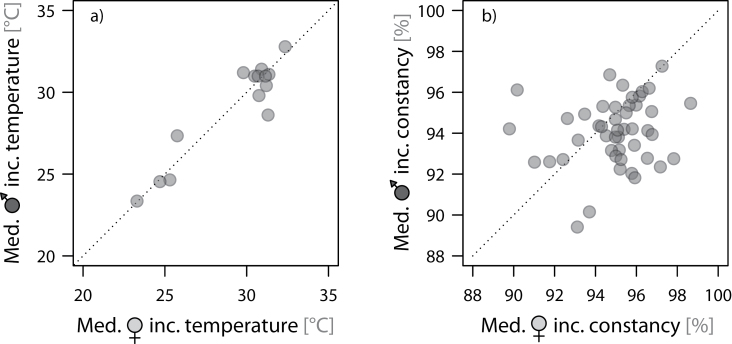
Within-pair differences in (a) the median fake-egg incubation temperature and (b) the median constancy of incubation. Each dot represents 1 nest. Incubation temperatures are not comparable between nests because the position of the temperature probe within the fake egg and of the fake egg within a nest is not exactly the same in each nest. Incubation constancy is comparable between nests.

Incubation temperatures did not change systematically over the incubation period or over the season (start of incubation within season) and on average did not differ between females and males (Figure 1a and Table 1). Within-nest variance accounted for 78% of overall phenotypic variance (Table 1). Body mass and size of the bird had no effect on incubation temperature nor did the attachment of a radio transmitter (Table 1).

**Table 1:** Model (GLMM) estimates of median *z*-transformed incubation temperature per incubation bout in relation to sex and incubation period with disturbance, presence of radio tag, body mass, culmen length, and start of incubation within the season as confounding variables

Fixed effects	Estimate	95% CI	*P*
(Intercept)	0.155	(−0.031, 0.342)	0.18
Disturbance	0.02	(−0.161, 0.201)	1
Radio tag	0.219	(−0.036, 0.474)	0.15
Culmen	−0.045	(−0.154, 0.065)	0.93
Body mass	−0.014	(−0.077, 0.048)	1
Start of incubation	0.004	(−0.039, 0.047)	1
Day of incubation	0.016	(−0.018, 0.049)	0.86
Sex (male)^a^	0.046	(−0.096, 0.188)	0.98
Sex × Day of incubation	0.002	(−0.027, 0.031)	1
Start of incubation × Day of incubation	−0.001	(−0.008, 0.006)	1
Random effects	Variance		
Nest (intercept)	0.0337		
*z*-Transformed (day of incubation)	0.0177		
Residual	0.1864		

*N* = 307 median *z*-transformed incubation temperatures per incubation bout from 14 nests. Fixed effects, except sex, were mean centered (culmen and body mass were centered within each sex). Median incubation temperatures were calculated from raw incubation temperature-values *z*-transformed within each nest. Results of the model without confounding variables are presented in Supplementary Table S1.

^a^Relative to female.

Overall, the median constancy of incubation within an incubation bout was 94.9% (range: 43–100%; *N* = 809 incubation bouts from 47 nests). Constancy of incubation within incubation bouts did not change systematically over the incubation period or over the season, but on average females had 0.9% higher incubation constancy per incubation bout than males (Figure 1b and Table 2). This corresponds to approximately 6min of longer incubation bouts by females (given a median bout length of 11.45h). Within-nest variance accounted for 77% of overall phenotypic variance (Table 2). Size and body mass of the bird had no effect on its incubation constancy nor did the attachment of a radio transmitter (Table 2).

**Table 2:** Model (GLMM) estimates of incubation constancy per incubation bout (arcsine transformed) in relation to sex and incubation period with disturbance, type of temperature probe, presence of radio tag, body mass, culmen length, and start of incubation within the season as confounding variables

Fixed effects	Estimate	95% CI	*P*
(Intercept)	1.342	(1.328, 1.355)	<0.0001
Disturbance	−0.023	(−0.042, −0.005)	0.0039
Temperature probe type	0.003	(−0.024, 0.031)	1
Radio tag	−0.003	(−0.029, 0.022)	1
Culmen	−0.003	(−0.014, 0.008)	0.99
Weight	−0.001	(−0.007, 0.004)	1
Start of incubation	−0.001	(−0.003, 0.001)	0.93
Day of incubation	−0.001	(−0.005, 0.004)	1
Sex (male)^a^	−0.02	(−0.036, −0.004)	0.0042
Sex × Day of incubation	0.003	(0, 0.006)	0.16
Start of incubation × Day of incubation	0	(−0.001, 0)	1
Random effects	Variance		
Nest (intercept)	0.0001		
*z*-Transformed (day of incubation)	0.0017		
Residual	0.0057		

*N* = 762 incubation constancies per incubation bout from 47 nests. Fixed effects, except sex, were mean centered (culmen and body mass were centered within each sex). Results of the model without confounding variables are presented in Supplementary Table S2.

^a^Relative to female.

In short, these results indicate that overall the quality of incubation varied little over the course of incubation and played a minor role in sex-specific investment.

### Amount of incubation

**Figure 2:**
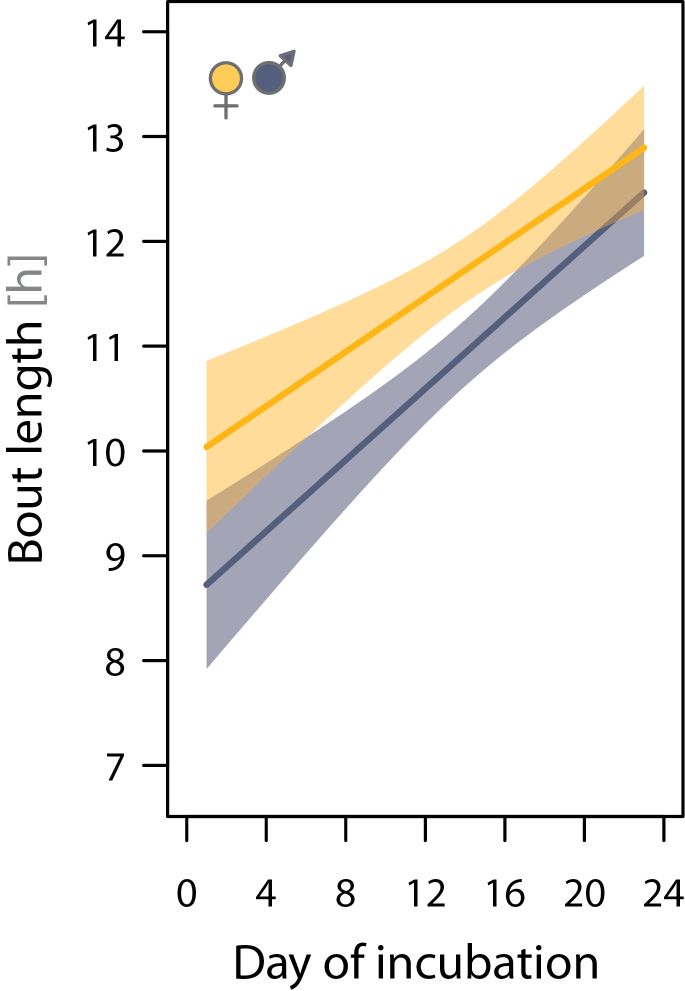
Increase in incubation bout length over the incubation period. The solid lines represent the model fit, and the shading represents the 95% CIs. Model results are presented in Table 3, and the distribution of the raw data is depicted in Supplementary Figure S5.

**Figure 3:**
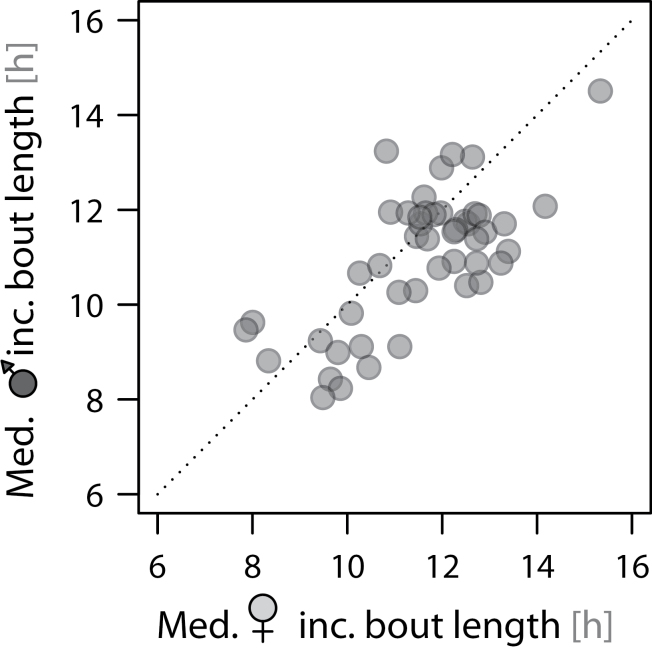
Between- and within-pair differences in the median length of incubation bouts. Each dot represents 1 nest. The correlation of the median bouts between sexes: Pearson correlation coefficient (95% CI): *r* = 0.71 (0.53–0.83), *t*_46_ = 6.8, *P* < 0.0001.

**Figure 4:**
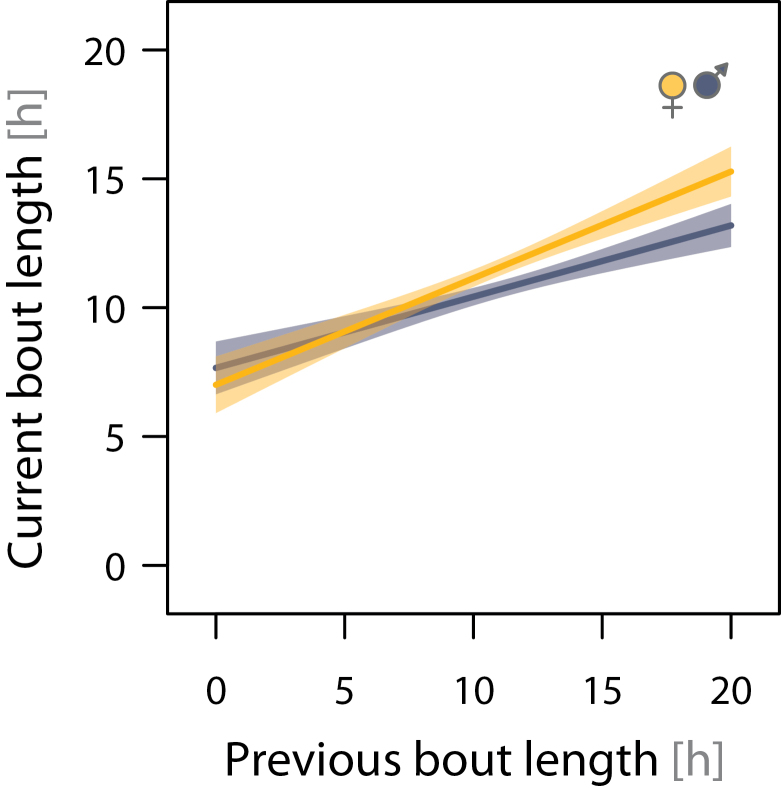
Positive relationship between incubation bout length and previous bout length (partner’s bout; off-nest bout of the focal bird). The solid lines represent the model fit, and the shading represents the 95% CI. Model results are presented in Table 3.

The median length of all incubation bouts was 11h 27min (range: 3.4 min–18.2h; *N* = 887 bouts from 48 nests). Bout length increased systematically over the incubation period (by ca. 9min/day; Figure 2). The increase was consistent across nests (between-nest variation in the change of bout length over the incubation period accounted for less than 1.1% of the variance) but independent of the start of incubation within the season and independent of sex (Figure 2 and Table 3). On average, females incubated 51min (95% CI: 26–76min) longer per incubation bout than males (Figures 2 and 3 and Table 3); thus, the median proportion of female incubation over the entire incubation period was 51.4% (range: 45.5–57%; *N* = 48 nests). After controlling for sex differences, incubation bout length did not depend on body mass or size and was unaffected by an individual wearing a radio tag or not (Table 3). Despite the general trend, in 16 of 48 nests (33%), the median bout length of the female was shorter than that of the male (Figure 3). Incubation bout length was positively correlated among pairs (Figure 3), indicating that if one parent had a longer median incubation bout than that of the rest of the population, so had its partner. This partner matching is also present within the pairs’ incubation period: the length of the previous (partner’s) incubation bout (which is the off-nest bout of the focal bird) strongly predicted the length of the current incubation bout of both sexes (Figure 4 and Table 3).

**Table 3:** Model (GLMM) estimates of incubation bout length (in minutes) in relation to sex, incubation period, and length of the previous bout with disturbance, presence of radio tag, body mass, culmen length, and start of incubation within the season as confounding variables

Fixed effects	Estimate	95% CI	*P*
(Intercept)	692.2	(663.9, 720.5)	<0.0001
Disturbance	38.9	(9.4, 68.4)	0.002
Radio tag	8	(−39.8, 55.9)	1
Culmen	−2.6	(−20.6, 15.4)	1
Body mass	−0.9	(−10.5, 8.7)	1
Previous bout length	0.4	(0.3, 0.6)	<0.0001
Start of incubation	2.2	(−1.8, 6.1)	0.76
Day of incubation	7.8	(2.8, 12.8)	0.0001
Sex (male)^a^	−50.9	(−76.3, −25.5)	<0.0001
Sex × Previous bout	−0.1	(−0.3, 0)	0.29
Sex × Day of incubation	2.4	(−3.5, 8.4)	0.95
Start of incubation × Day of incubation	0	(−0.6, 0.6)	1
Random effects	Variance		
Nest (intercept)	2032		
*z*-Transformed (day of incubation)	165		
Residual	13 779		

*N* = 729 incubation bouts from 39 nests. Fixed effects, except sex, were mean centered (culmen and body mass were centered within each sex). Results of the model without confounding variables are presented in Supplementary Table S3.

^a^Relative to female.

**Figure 5:**
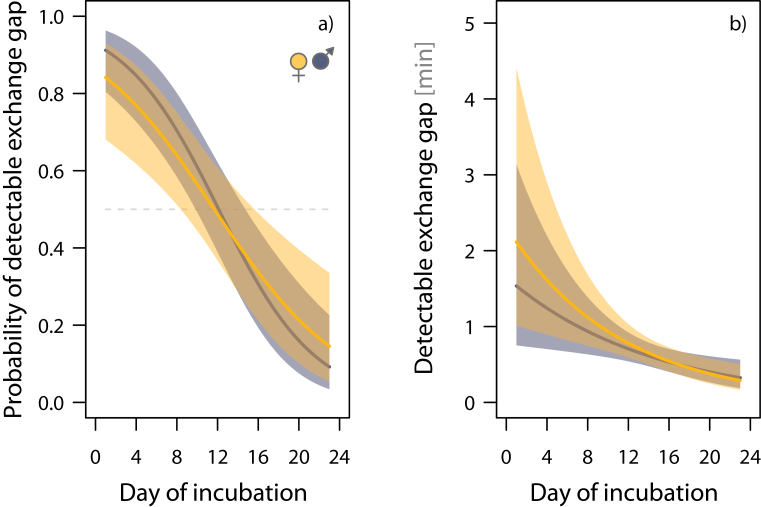
Decrease in (a) the probability of a detectable exchange gap (≥5 s) and (b) the length of detectable exchange gaps over the incubation period. The solid lines represent the model fit, the shading represents the 95% CI, and the horizontal dashed line in (a) represents the equal probability. Model results are presented in Tables 4 and 5, and the distribution of the raw data is depicted in Supplementary Figure S6.

There was no detectable exchange gap (<5 s) during 51% of the exchanges (*N* = 809 exchanges at 47 nests); the median length of all detectable exchange gaps was 35 s (range: 5 s–6.7h; *N* = 399 detectable gaps from 44 nests). Both the probability of a detectable exchange gap and the length of detectable exchange gaps decreased over the incubation period, irrespective of the sex of the exchanging bird (Figure 5 and Tables 4 and 5). Within-nest variance in the length of detectable exchange gaps accounted for 80% of the overall phenotypic variance (Table 5).

**Table 4:** Model (binomial GLMM) estimates of the probability of a detectable exchange gap (≥5 s) in relation to sex and incubation period with disturbance, type of temperature probe, presence of radio tag, body mass, culmen length, and start of incubation within the season as confounding variables

Fixed effects	Estimate	95% CI	*P*
(Intercept)	−0.124	(−0.848, 0.6)	1
Disturbance	−0.046	(−0.674, 0.583)	1
Temperature probe type	−1.644	(−3.518, 0.23)	0.13
Radio tag	0.049	(−1.721, 1.819)	1
Culmen	0.033	(−0.356, 0.422)	1
Body mass	0.1	(−0.112, 0.313)	0.87
Start of incubation	−0.065	(−0.177, 0.046)	0.64
Day of incubation	−0.155	(−0.269, −0.041)	0.0014
Sex (male)^a^	0.064	(−0.455, 0.585)	1
Sex × Day of incubation	−0.054	(−0.166, 0.058)	0.85
Start of incubation × Day of incubation	−0.004	(−0.019, 0.012)	1
Random effects	Variance		
Nest (intercept)	1.72		
*z*-Transformed (day of incubation)	0.38		

*N* = 762 exchanges from 47 nests. Fixed effects, except sex, were mean centered (culmen and body mass were centered within each sex). Results of the model without confounding variables are presented in Supplementary Table S4.

^a^Relative to female.

**Table 5:** Model (GLMM) estimates of detectable exchange gap duration (in seconds, log transformed) in relation to sex and incubation period with disturbance, type of temperature probe, presence of radio tag, body mass, culmen length, and start of incubation within the season as confounding variables

Fixed effects	Estimate	95% CI	*P*
(Intercept)	3.894	(3.481, 4.306)	<0.0001
Disturbance	−0.113	(−0.592, 0.366)	1
Temperature probe type	0.579	(−0.295, 1.453)	0.47
Radio tag	0.007	(−0.738, 0.753)	1
Culmen	−0.035	(−0.333, 0.264)	1
Body mass	−0.054	(−0.201, 0.093)	0.97
Start of incubation	0.037	(−0.026, 0.1)	0.64
Day of incubation	−0.09	(−0.167, −0.013)	0.01
Sex (male)^a^	−0.114	(−0.512, 0.284)	0.99
Sex × Day of incubation	0.02	(−0.063, 0.103)	1
Start of incubation × Day of incubation	−0.003	(−0.07, 0.064)	1
Random effects	Variance		
Nest (intercept)	0.305		
*z*-Transformed (day of incubation)	0.139		
Residual	1.815		

*N* = 385 exchange gaps from 44 nests. Fixed effects, except sex, were mean centered (culmen and body mass were centered within each sex). Results of the model without confounding variables are presented in Supplementary Table S5.

^a^Relative to female.

Taken together, these results indicate that the amount of incubation changed over the incubation period and was female biased.

### Timing of incubation: general incubation pattern

The median proportion of female incubation within the cold period was 72.6% (range: 0–100%; *N* = 356 days from 48 nests). Thus, overall, females incubated more during the cold period of the Arctic day (i.e., when the tundra temperatures were on average below overall median tundra temperature, roughly between 21:30 and 09:30), whereas males incubated more during the warmer period when foraging conditions were more favorable.

**Figure 6:**
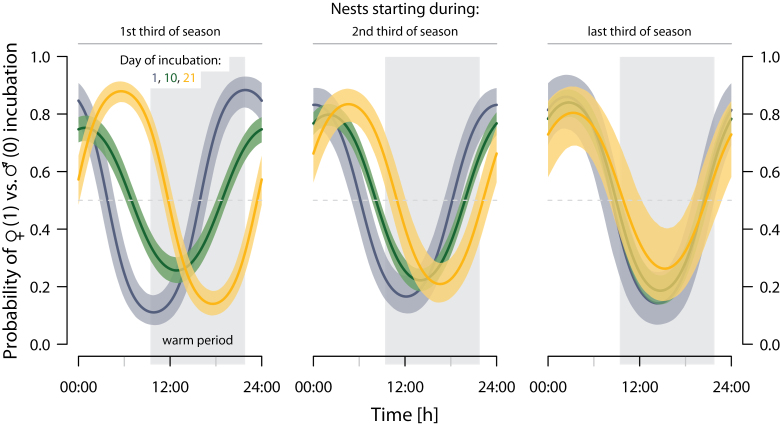
Shift in the probability of female (vs. male) incubation during specific time of a day over the 21-day incubation period and with respect to the start of incubation within the season. Color lines represent the mean predicted probability of 5000 GLMMs for the 1st (dark blue), the 10th (green), and the 21st (yellow) day of incubation; color shadings represent the nonparametric CIs that contain 95% of the 5000 fits. Left panel: predictions from 6 June (first nest started on 1 June); middle panel: 13 June; right panel: 19 June. The distribution of the nests across the season is in Supplementary Figure S7. The horizontal dashed line indicates an equal share of incubation, and the gray shaded rectangle represents the time when the tundra temperatures were on average above overall median tundra temperature, that is, the warmer period of the Arctic day. Nest-specific incubation patterns for all 48 nests are in Supplementary Actograms.

The timing of incubation, however, shifted over the breeding season and as incubation progressed (Figure 6 and Table 6). In early nests, female incubation shifted from evening–night to night–morning over the incubation period (Figure 6, left panel: 1st third of season). This shift (of ca. 7.5h) weakened over the season (Figure 6, middle panel: 2nd third of season) and became absent in late nests (Figure 6, right panel: last third of season). Note that we had fewer nests starting in the second half of the season and running for more than 10 days (Supplementary Figure S7).

In short, these results show incubation patterns that are potentially specific to different parts of the breeding season.

**Table 6:** Summary of 5000 model (binomial GLMM) estimates of the probability that the female (vs. male) incubates in relation to time of a day, incubation period, and start of incubation within the season

Fixed effects	Estimate	95% CI	Number of iterations (*P* < 0.05)	*P*
(Intercept)	0.059	(−0.021, 0.14)	318	0.94
cos(time)	0.915	(0.757, 1.079)	4998	0.0004
sin(time)	0.755	(0.61, 0.906)	5000	0
Day of incubation	0.007	(−0.01, 0.024)	101	0.98
Start of incubation	0.006	(−0.006, 0.018)	133	0.97
Start of incubation × Day of incubation	0	(−0.002, 0.003)	22	1
cos(time) × Day of incubation	−0.066	(−0.098, −0.034)	4494	0.10
cos(time) × Start of incubation	0.02	(−0.003, 0.044)	0	1
cos(time) × Day of incubation × Start of incubation	0.003	(−0.002, 0.008)	284	0.94
sin(time) × Day of incubation	0.109	(0.079, 0.139)	5000	0
sin(time) × Start of incubation	0.026	(0.006, 0.046)	0	1
sin(time) × Day of incubation × Start of incubation	−0.012	(−0.017, −0.007)	4928	0.0144
Random effects	Variance	95% CI		
Nest (intercept)	0	(0, 0)		
cos(time)	4.67	(3.6, 6.0)		
sin(time)	2.37	(1.84, 3.04)		

Median (range) *N* = 1722 (1680–1768) incubation data points from 48 nests. Time of a day (in radians) was decomposed into sin and cos; the remaining fixed effects were mean centered. Estimates are means, and their 95% CIs (nonparametric) are 0.025 and 0.975 quintiles, of the fixed effect estimates of 5000 GLMMs. Variances are mean values, and 95% CIs are 0.025 and 0.975 quintiles, of the random effects from 5000 GLMMs. The median (range) partial autocorrelation coefficient of GLMMs was 0.038 (0.001–0.074) in lag 1 and −0.264 (−0.311 to −0.214) in lag 2.

### Timing of incubation: different incubation patterns

The observed variation in incubation patterns, within season or between nests (random slopes of sin[time] and cos[time] accounted for all variance; Table 6), has different consequences for female–male division of incubation.

**Figure 7:**
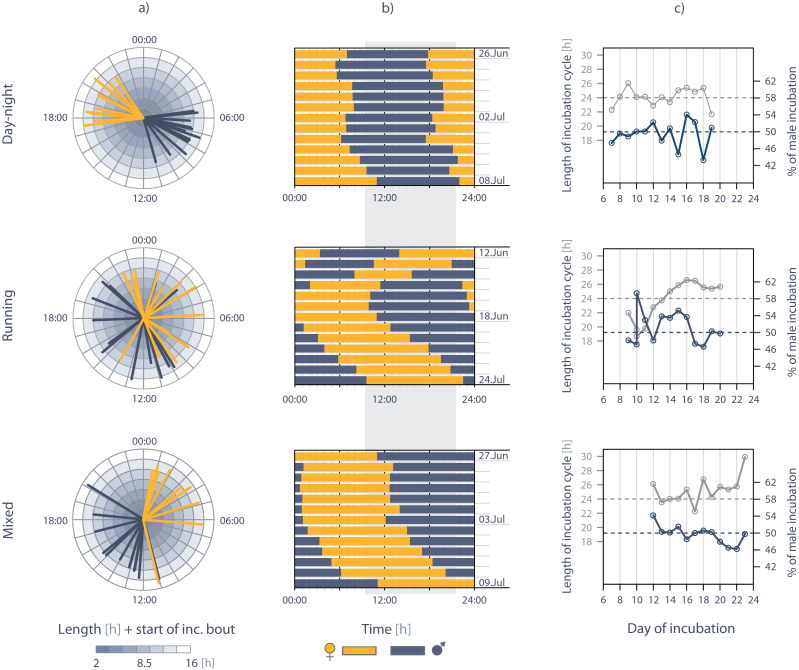
Examples illustrating the variation in the division of amount and timing of incubation in semipalmated sandpiper pairs. Each row represents 1 specific nest, illustrating a day–night pattern, a running pattern, and a mixed pattern (see text for details). (a) Division of incubation within a day (yellow lines = female, dark blue lines = male; the position of each line marks the start of an incubation bout, the length of the line reflects incubation bout length). (b) Visualization of incubation bouts of females and males across the incubation period (gray shading = approximate warmer period of the day, i.e., the time when the tundra temperatures were on average above median tundra temperature). (c) Changes in the length of the incubation cycle (i.e., the sum of the female and subsequent male bout length; solid gray line, left *y* axis) and male share of incubation (i.e., the percentage of male incubation within each cycle; solid dark blue line, right *y* axis) across the incubation period. The dashed lines indicate a 24-h cycle (gray, left *y* axis) and equal share of incubation (dark blue, right *y* axis). For illustration, the early or late incubation period is excluded, such that all 3 nests show the same part of the incubation period.

At one extreme were nests where the length of the incubation cycle (female + male incubation bout) roughly followed a 24-h period (Figure 7, day–night). These nests showed a distinct division of female and male incubation within a day throughout most of the incubation period; even if parents divided the amount of incubation roughly equally (in the example in Figure 7, the male incubated 49.5% of the time), one parent incubated during the night (i.e., the colder part of the 24-h day) and the other during the day (i.e., the warmer part of the Arctic day; in the example in Figure 7, 81% of the male incubation occurred during this time).

At the other extreme were nests where the length of the incubation cycle substantially deviated from a 24-h period (Figure 7, “running”). As a result, the time of day when parents exchanged became progressively earlier (usually during the 1st half of incubation) or later (usually during the 2nd half of incubation) as incubation advanced (Figure 7b). This allowed both parents to experience similar incubation/off-nest conditions but during different days within the incubation period. Unlike the day–night extreme, both pair members incubated during the warmer part of the day; in the example depicted in Figure 7, the total male share of incubation was 51%, and 49% of his incubation fell in the warmer period of the Arctic day.

In between these extremes were nests that ran moderately over the incubation period, so that the general day–night (cold–warm) division between partners remained, or nests where the periodicity was close to 24h during part of the incubation, but larger or smaller than 24h during another part (e.g., Figure 7, “mixed”; nest-specific incubation patterns for all 48 nests are depicted in Supplementary Actograms).

## DISCUSSION

Using a continuous monitoring system, we quantitatively described incubation patterns in a population of semipalmated sandpipers breeding in the high Arctic. The incubation patterns and hence sex-specific costs varied considerably between pairs. Our results show that the amount of incubation (bout length) generally increased over the incubation period, causing a shift in the daily timing of incubation; the degree of this shift seemed dependent on the time within the season. We also found that incubation bout lengths were positively correlated between pair members. The exchange gaps became shorter or disappeared over the incubation period, suggesting that pair members became better synchronized. The quality of incubation varied little over the course of incubation and was similar for females and males. Females incubated on average longer than males; thus, in the absence of other data, one would conclude that in this species, females invested more in incubation than males. However, our results further show that females incubated more often during the colder part of the day (night) when foraging efficiency is expected to be lower and predation pressure may be different than during the warmer part of the day (day). This suggests that the female-biased amount of incubation might be offset by a more favorable timing of incubation. We discuss our findings in relation to sex-specific costs of care and resolution of the conflict between the parents.

### Possible explanations for sex differences in incubation

Why did female semipalmated sandpipers incubate longer and more constantly than males? Early in incubation, females might incubate longer than males because males might spend more time defending their territories. However, this interpretation is unlikely because male incubation bouts were consistently shorter across the entire incubation period (Figure 2). Alternatively, females might be able to sit longer and more constantly because they are larger and can carry more resources than males and therefore could afford the costs of incubating longer (i.e., females might experience the same relative costs of incubation as males). However, females seem to have higher energy expenditure during incubation than males (Ashkenazie and Safriel 1979b). Also, size dimorphism appears an unlikely explanation because body size and mass did not explain much of the variation in the length of incubation bouts or in incubation constancy among females or males (Tables 2 and 3). The longer incubation bouts and higher incubation constancy of females might be directly related to the sex difference in timing of incubation for the following reasons.

First, the bird incubating during the colder part of the Arctic day (most often the female) may be forced to sit tighter on the eggs to prevent their cooling or to minimize the detectability of the nest if predators are more abundant or more active, both leading to higher incubation constancy. Indeed, including time of day in the constancy model reduced the sex effect (Supplementary Table S6). However, predatory pressure is an unlikely explanation for this effect, at least in our study site, because predation events occurred predominantly during the day (Supplementary Figure S2).

Second, females may incubate longer because during incubation, they are less energy constrained. The availability of arthropods, the main shorebird prey, strongly correlates with ambient temperatures (Corbet 1966; Danks and Oliver 1972; Schekkerman et al. 2003; Tulp and Schekkerman 2008), making foraging easier during the warmer part of the Arctic day. Furthermore, because diel fluctuations in wind speed are minimal (Supplementary Figure S1), being off nest during the warmer “day” will be energetically less demanding than being off nest during the colder “night.” These demands might be extreme because energy expenditure in high-Arctic breeding shorebirds often reaches ceilings of sustainable energy turnover rates (Piersma et al. 2003). Thus, foraging during the colder period will reduce the net energy intake rate of the feeding bird as prey availability is lower and the need for thermoregulation is higher. Hence, incubating during the cold part of the day and foraging during warm periods seem advantageous. Yet, it is unclear why females are more likely to capitalize on this advantage.

An unanswered question is whether the sex difference in the amount of incubation is related to the level of brood care. Females of this species are more likely to desert the brood earlier than the males (Ashkenazie and Safriel 1979a; Hicklin and Gratto-Trevor 2010). Thus, the variation in the timing of desertion may be linked to the investment during incubation, that is, females that dedicate more to incubation may tend to desert the brood earlier (e.g., because they have depleted their resources) or later (e.g., because they may be high-quality females that generally can invest more in the brood).

### Variation in incubation patterns: current and previous findings

There was relatively little between-nest variation in the amount and quality of incubation (Tables 1–3), but pairs varied considerably in their timing of incubation. This resulted in unexpected variation in incubation patterns (Figure 7). Perhaps the most important difference between these patterns, in terms of costs of incubation, is that only in the “running” pattern, both parents could forage during the warmer parts of the day or be exposed to similar risk of predation, at least on some days.

The running pattern, however, is not the only possible scenario that would lead to a relatively equal division of incubation during the colder part of the day. Theoretically, parents could have shorter incubation bouts (e.g., of a few hours) allowing both of them to forage when it is more efficient. However, this would lead to more frequent exchanges and might be counter selected if it increases predation risk (Smith et al. 2012). Alternatively, parents could keep regular 24-h incubation cycles but with changeovers that would allow each partner to experience part of the colder and part of the warmer period of each day.

It is difficult to assess whether the observed variation in incubation patterns is also present in other species because continuous data throughout the incubation period are scarce. Moreover, the literature is dominated by studies on species with incubation bouts lasting >24h (albatrosses, e.g., Weimerskirch et al. 1986; Weimerskirch 1995; penguins, e.g., Davis 1982; Weimerskirch et al. 1992; Gauthier-Clerc et al. 2001; and petrels, e.g., Chaurand and Weimerskirch 1994), where timing of incubation within a day does not play a role. In the remaining species, the available (noncontinuous) data suggest that a day–night incubation pattern is typical for day–night environments (reviewed by Skutch 1957). Continuous laboratory observations confirmed female-biased night incubation in masked doves, *Oena capensis* (Hoffmann 1969), and in ring doves, *Streptopelia risoria* (Wallman et al. 1979; Ball and Silver 1983); the same may occur in other Columbiformes (Hoffmann 1969). In contrast, continuous incubation records of (radioactively tagged) black-legged kittiwakes, *Rissa tridactyla*, revealed varying incubation patterns, including “day–night,” and “running” (Coulson and Wooller 1984). In shorebirds (based on the recordings of few nests or days), both female-biased (e.g., Jehl 1973; Ward 1990) and male-biased (e.g., Byrkjedal 1985; Sullivan Blanken and Nol 1998; Currie et al. 2001) night incubation have been reported.

### Possible causes of variation in incubation patterns

The causes, consequences, and adaptive significance of the observed incubation patterns (Figure 7) await exploration.

Our results suggest that these patterns are to some extent season specific (Figure 6), that is, nests that started in the first half of the season tended to show a running pattern, whereas late nests more often showed a day–night pattern. To confirm this trend, a larger sample size is required, particularly for late-starting nests (Supplementary Figure S7). One possible explanation for the within-seasonal variation is that a different subset of birds is incubating later in the season (e.g., individuals that are of lower quality or that are renesting after a predation event). Alternatively, the within-seasonal variation may be influenced by the change in weather conditions from early to late breeding season.

Whether additional or different mechanisms drive the described patterns remains unclear. Here, we discuss how the patterns can arise through variation in response to external environmental cues, variation in individuals’ internal clock, or variation in the settlement of parental conflict among pair members over the amount and timing of incubation, or through a combination of these.

### 

#### External environmental cues

The typical day–night pattern (24-h cycle) may arise even under continuous light, when individuals use other external cues (e.g., light intensity or quality, temperature) as zeitgeber (Steiger et al. 2013). A running pattern may then arise if individuals are less sensitive to such subtle cues. Early experimental evidence suggests that incubation patterns may indeed be influenced by photoperiod: in carrier pigeons, domesticated *Columba livia*, pairs showed running or variable incubation patterns when exposed to continuous light (no fluctuations in light intensity), whereas pairs kept a day–night incubation pattern (with male incubation during the day, as under natural conditions) when exposed to a 12:12h light:dark cycle (Schmidt-Koenig 1958). The day–night pattern remained when parents experienced different darkness levels at night. However, the time of the exchanges varied more than during the strict day–night light regime (Schmidt-Koenig 1958). Thus, the observed variation in incubation patterns could reflect individuals that differ in their responsiveness to more subtle zeitgebers in the Arctic.

#### Internal clock

The observed variation in incubation rhythms (Figure 7) might also be linked to individual variation in the internal clock. Disruption or shifts in daily rhythms (e.g., due to changing light regimes) may lead to severe costs (Aschoff et al. 1971; Foster and Wulff 2005; LeGates et al. 2012). Therefore, if a specific zeitgeber (e.g., day–night, tide) drives the daily behavioral rhythm of individuals during most of their life, it might be advantageous for individuals to keep their rhythm also in an environment where the specific zeitgeber is absent. Because semipalmated sandpipers are predominantly tidal (Hicklin and Gratto-Trevor 2010), a running pattern might reflect the tide-bound internal clock of individuals (including the time when birds become hungry and get the urge to forage). Short female–male cycles (due to short incubation bouts) during early incubation might reflect the approximately 12.5-h cycle of low and high tide, whereas the long incubation cycle during late incubation might reflect 2 low–high tide cycles (25h). Keeping the shifting tidal pattern while incubating in the high Arctic, where food availability fluctuates with time of day, may be beneficial if it allows both parents to forage during the times of the day when food is most abundant. Hence, the observed variation in timing patterns could reflect individuals that differ in life history (i.e., outside the breeding season live in environments driven by different zeitgebers).

#### Settlement of parental conflict

The observed variation in incubation patterns can also reflect between-pair differences in behavioral rules that determine the length of incubation bouts or in negotiations among pair members. During continuous biparental incubation, only one parent at a time can be off nest (e.g., to feed), so one or both parents will need to adjust their individual schedules (e.g., feeding, resting) and possibly compromise their internal clock (discussed above). In migratory birds such as semipalmated sandpipers, pair members can be running on different rhythms (e.g., depending on migratory routes and timing of migration). We do not know whether parents use behavioral rules, such that one parent forces its internal rhythm on the other, or whether parents negotiate and synchronize toward a new rhythm, which then leads to a particular incubation pattern. However, our observations support some scenarios more than others, and we discuss 3 possible behavioral rules and a negotiation scenario.

First, the incubation patterns may arise from the rule “when the foraging partner comes back to the nest, the incubating bird goes” (come-and-go rule); the observed variation in the patterns may then reflect differences in the decision of the returning birds about when to return to the nest. In support of this rule, we found striking synchronization between partners; exchanges between incubating birds were usually instantaneous (81% of exchange gaps were shorter than 1min), despite large within-nest variation in incubation bout length (nearly 2h). However, the come-and-go rule fails to explain why synchronization increased over time (the occurrence and length of exchange gaps decreased over the incubation period; Figure 5). Also, our observations suggest that both birds may determine the bout length because 1) the off-nest bird is often foraging or resting up to several kilometers away (Ashkenazie and Safriel 1979a; our unpublished data) and has to return to the nest before the changeover (which is mostly immediate, see above), 2) the sitting bird does not always leave when the partner returns (and may even chase away the incoming bird; Ashkenazie and Safriel 1979a), and 3) the sitting bird sometimes leaves the nest before the partner returns (although rare, exchange gaps up to 6.7h occur). Thus, the simple come-and-go rule seems unlikely.

Second, the patterns may arise from the rule “when the foraging partner comes back to the nest, the incubating bird decides when to leave”; then the variation in the patterns may reflect individual differences in the decision to leave the nest when the partner returns. Although plausible, it remains unclear what factors influence this decision and how the off-nest bird knows when to return.

Third, the incubation patterns may arise due to energy constraints; variation in the patterns may then reflect variation in the birds’ condition. Under this “energy rule,” the off-nest bird may return whenever it has replenished its energy reserves, and the incubating bird may leave whenever its energy reserves have dropped to a certain threshold. This scenario has been supported by experimental data suggesting that a parent prolongs its incubation bout when the energetic demands during incubation are lower (Cresswell et al. 2003). But a similar experiment and reanalyses of Cresswell et al.’s (2003) data revealed or depicted no such relationship (Bulla M, Cresswell W, Valcu M, Rutten AL, Kempenaers B, unpublished data). Hence, although there is no doubt that energetic constraints play some role in determining incubation patterns in biparental incubators (Chaurand and Weimerskirch 1994), these constraints do not seem to fully explain the patterns.

Finally, the patterns could arise due to a form of negotiation between the pair members; variation in the observed patterns would then reflect different outcomes of the negotiations. Our results support the predictions of 2 game-theory models of biparental negotiations. In the first model, parents match their amount of care when they have partial information about the brood need; investment of one parent serves as a signal of the brood need to the other parent (Johnstone and Hinde 2006). As predicted, we found that bout lengths of partners were positively correlated. However, these models seem to apply more to offspring feeding; whether incubating parents have only partial information about the need of their eggs seems unlikely. Also, the model does not explicitly consider repeated bouts of investment.

The second model explicitly considers repeated bouts of investment and predicts an increase in the amount of care for both parents with consecutive bouts of investment (Lessells and McNamara 2011). We observed exactly that, as incubation progressed, bouts increased in length. The model further suggests that the amount of parental care will depend on the parent’s quality; the higher quality parent will deliver more care. The observed variation in incubation patterns between pairs may, thus, reflect pairs with parents of different quality or in different condition. This is possible, but at least individual body mass (measured once) and size (proxy for individual condition and quality; e.g., Peig and Green 2009) explained little of the variation in the length of incubation bouts. In addition, although the Lessells–McNamara model incorporates quality of care, it does not consider timing of care. It assumes that the cost function for a parent is the same in all bouts of investment. But our results indicate that the costs of individual investment might vary over time, for example, by changes in the timing of incubation relative to optimal foraging opportunities.

In sum, variation in the incubation patterns is unlikely a result of birds differing solely in their decision to return to the nest or in their condition. Although differences in the decision of incubating birds to leave the nest or negotiations seem more likely to explain the various incubation patterns, experimental evidence is missing. Our findings suggest that current game-theory models of biparental care are not yet directly applicable to biparental incubation because they do not explicitly consider amount, quality, and timing of care. Incorporating variation in the temporal cost of investment in these models might help understand the within-population variation in incubation patterns we described.

## CONCLUSIONS

The significance of our findings is 3-fold. First, our study provides a quantitative framework for future work on biparental care patterns. The framework allows quantification of both general trends and within-population variation (suggesting possibly different incubation strategies). Second, our results reveal variation in biparental incubation patterns, with possibly different consequences for sex-specific costs of care. This highlights the need to investigate not only the central tendency but also the variation in costs of parental care over time. Whether similar variation is also present in other species or systems (e.g., breeding under less extreme environmental conditions) remains unknown. Finally, although our study is limited to observations of incubation, that is, misses other forms of parental care (e.g., brood care), it demonstrates that focusing only on one aspect of care or on a short snapshot of care in time may bias our perception of costs of parental care and therefore may be insufficient for understanding parental conflict and its outcomes.

## SUPPLEMENTARY MATERIAL

Supplementary material can be found at http://www.beheco.oxfordjournals.org/

## FUNDING

This work was funded by the Max Planck Society.

## Supplementary Material

Supplementary Data
