# Supplementary material for: Biparental incubation patterns in a high-Arctic breeding shorebird: how do pairs divide their duties?
Source: Behav Ecol. 2013 Oct 29;25(1):152–64. doi: 10.1093/beheco/art098 (PMC3860833; doi:10.1093/beheco/art098)
Supplement: Supplementary Data [file supp_25_1_152__index.html]

Biparental incubation patterns in a high-Arctic breeding shorebird: how do pairs divide their duties? — Supplementary Data 

# Biparental incubation patterns in a high-Arctic breeding shorebird: how do pairs divide their duties?

## Supplementary Data

Data files

**Files in this Data Supplement:**

- Supplementary Data - Supplementary Data
- Supplementary Data - Supplementary Data
- Supplementary Data - Supplementary Data
- Supplementary Data - Supplementary Data
