# Supplementary material for: Biparental incubation patterns in a high-Arctic breeding shorebird: how do pairs divide their duties?
Source: Behav Ecol. 2013 Oct 29;25(1):152–64. doi: 10.1093/beheco/art098 (PMC3860833; doi:10.1093/beheco/art098)
Supplement: Supplementary Data [file supp_art098_El.Supplement_ACTOGRAMS.pdf]

ELECTRONIC SUPPLEMENT - Actograms

The following actograms depict nest-specific incubation patterns. Each of 48 nests is represented by one actogram that contains only data used in the models (i.e., some data were excluded prior to analyses – the procedure is described in Methods, sections *Statistical analyses – Timing,* and *Sample sizes*). First date indicates the estimated start of incubation (1st nest started June 1, last June 26). All figures depict 23 days, even if the incubation ended earlier. The actograms are ordered according to the start of incubation within the season.

A lighter color of incubation indicates 'assumed incubation'. This refers to cases where the temperature recordings failed and only one parent had an RFID tag (nest S304 and S403). A horizontal black-line crossing incubation bouts indicates bouts that was excluded from the incubation constancy and exchange gap models because of failed temperature recordings (nest S101, S304, S320 and S403) or a displaced temperature probe (nest S503).

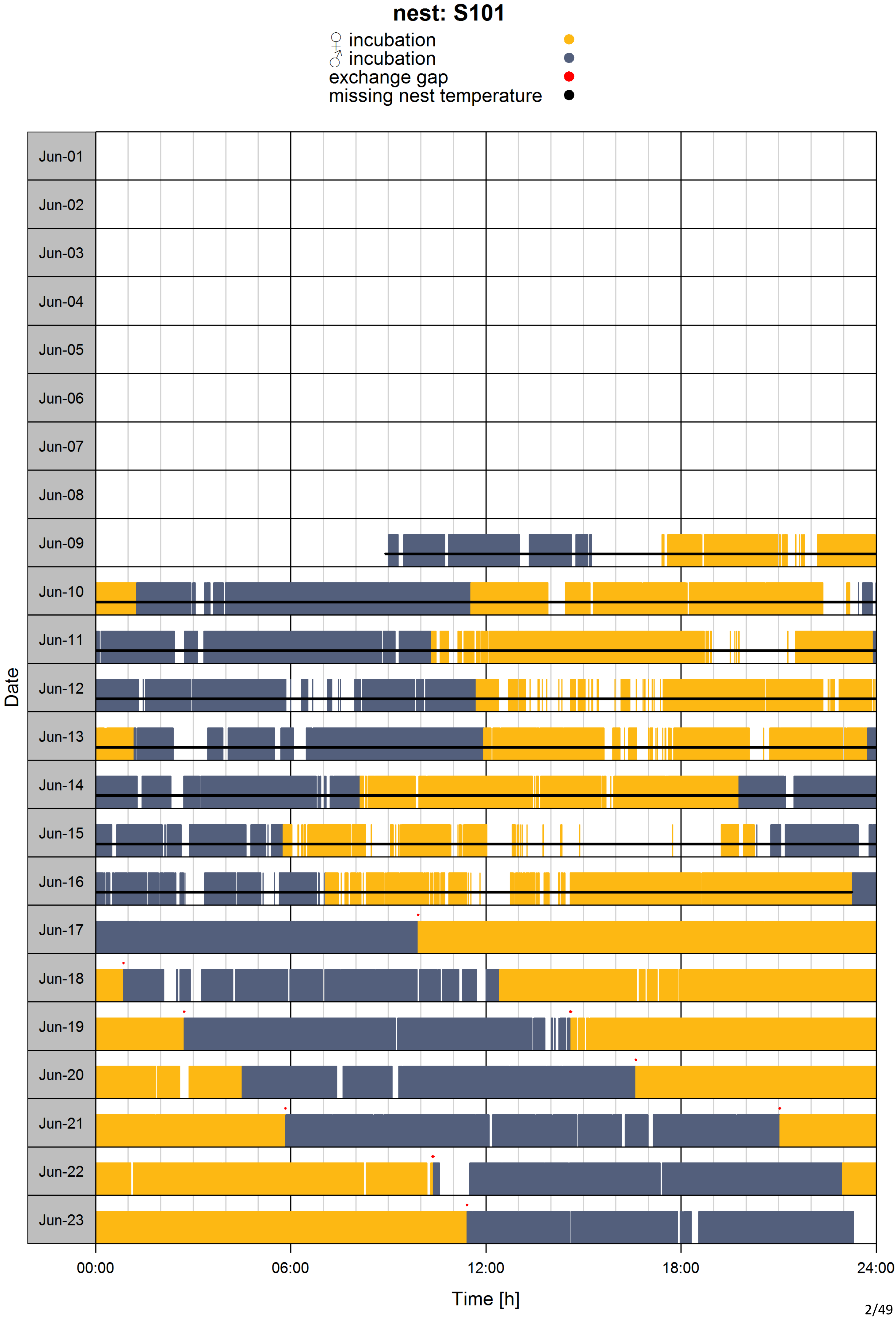

nest: S101
♀ incubation
♂ incubation
exchange gap
missing nest temperature
Date
Jun-01
Jun-02
Jun-03
Jun-04
Jun-05
Jun-06
Jun-07
Jun-08
Jun-09
Jun-10
Jun-11
Jun-12
Jun-13
Jun-14
Jun-15
Jun-16
Jun-17
Jun-18
Jun-19
Jun-20
Jun-21
Jun-22
Jun-23
00:00
06:00
12:00
18:00
24:00
Time [h]

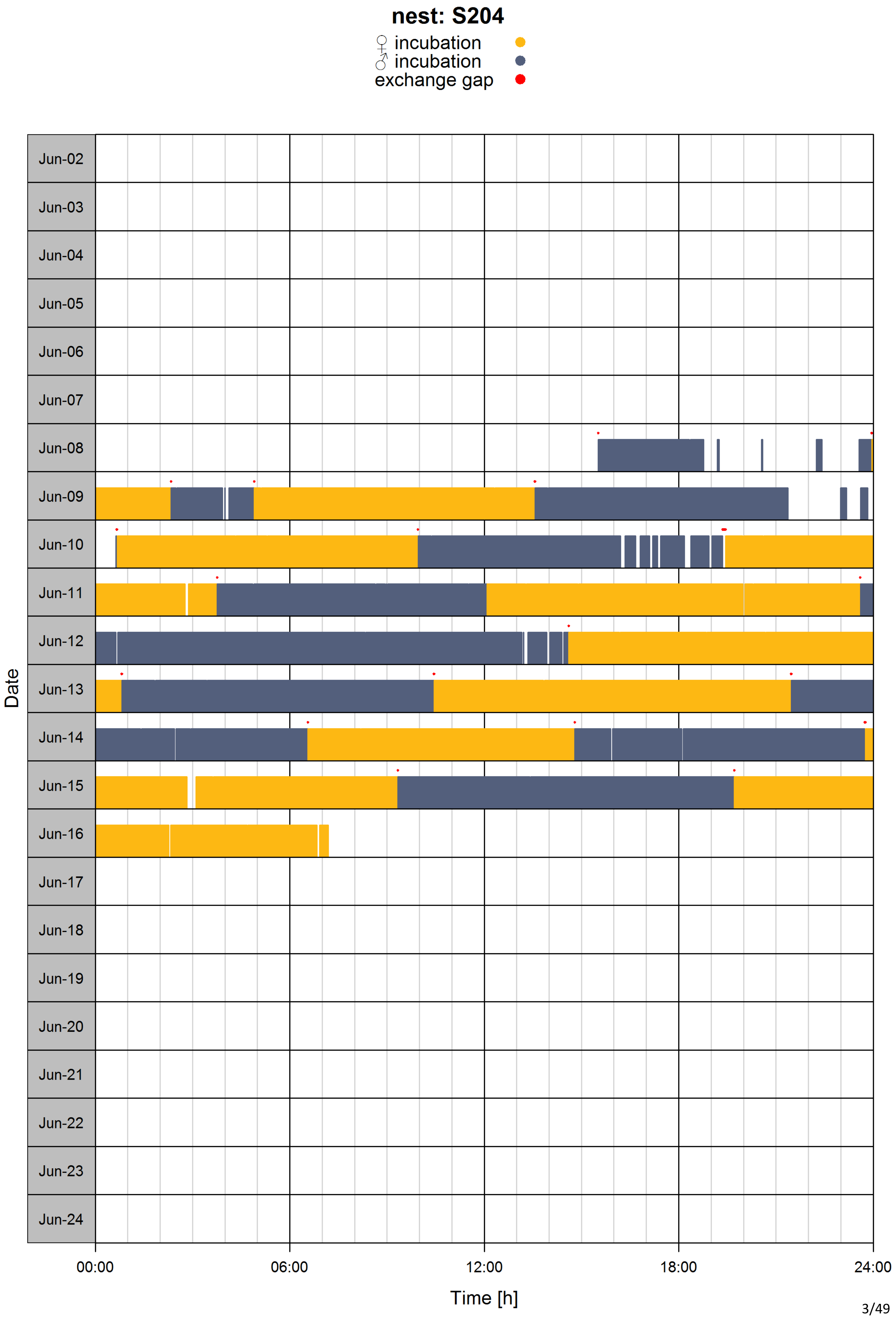
nest: S204
♀ incubation
♂ incubation
exchange gap
Date
Jun-02
Jun-03
Jun-04
Jun-05
Jun-06
Jun-07
Jun-08
Jun-09
Jun-10
Jun-11
Jun-12
Jun-13
Jun-14
Jun-15
Jun-16
Jun-17
Jun-18
Jun-19
Jun-20
Jun-21
Jun-22
Jun-23
Jun-24
00:00
06:00
12:00
18:00
24:00
Time [h]

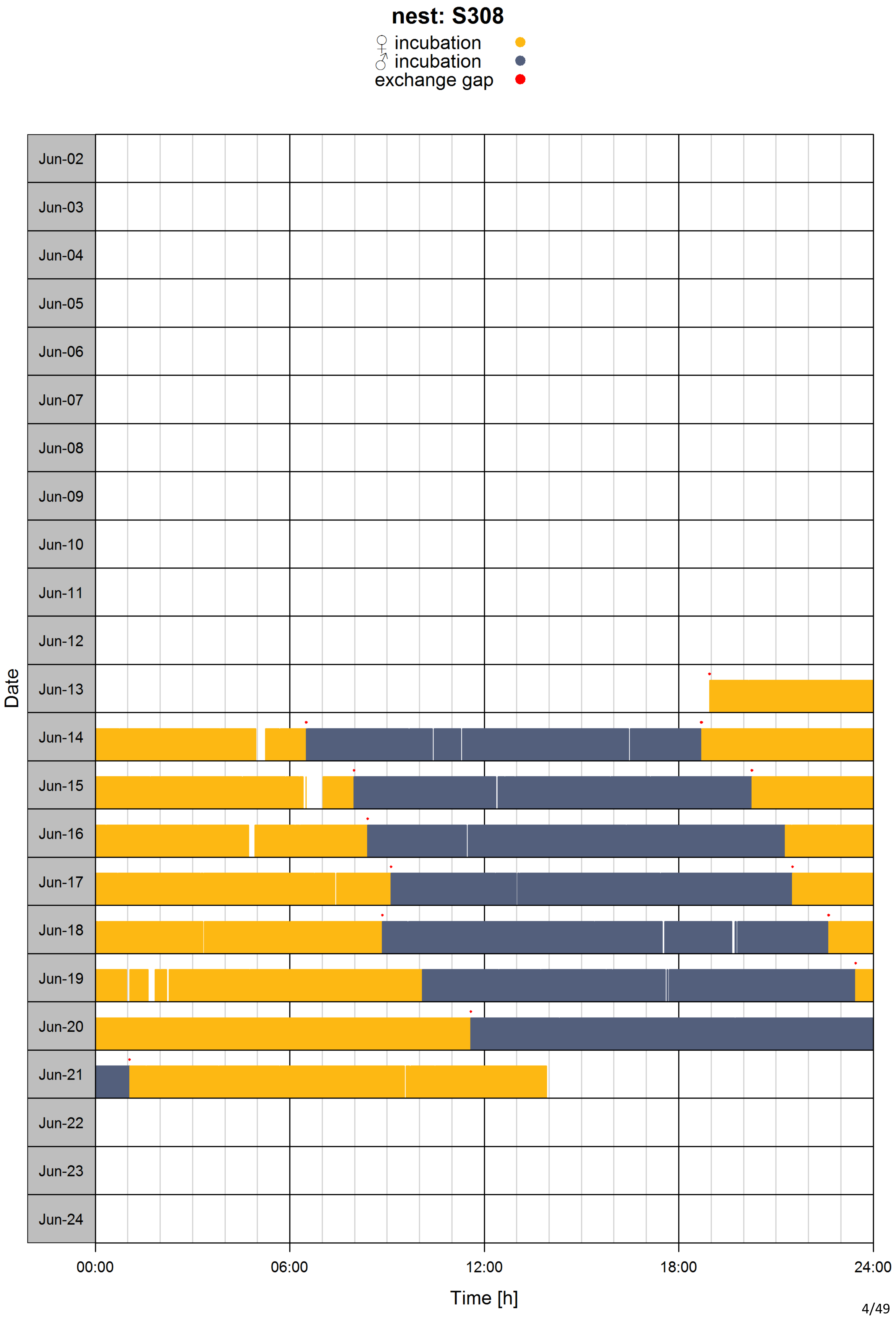
nest: S308
♀ incubation
♂ incubation
exchange gap
Date
Jun-02
Jun-03
Jun-04
Jun-05
Jun-06
Jun-07
Jun-08
Jun-09
Jun-10
Jun-11
Jun-12
Jun-13
Jun-14
Jun-15
Jun-16
Jun-17
Jun-18
Jun-19
Jun-20
Jun-21
Jun-22
Jun-23
Jun-24
00:00
06:00
12:00
18:00
24:00
Time [h]

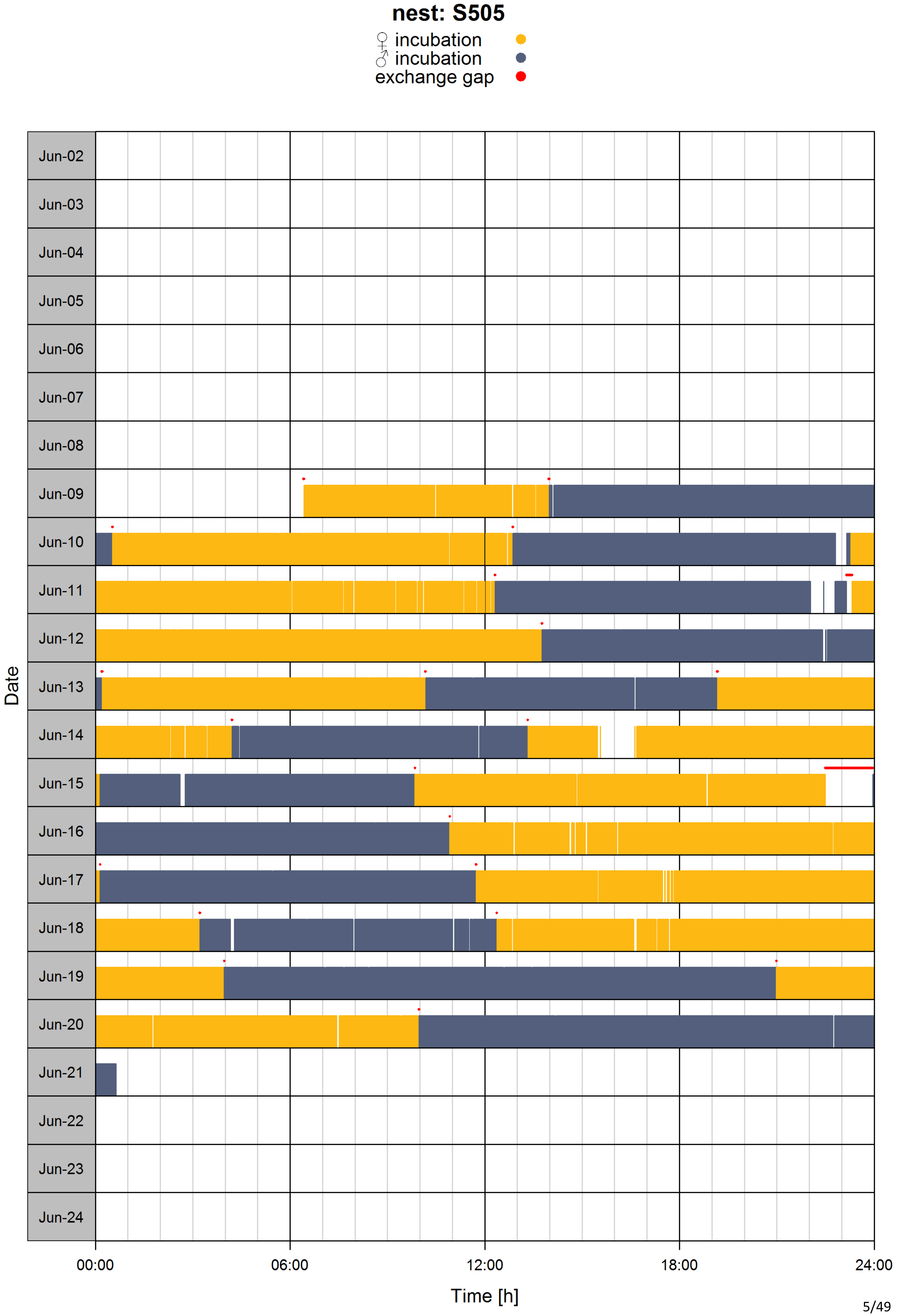

nest: S505
♀ incubation
♂ incubation
exchange gap
Date
Jun-02
Jun-03
Jun-04
Jun-05
Jun-06
Jun-07
Jun-08
Jun-09
Jun-10
Jun-11
Jun-12
Jun-13
Jun-14
Jun-15
Jun-16
Jun-17
Jun-18
Jun-19
Jun-20
Jun-21
Jun-22
Jun-23
Jun-24
00:00
06:00
12:00
18:00
24:00
Time [h]

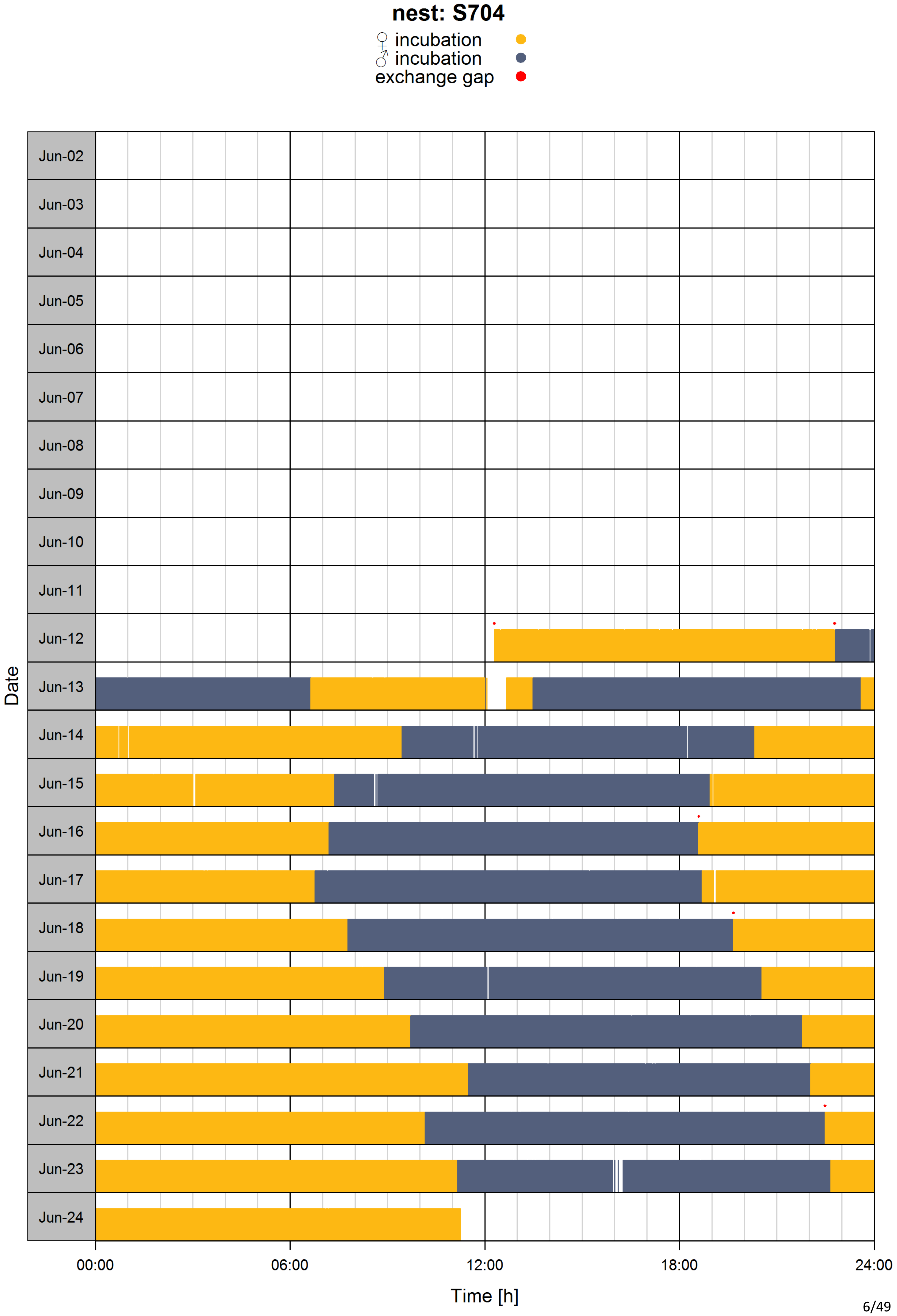
nest: S704
♀ incubation
♂ incubation
exchange gap
Date
Jun-02
Jun-03
Jun-04
Jun-05
Jun-06
Jun-07
Jun-08
Jun-09
Jun-10
Jun-11
Jun-12
Jun-13
Jun-14
Jun-15
Jun-16
Jun-17
Jun-18
Jun-19
Jun-20
Jun-21
Jun-22
Jun-23
Jun-24
00:00
06:00
12:00
18:00
24:00
Time [h]

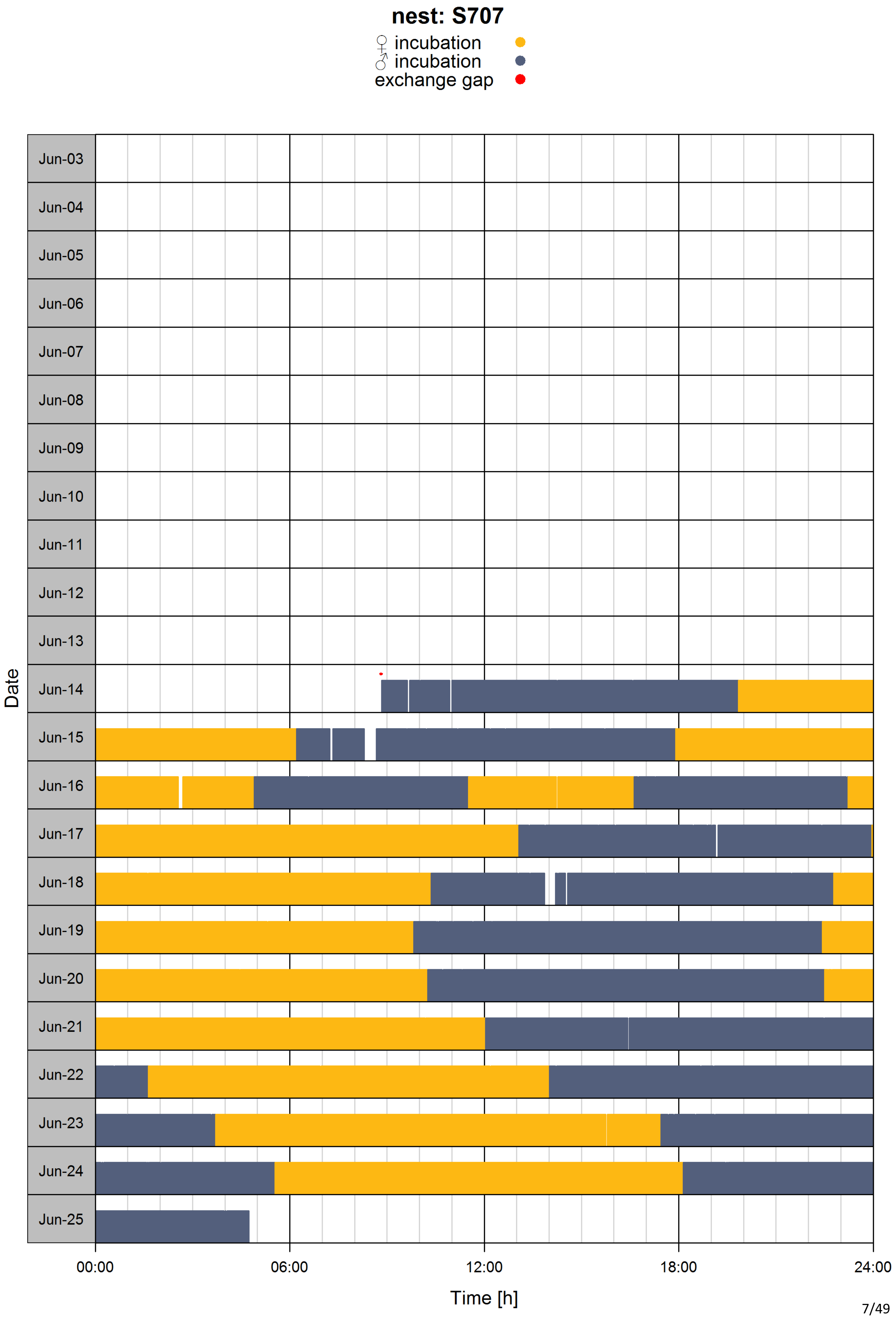
nest: S707
♀ incubation
♂ incubation
exchange gap
Date
Jun-03
Jun-04
Jun-05
Jun-06
Jun-07
Jun-08
Jun-09
Jun-10
Jun-11
Jun-12
Jun-13
Jun-14
Jun-15
Jun-16
Jun-17
Jun-18
Jun-19
Jun-20
Jun-21
Jun-22
Jun-23
Jun-24
Jun-25
00:00
06:00
12:00
18:00
24:00
Time [h]

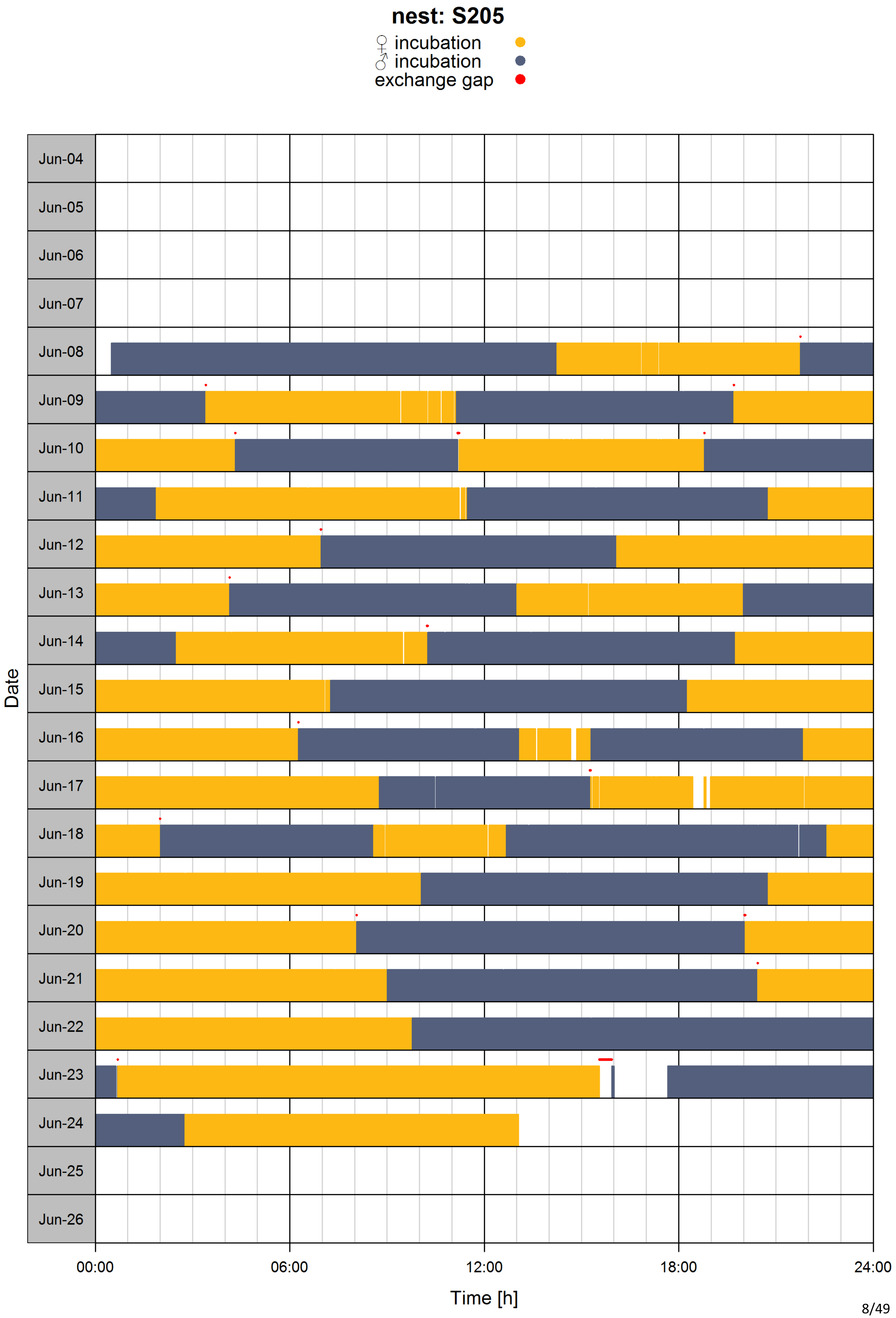

nest: S205
♀ incubation
♂ incubation
exchange gap
Date
Jun-04
Jun-05
Jun-06
Jun-07
Jun-08
Jun-09
Jun-10
Jun-11
Jun-12
Jun-13
Jun-14
Jun-15
Jun-16
Jun-17
Jun-18
Jun-19
Jun-20
Jun-21
Jun-22
Jun-23
Jun-24
Jun-25
Jun-26
00:00
06:00
12:00
18:00
24:00
Time [h]

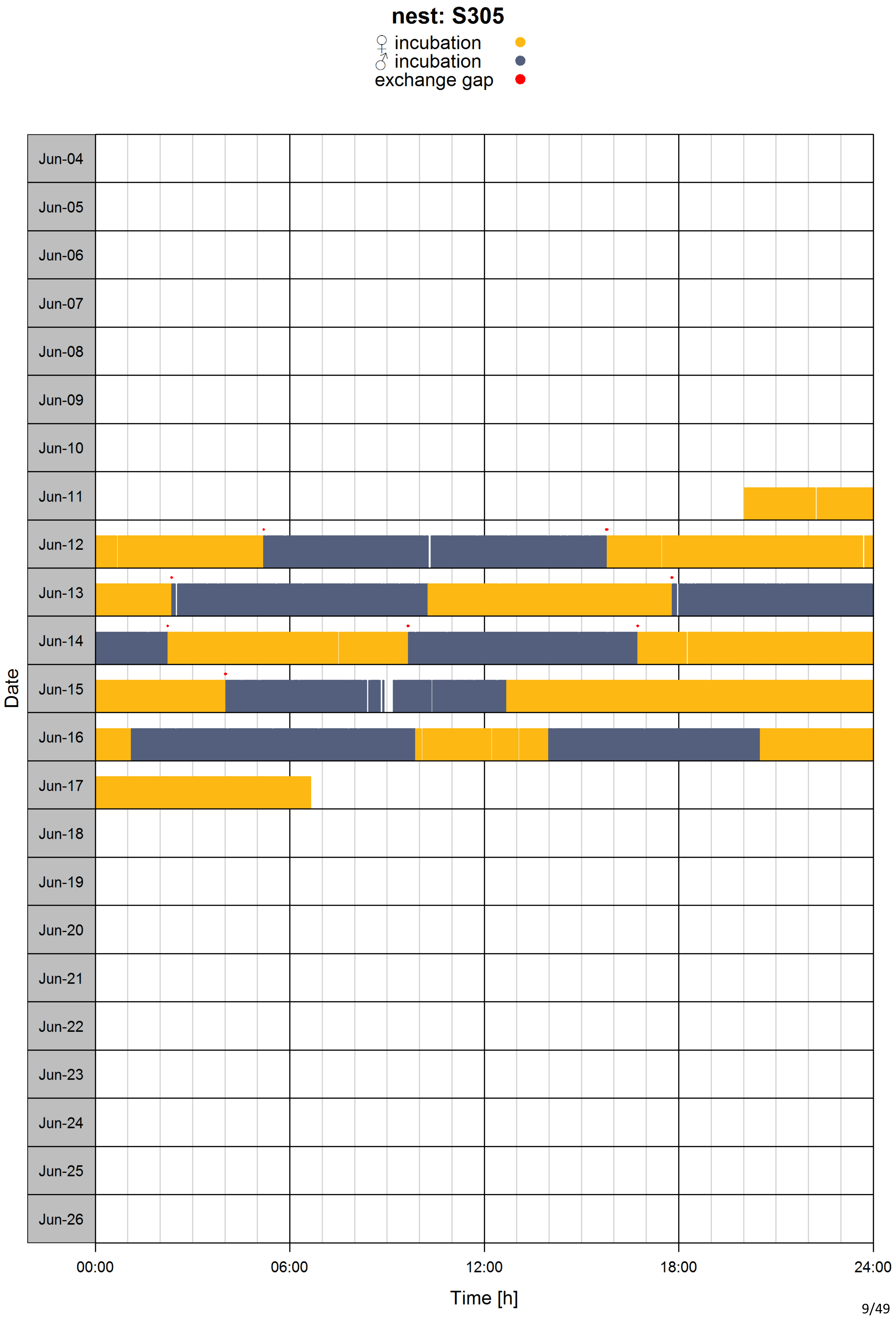

nest: S305
♀ incubation
♂ incubation
exchange gap
Date
Jun-04
Jun-05
Jun-06
Jun-07
Jun-08
Jun-09
Jun-10
Jun-11
Jun-12
Jun-13
Jun-14
Jun-15
Jun-16
Jun-17
Jun-18
Jun-19
Jun-20
Jun-21
Jun-22
Jun-23
Jun-24
Jun-25
Jun-26
00:00
06:00
12:00
18:00
24:00
Time [h]

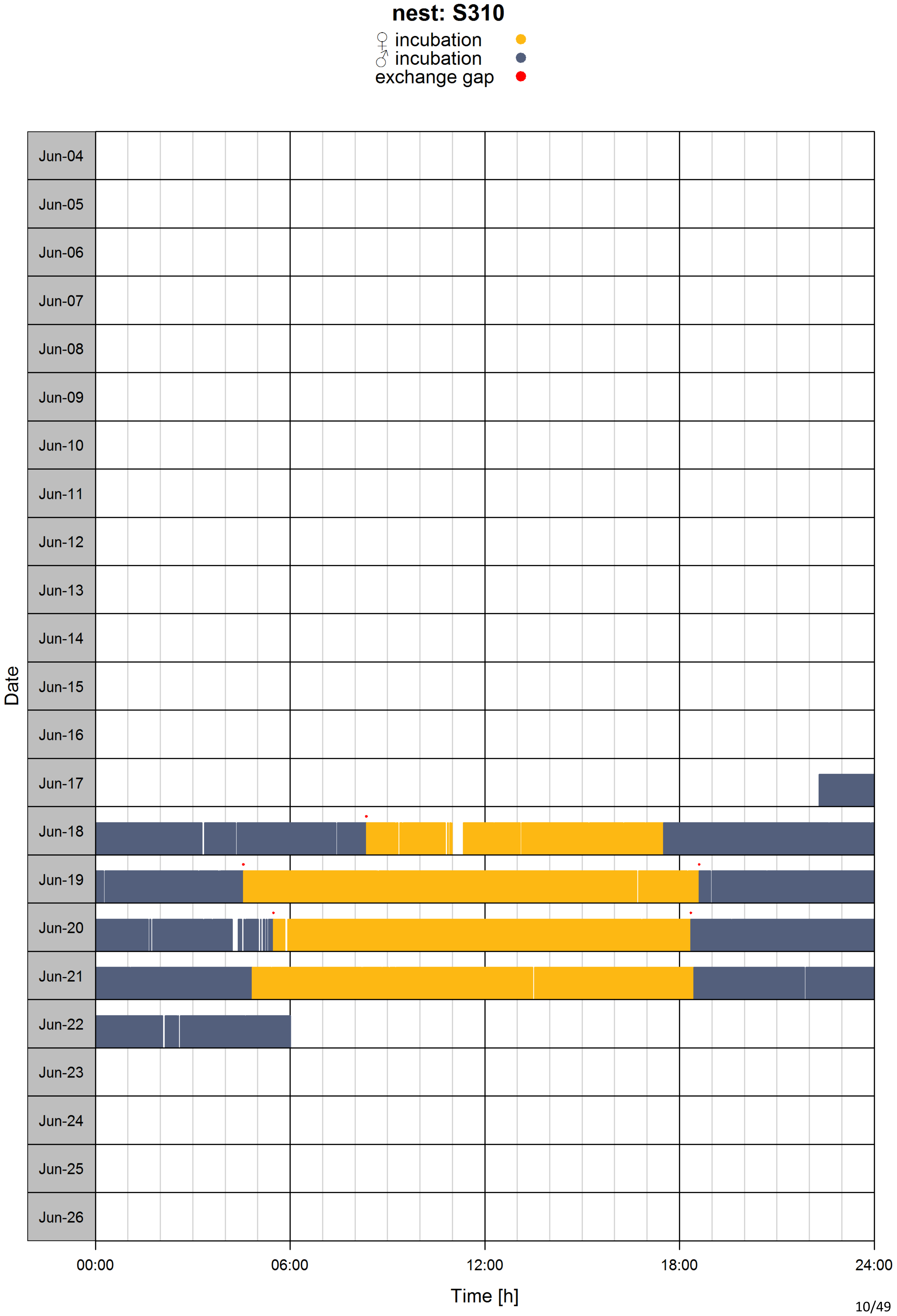

nest: S310
♀ incubation
♂ incubation
exchange gap
Date
Jun-04
Jun-05
Jun-06
Jun-07
Jun-08
Jun-09
Jun-10
Jun-11
Jun-12
Jun-13
Jun-14
Jun-15
Jun-16
Jun-17
Jun-18
Jun-19
Jun-20
Jun-21
Jun-22
Jun-23
Jun-24
Jun-25
Jun-26
00:00
06:00
12:00
18:00
24:00
Time [h]

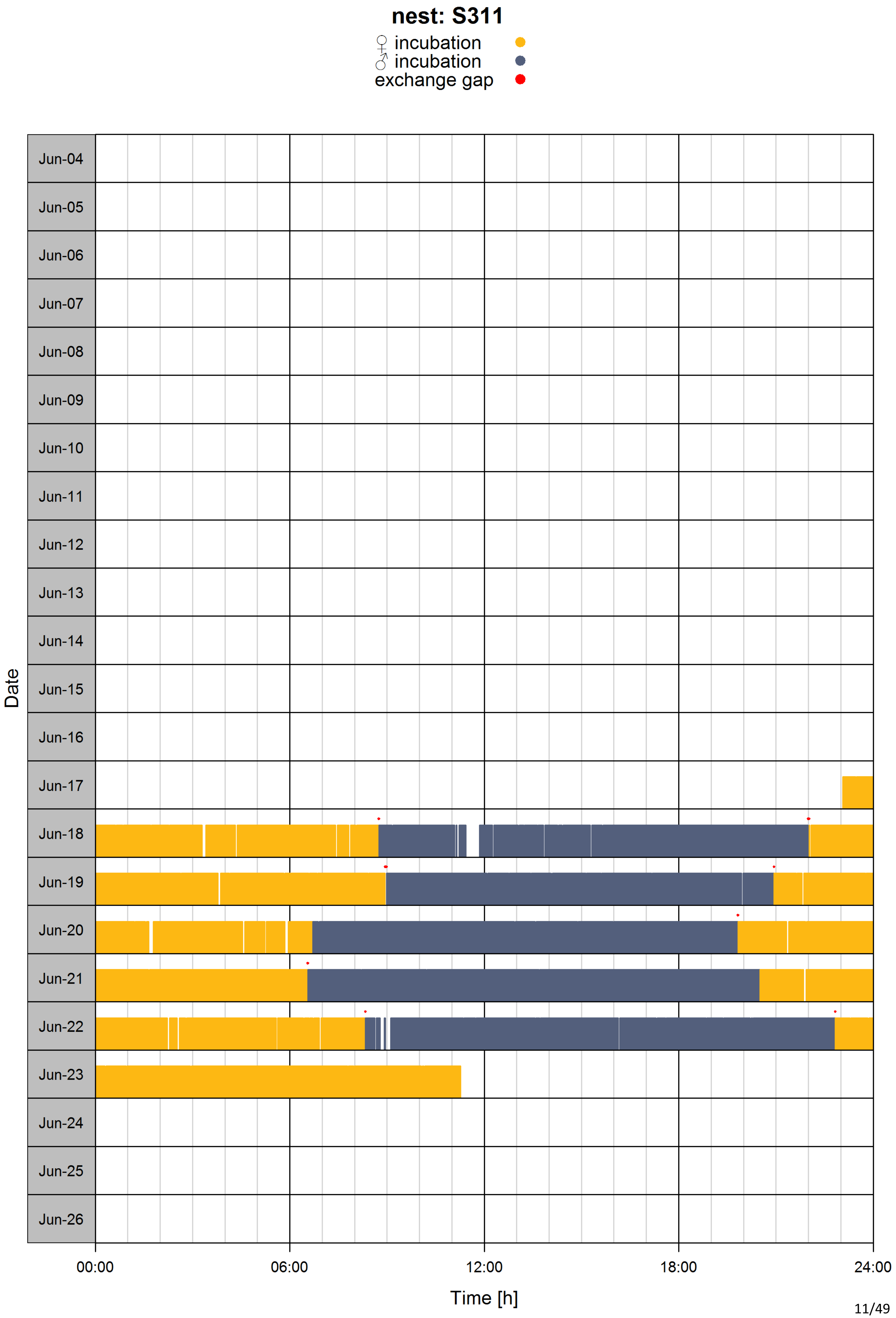
nest: S311
♀ incubation
♂ incubation
exchange gap
Date
Jun-04
Jun-05
Jun-06
Jun-07
Jun-08
Jun-09
Jun-10
Jun-11
Jun-12
Jun-13
Jun-14
Jun-15
Jun-16
Jun-17
Jun-18
Jun-19
Jun-20
Jun-21
Jun-22
Jun-23
Jun-24
Jun-25
Jun-26
00:00
06:00
12:00
18:00
24:00
Time [h]

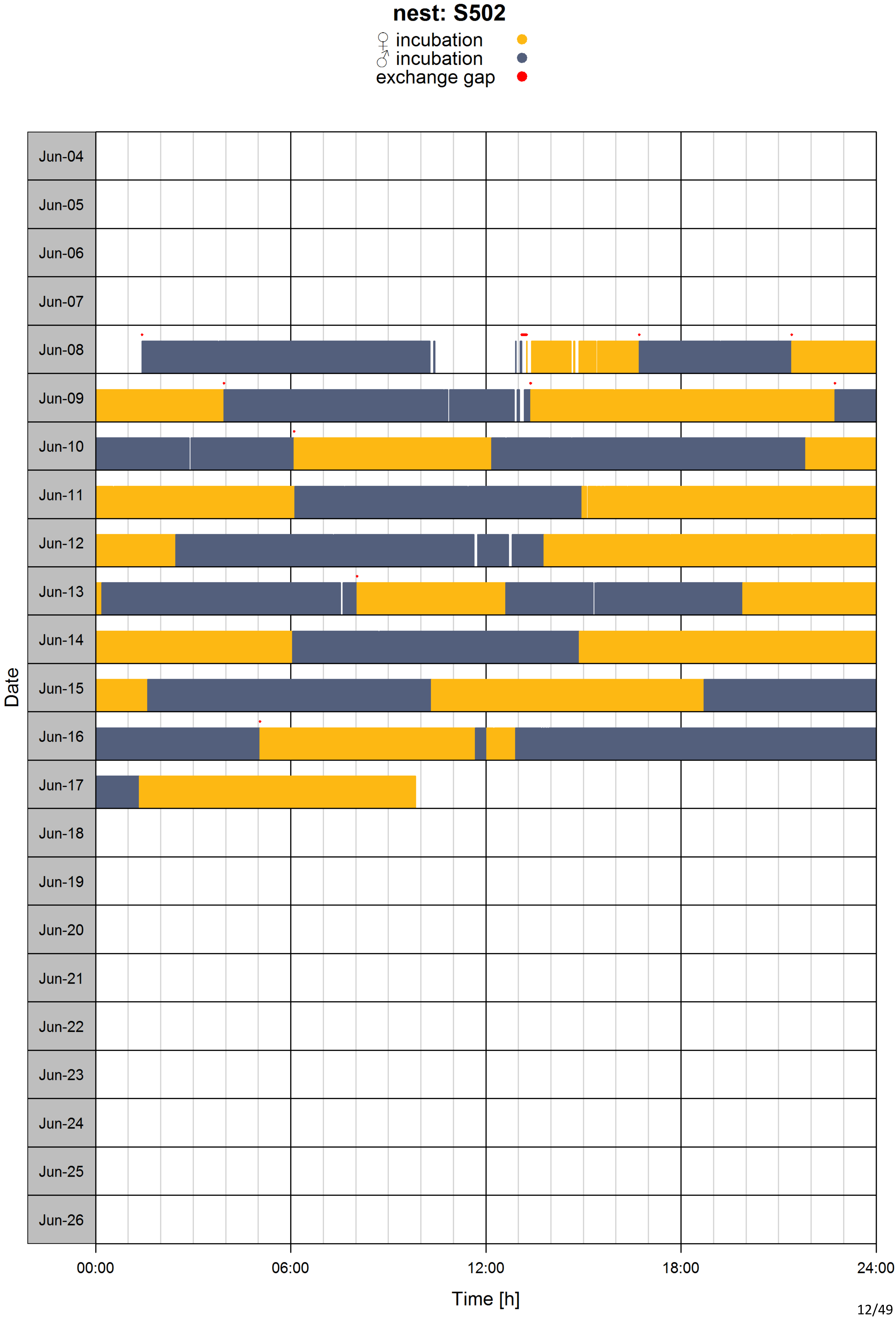
nest: S502
♀ incubation
♂ incubation
exchange gap
Jun-04
Jun-05
Jun-06
Jun-07
Jun-08
Jun-09
Jun-10
Jun-11
Jun-12
Jun-13
Jun-14
Jun-15
Jun-16
Jun-17
Jun-18
Jun-19
Jun-20
Jun-21
Jun-22
Jun-23
Jun-24
Jun-25
Jun-26
Date
00:00
06:00
12:00
18:00
24:00
Time [h]

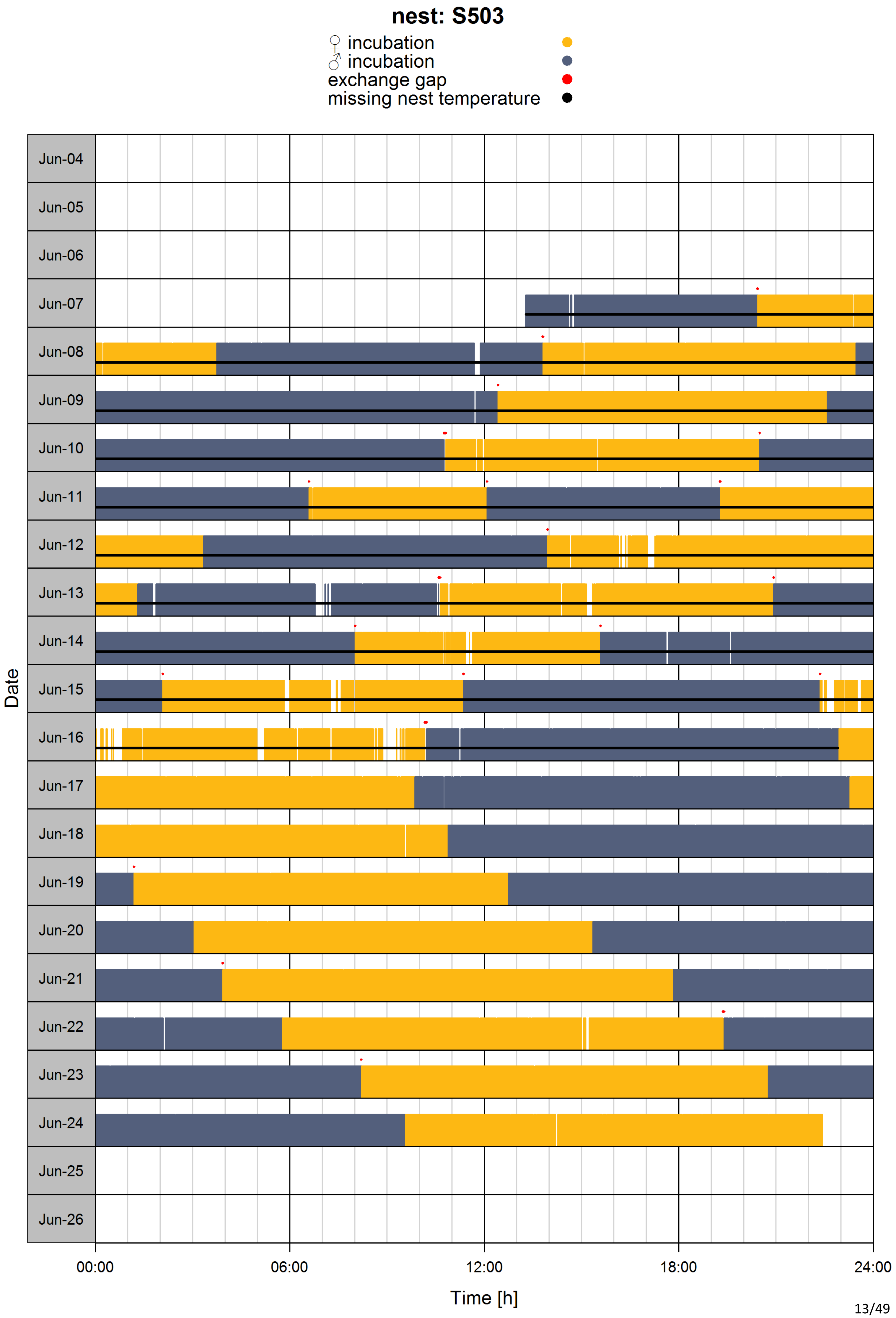
nest: S503
♀ incubation
♂ incubation
exchange gap
missing nest temperature
Jun-04
Jun-05
Jun-06
Jun-07
Jun-08
Jun-09
Jun-10
Jun-11
Jun-12
Jun-13
Jun-14
Jun-15
Jun-16
Jun-17
Jun-18
Jun-19
Jun-20
Jun-21
Jun-22
Jun-23
Jun-24
Jun-25
Jun-26
Date
00:00
06:00
12:00
18:00
24:00
Time [h]

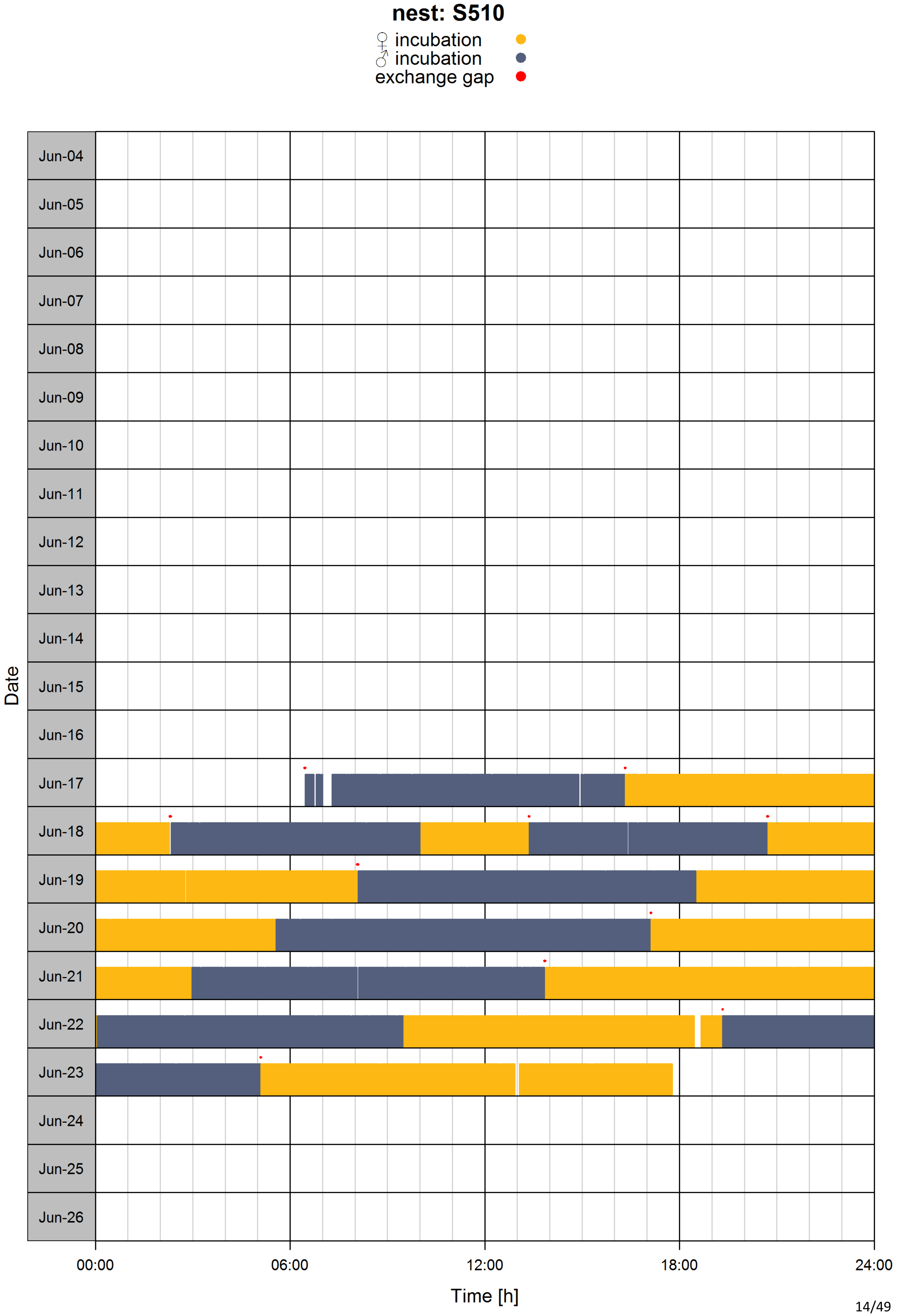
nest: S510
♀ incubation
♂ incubation
exchange gap
Date
Jun-04
Jun-05
Jun-06
Jun-07
Jun-08
Jun-09
Jun-10
Jun-11
Jun-12
Jun-13
Jun-14
Jun-15
Jun-16
Jun-17
Jun-18
Jun-19
Jun-20
Jun-21
Jun-22
Jun-23
Jun-24
Jun-25
Jun-26
00:00
06:00
12:00
18:00
24:00
Time [h]

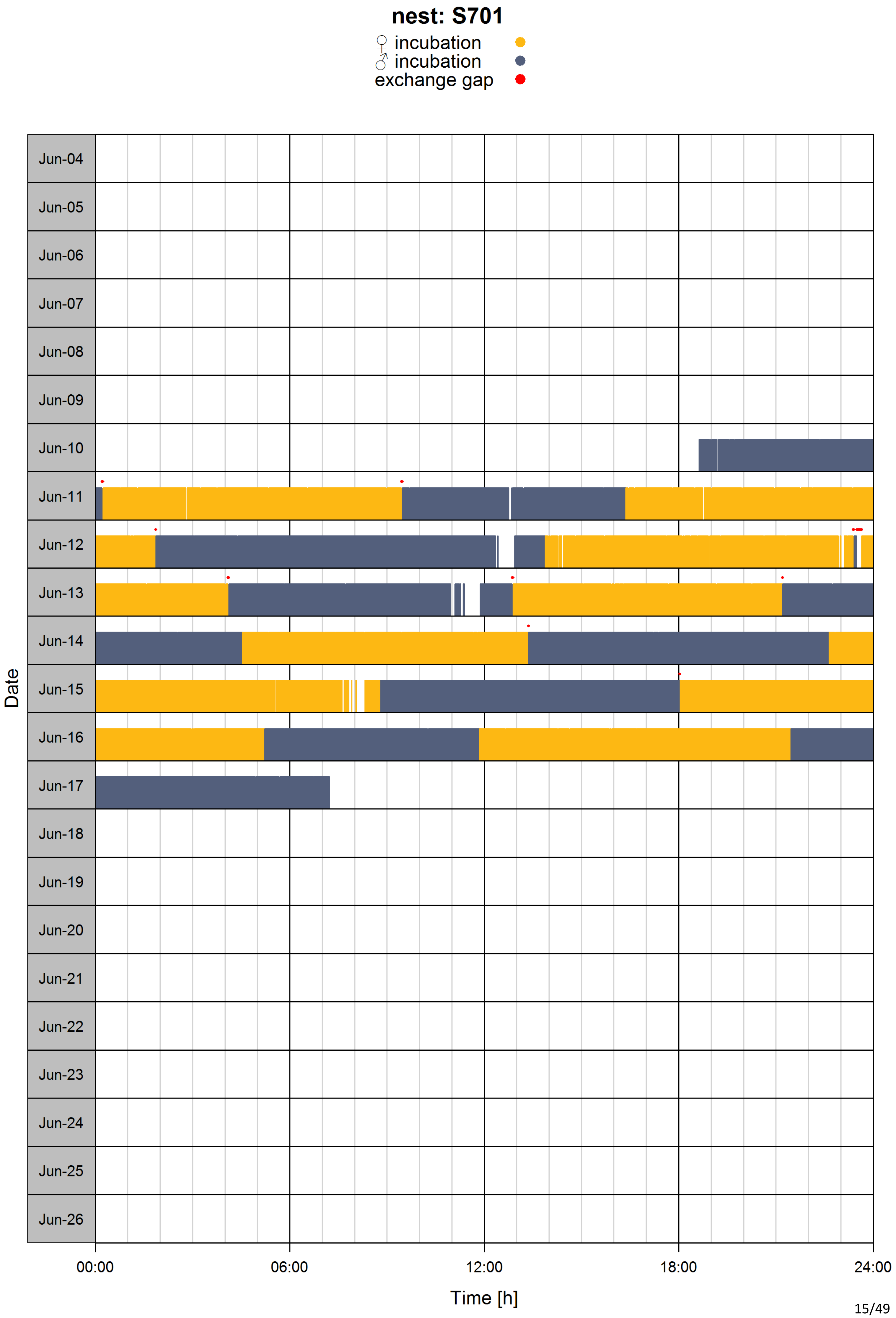
nest: S701
♀ incubation
♂ incubation
exchange gap
Date
Jun-04
Jun-05
Jun-06
Jun-07
Jun-08
Jun-09
Jun-10
Jun-11
Jun-12
Jun-13
Jun-14
Jun-15
Jun-16
Jun-17
Jun-18
Jun-19
Jun-20
Jun-21
Jun-22
Jun-23
Jun-24
Jun-25
Jun-26
00:00
06:00
12:00
18:00
24:00
Time [h]

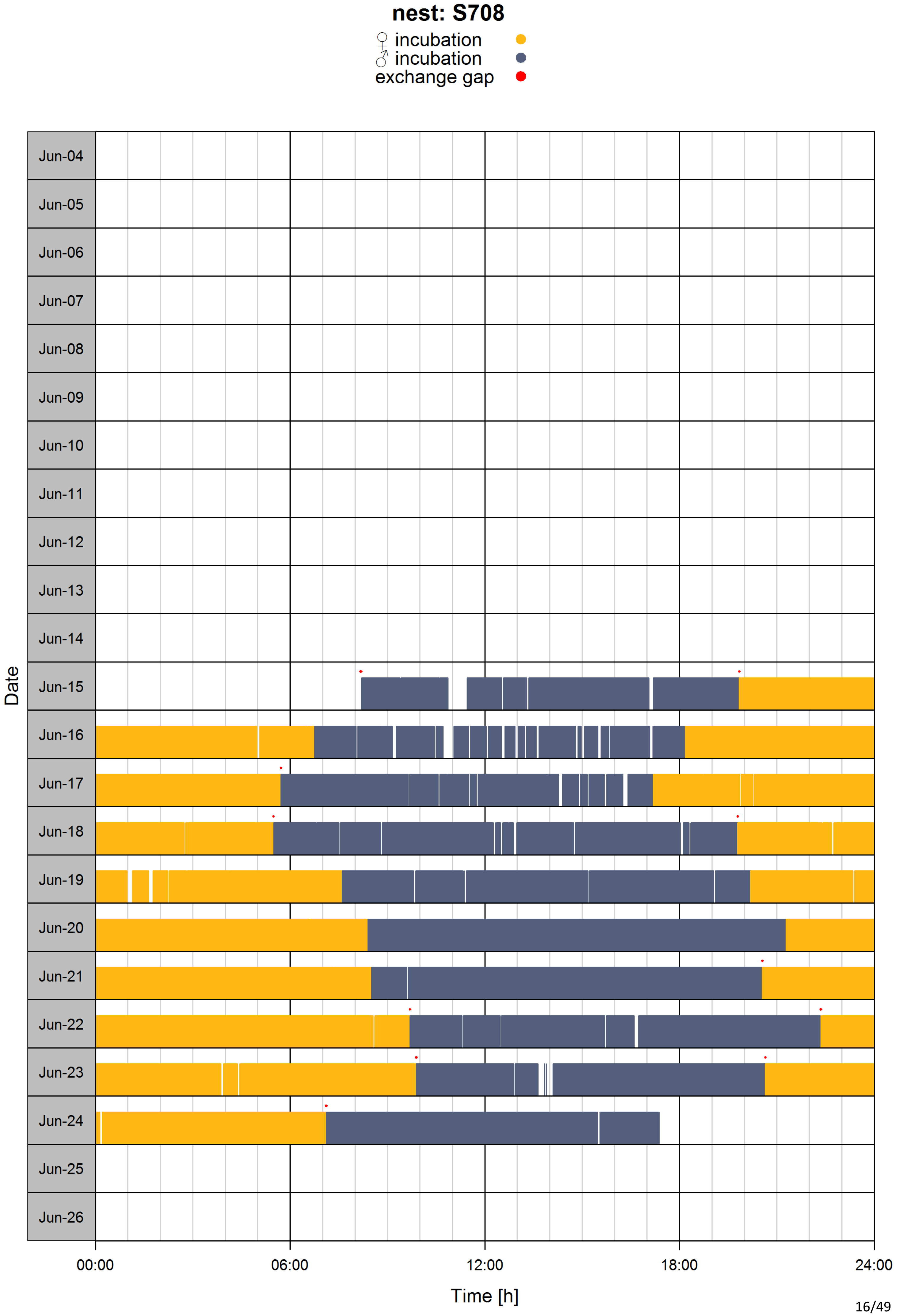
nest: S708
♀ incubation
♂ incubation
exchange gap
Date
Jun-04
Jun-05
Jun-06
Jun-07
Jun-08
Jun-09
Jun-10
Jun-11
Jun-12
Jun-13
Jun-14
Jun-15
Jun-16
Jun-17
Jun-18
Jun-19
Jun-20
Jun-21
Jun-22
Jun-23
Jun-24
Jun-25
Jun-26
00:00
06:00
12:00
18:00
24:00
Time [h]

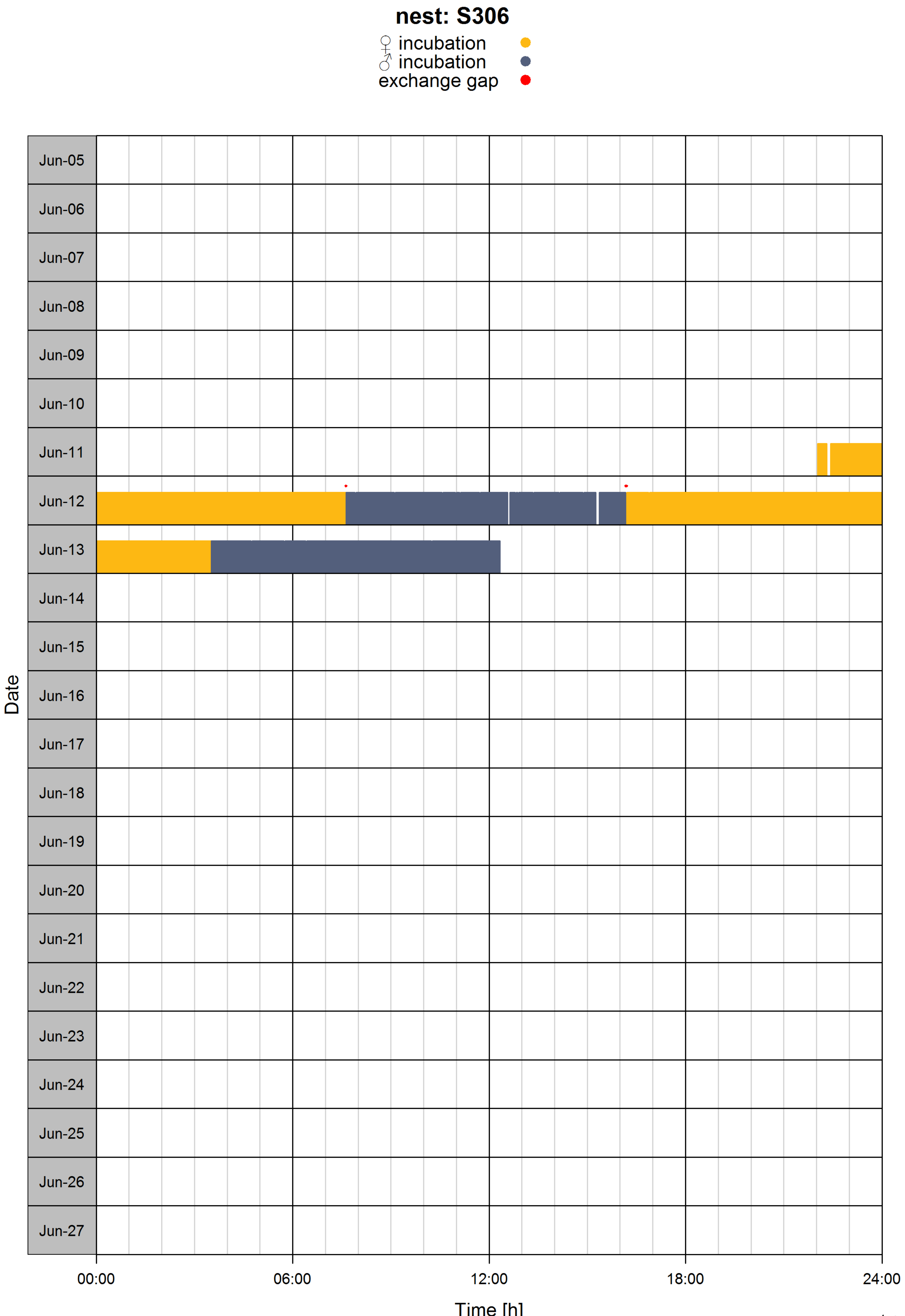

nest: S306
♀ incubation
♂ incubation
exchange gap
Date
Jun-05
Jun-06
Jun-07
Jun-08
Jun-09
Jun-10
Jun-11
Jun-12
Jun-13
Jun-14
Jun-15
Jun-16
Jun-17
Jun-18
Jun-19
Jun-20
Jun-21
Jun-22
Jun-23
Jun-24
Jun-25
Jun-26
Jun-27
00:00
06:00
12:00
18:00
24:00
Time [h]

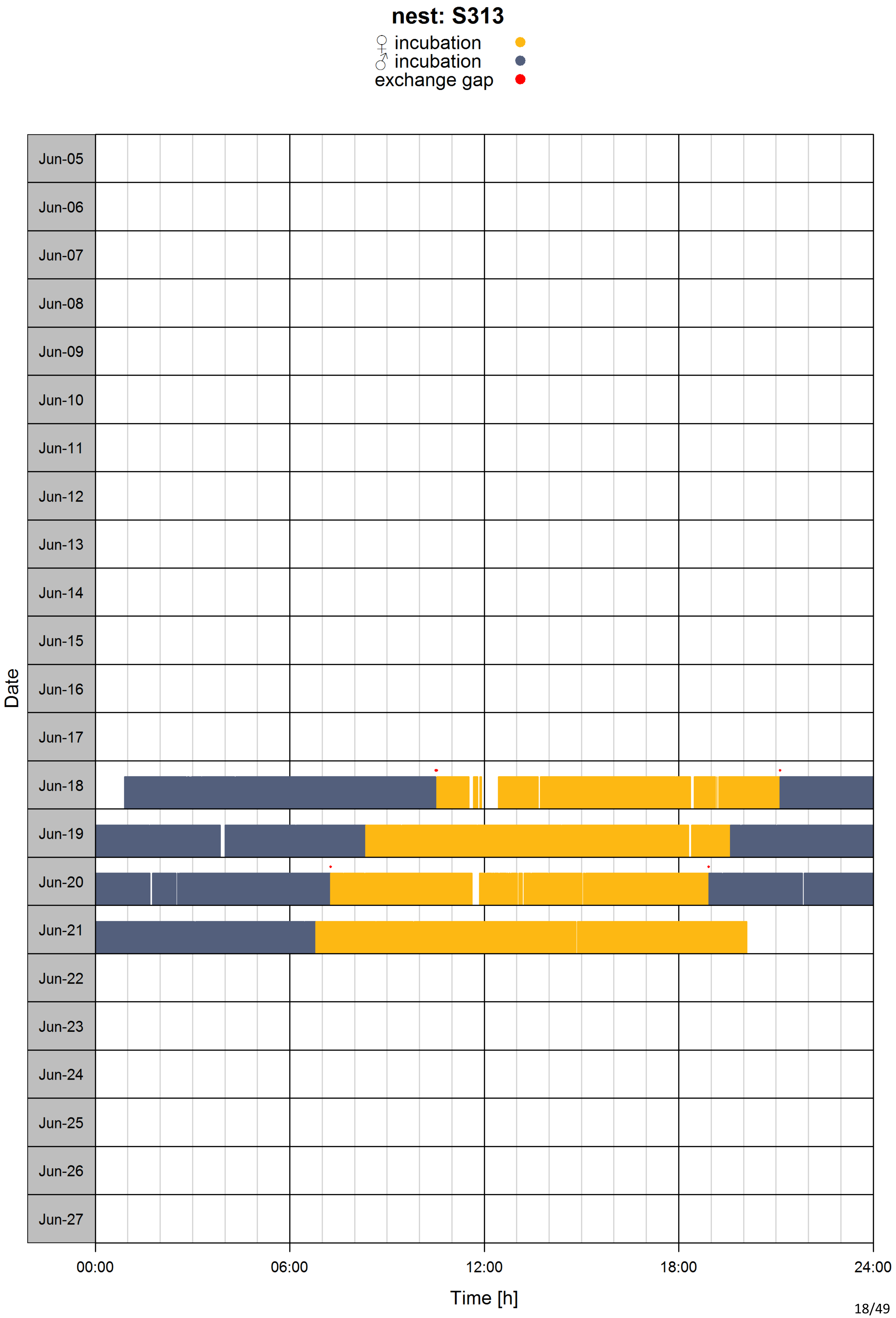
nest: S313
♀ incubation
♂ incubation
exchange gap
Date
Jun-05
Jun-06
Jun-07
Jun-08
Jun-09
Jun-10
Jun-11
Jun-12
Jun-13
Jun-14
Jun-15
Jun-16
Jun-17
Jun-18
Jun-19
Jun-20
Jun-21
Jun-22
Jun-23
Jun-24
Jun-25
Jun-26
Jun-27
00:00
06:00
12:00
18:00
24:00
Time [h]

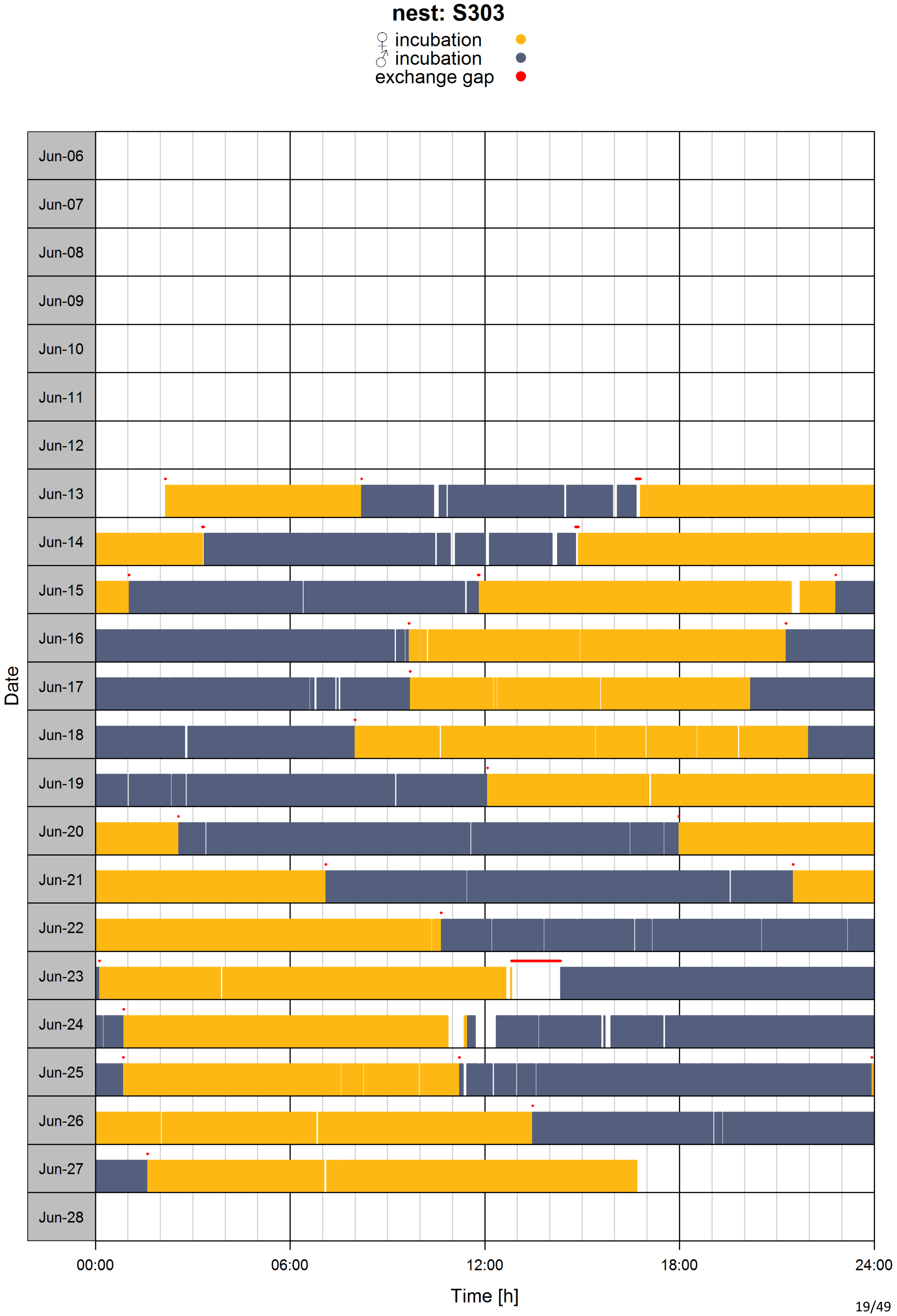

nest: S303
♀ incubation
♂ incubation
exchange gap
Date
Jun-06
Jun-07
Jun-08
Jun-09
Jun-10
Jun-11
Jun-12
Jun-13
Jun-14
Jun-15
Jun-16
Jun-17
Jun-18
Jun-19
Jun-20
Jun-21
Jun-22
Jun-23
Jun-24
Jun-25
Jun-26
Jun-27
Jun-28
00:00
06:00
12:00
18:00
24:00
Time [h]

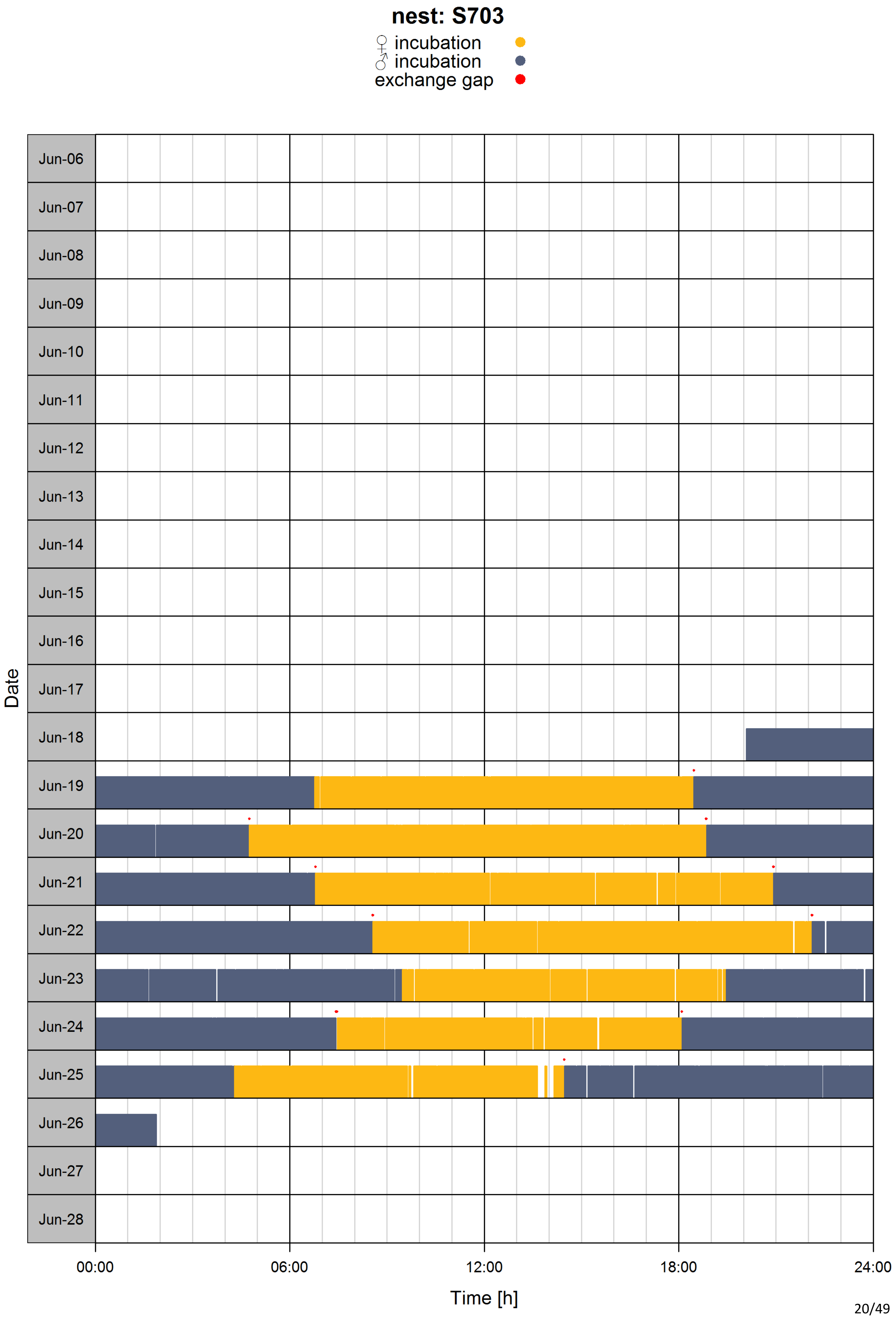
nest: S703
♀ incubation
♂ incubation
exchange gap
Date
Jun-06
Jun-07
Jun-08
Jun-09
Jun-10
Jun-11
Jun-12
Jun-13
Jun-14
Jun-15
Jun-16
Jun-17
Jun-18
Jun-19
Jun-20
Jun-21
Jun-22
Jun-23
Jun-24
Jun-25
Jun-26
Jun-27
Jun-28
00:00
06:00
12:00
18:00
24:00
Time [h]

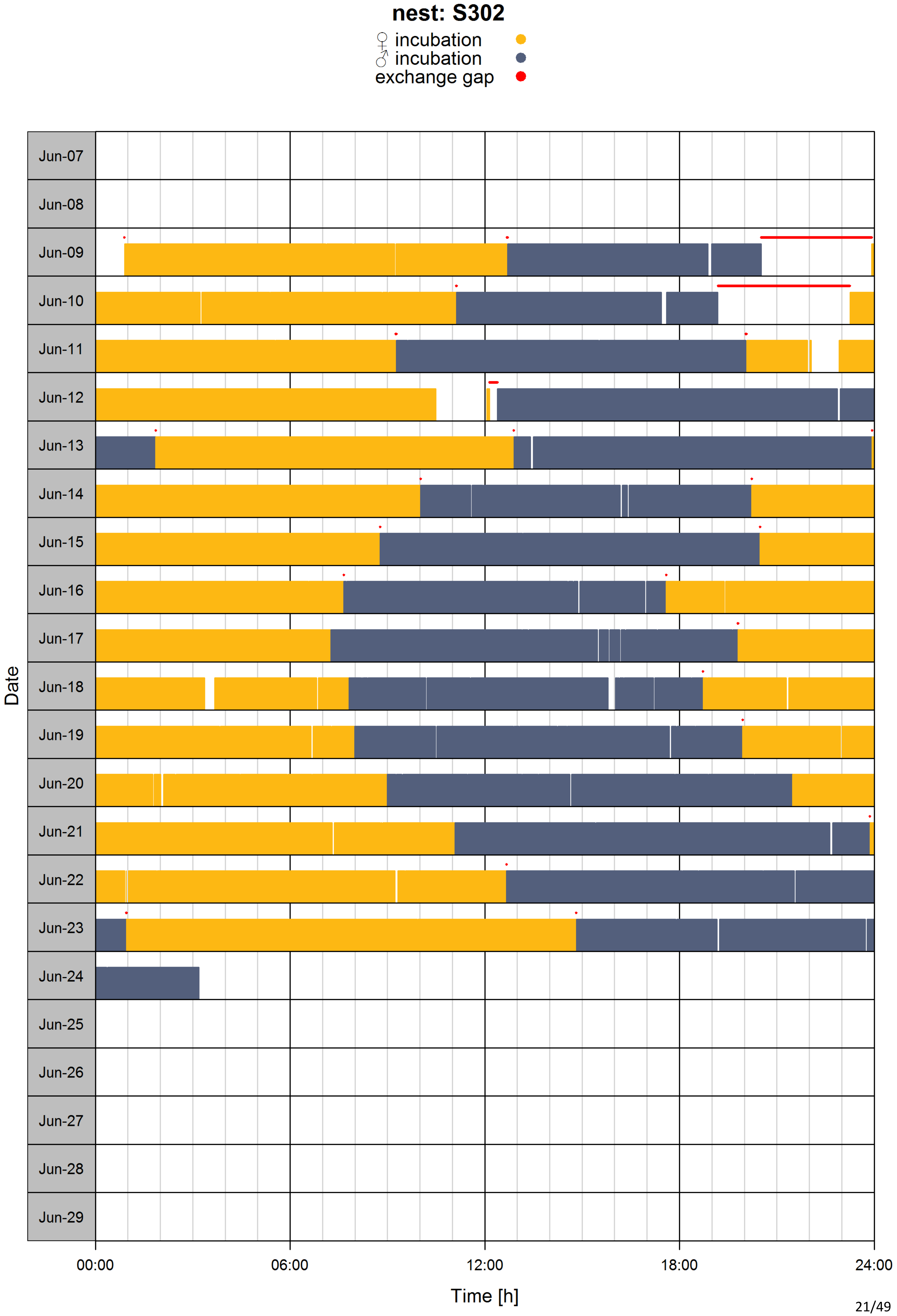
nest: S302
♀ incubation
♂ incubation
exchange gap
Jun-07
Jun-08
Jun-09
Jun-10
Jun-11
Jun-12
Jun-13
Jun-14
Jun-15
Jun-16
Jun-17
Jun-18
Jun-19
Jun-20
Jun-21
Jun-22
Jun-23
Jun-24
Jun-25
Jun-26
Jun-27
Jun-28
Jun-29
Date
00:00
06:00
12:00
18:00
24:00
Time [h]

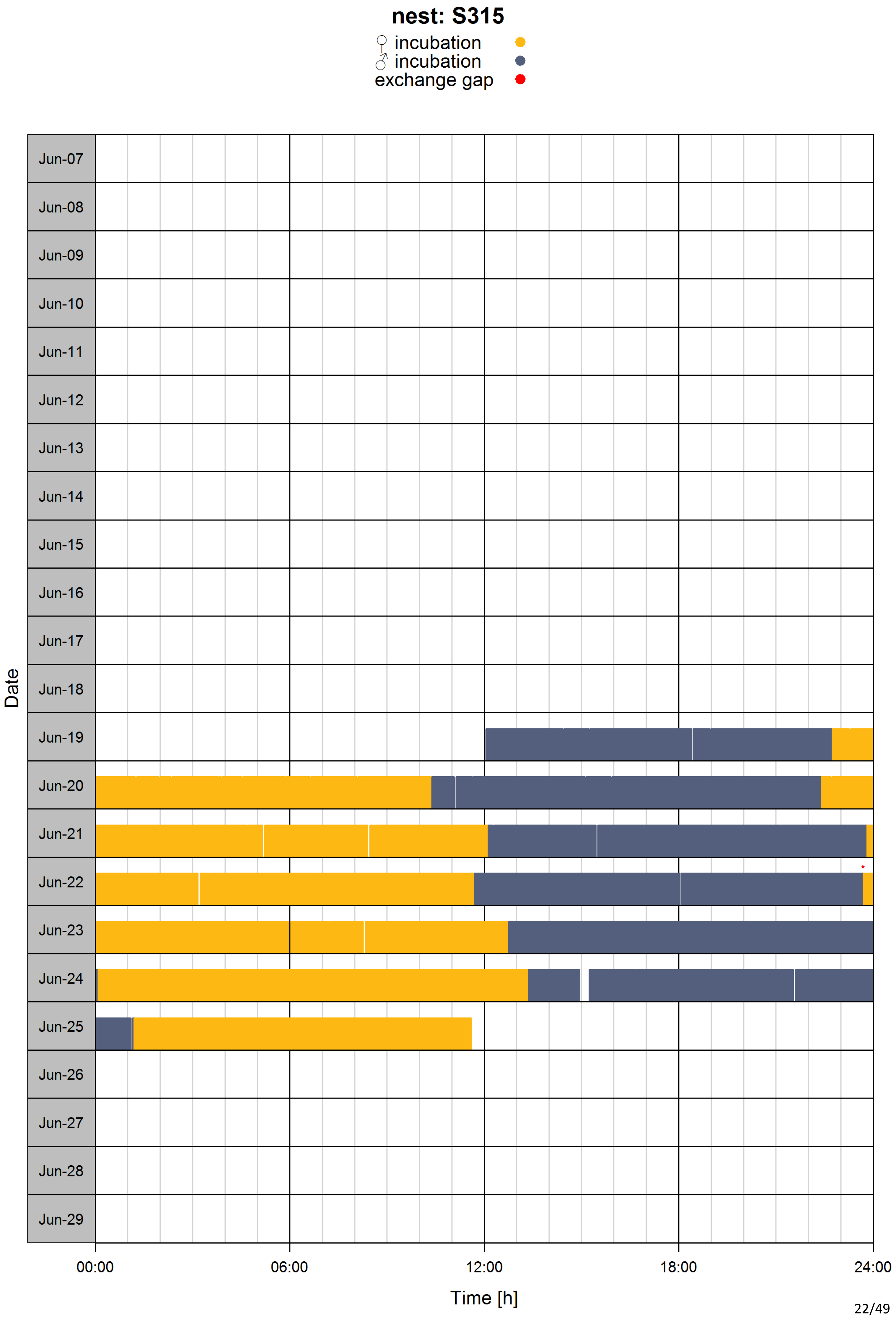
nest: S315
♀ incubation
♂ incubation
exchange gap
Date
Jun-07
Jun-08
Jun-09
Jun-10
Jun-11
Jun-12
Jun-13
Jun-14
Jun-15
Jun-16
Jun-17
Jun-18
Jun-19
Jun-20
Jun-21
Jun-22
Jun-23
Jun-24
Jun-25
Jun-26
Jun-27
Jun-28
Jun-29
00:00
06:00
12:00
18:00
24:00
Time [h]

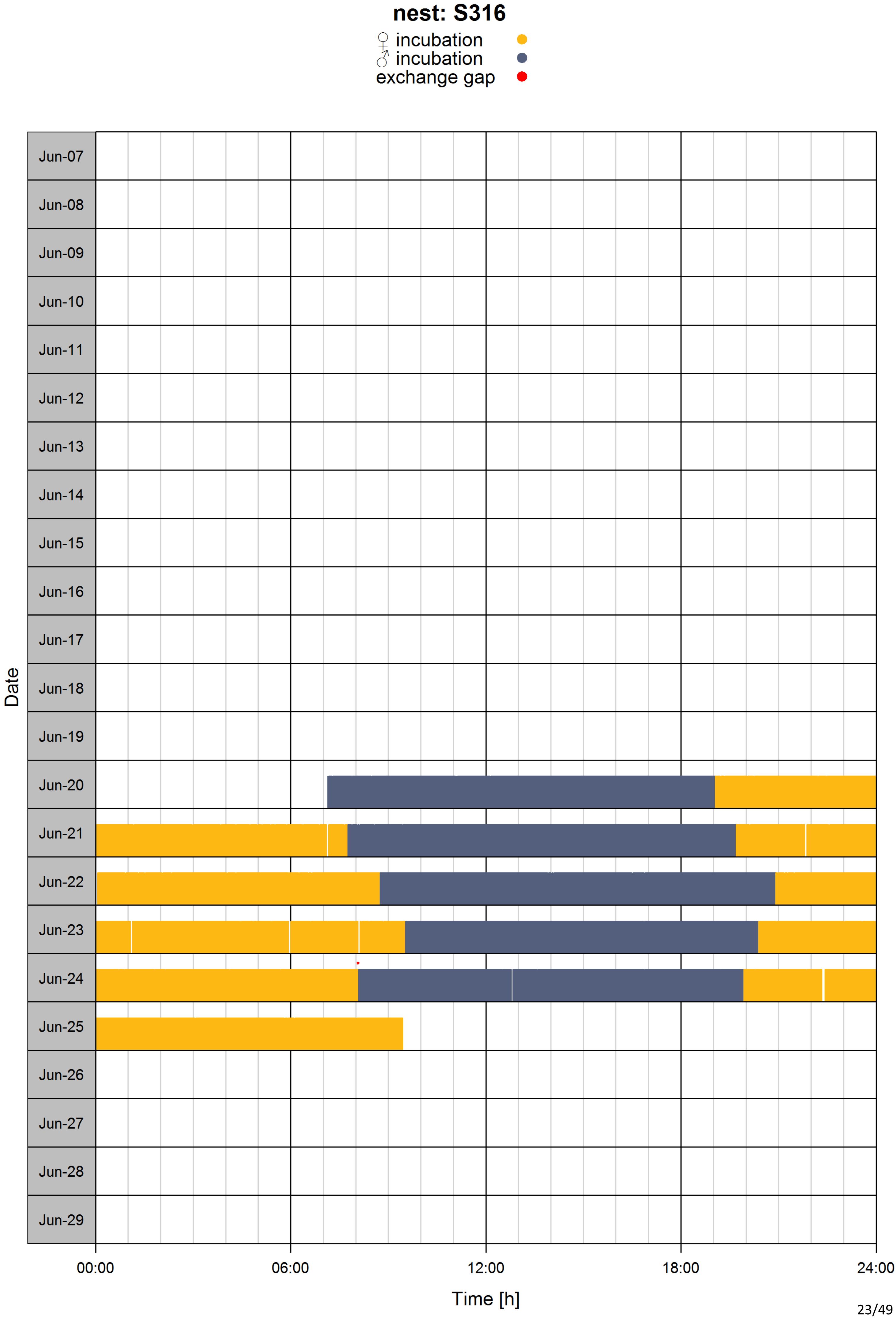
nest: S316
♀ incubation
♂ incubation
exchange gap
Date
Jun-07
Jun-08
Jun-09
Jun-10
Jun-11
Jun-12
Jun-13
Jun-14
Jun-15
Jun-16
Jun-17
Jun-18
Jun-19
Jun-20
Jun-21
Jun-22
Jun-23
Jun-24
Jun-25
Jun-26
Jun-27
Jun-28
Jun-29
00:00
06:00
12:00
18:00
24:00
Time [h]

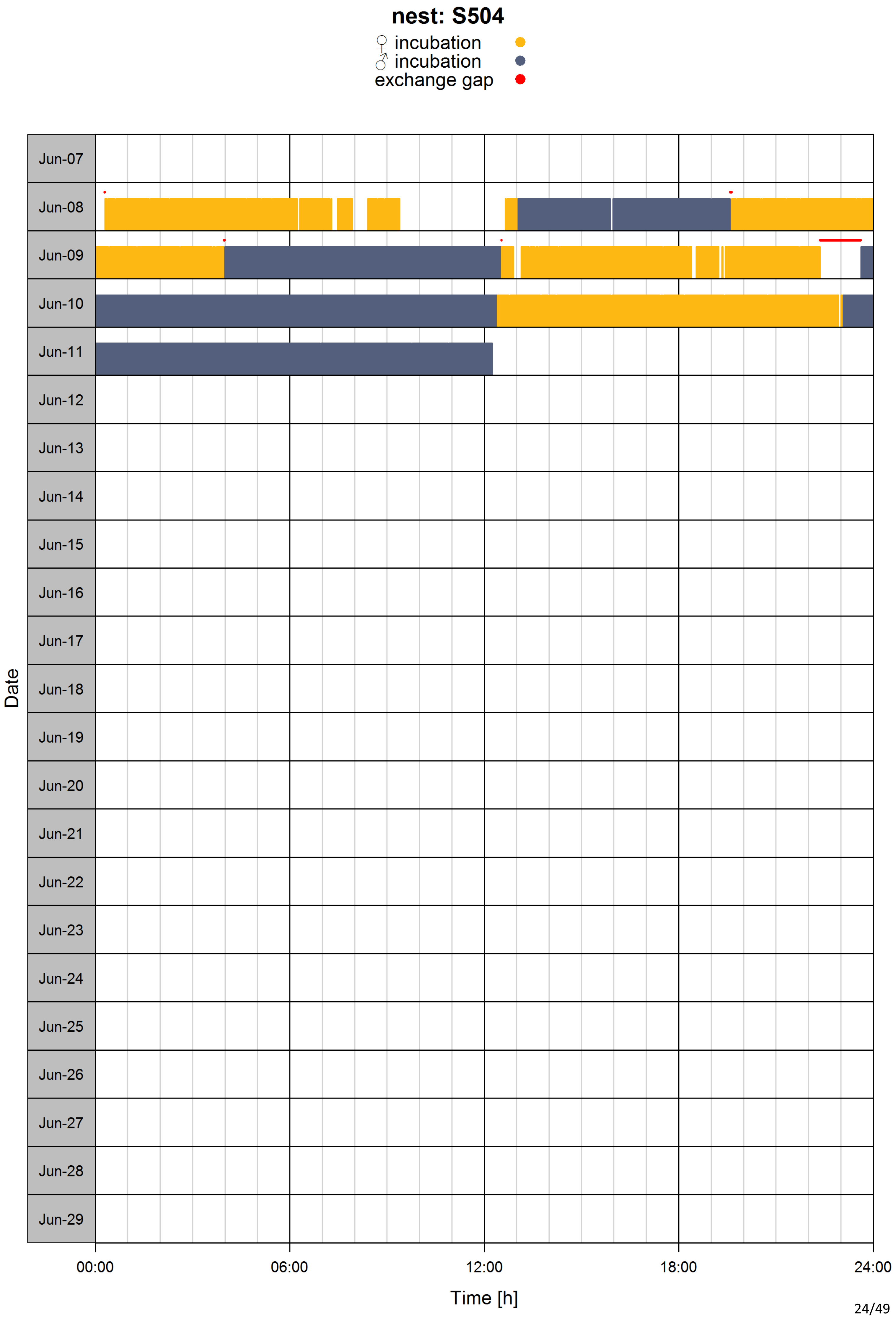
nest: S504
♀ incubation
♂ incubation
exchange gap
Date
Jun-07
Jun-08
Jun-09
Jun-10
Jun-11
Jun-12
Jun-13
Jun-14
Jun-15
Jun-16
Jun-17
Jun-18
Jun-19
Jun-20
Jun-21
Jun-22
Jun-23
Jun-24
Jun-25
Jun-26
Jun-27
Jun-28
Jun-29
00:00
06:00
12:00
18:00
24:00
Time [h]

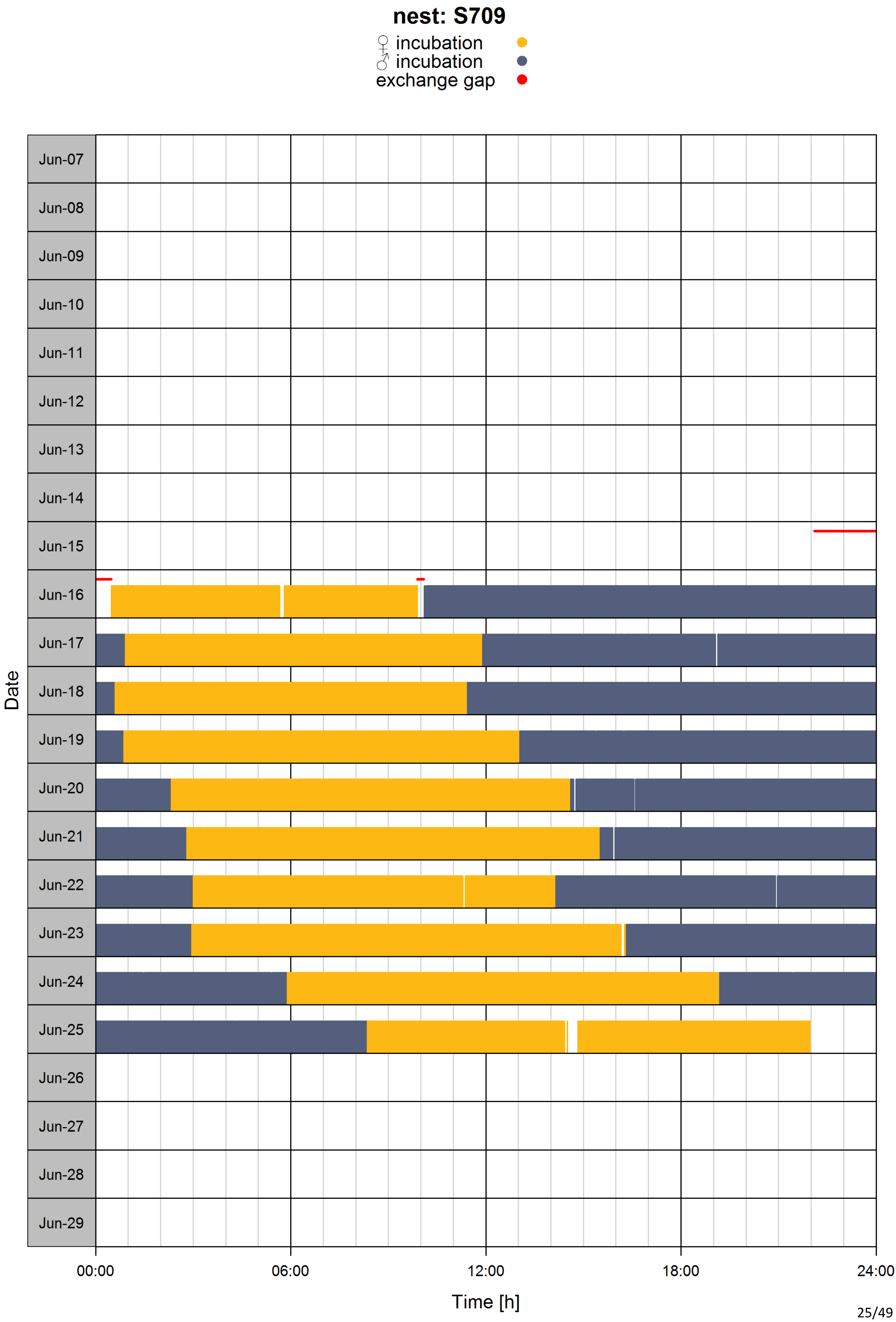

nest: S709
♀ incubation
♂ incubation
exchange gap
Date
Jun-07
Jun-08
Jun-09
Jun-10
Jun-11
Jun-12
Jun-13
Jun-14
Jun-15
Jun-16
Jun-17
Jun-18
Jun-19
Jun-20
Jun-21
Jun-22
Jun-23
Jun-24
Jun-25
Jun-26
Jun-27
Jun-28
Jun-29
00:00
06:00
12:00
18:00
24:00
Time [h]

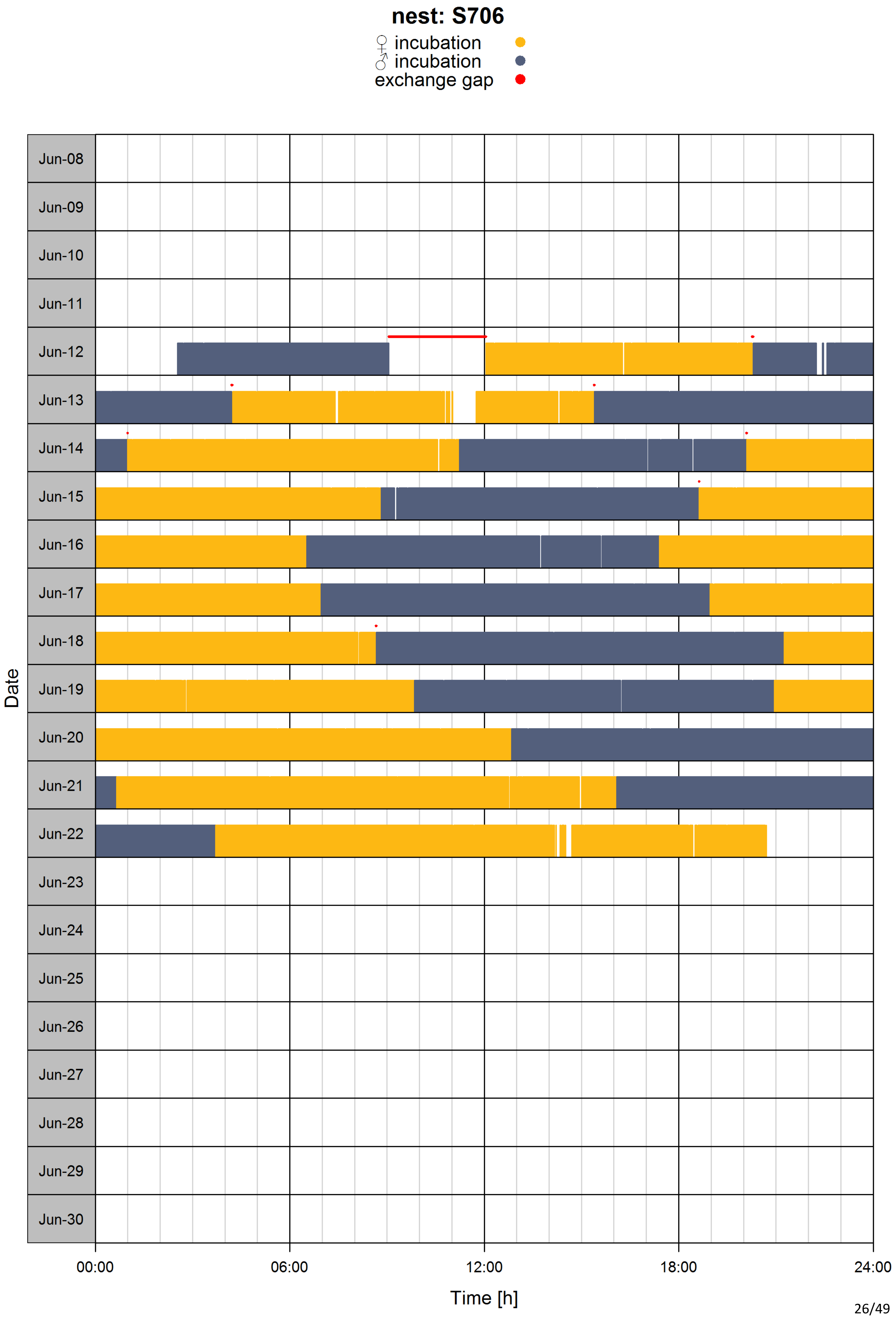
nest: S706
♀ incubation
♂ incubation
exchange gap
Date
Jun-08
Jun-09
Jun-10
Jun-11
Jun-12
Jun-13
Jun-14
Jun-15
Jun-16
Jun-17
Jun-18
Jun-19
Jun-20
Jun-21
Jun-22
Jun-23
Jun-24
Jun-25
Jun-26
Jun-27
Jun-28
Jun-29
Jun-30
00:00
06:00
12:00
18:00
24:00
Time [h]

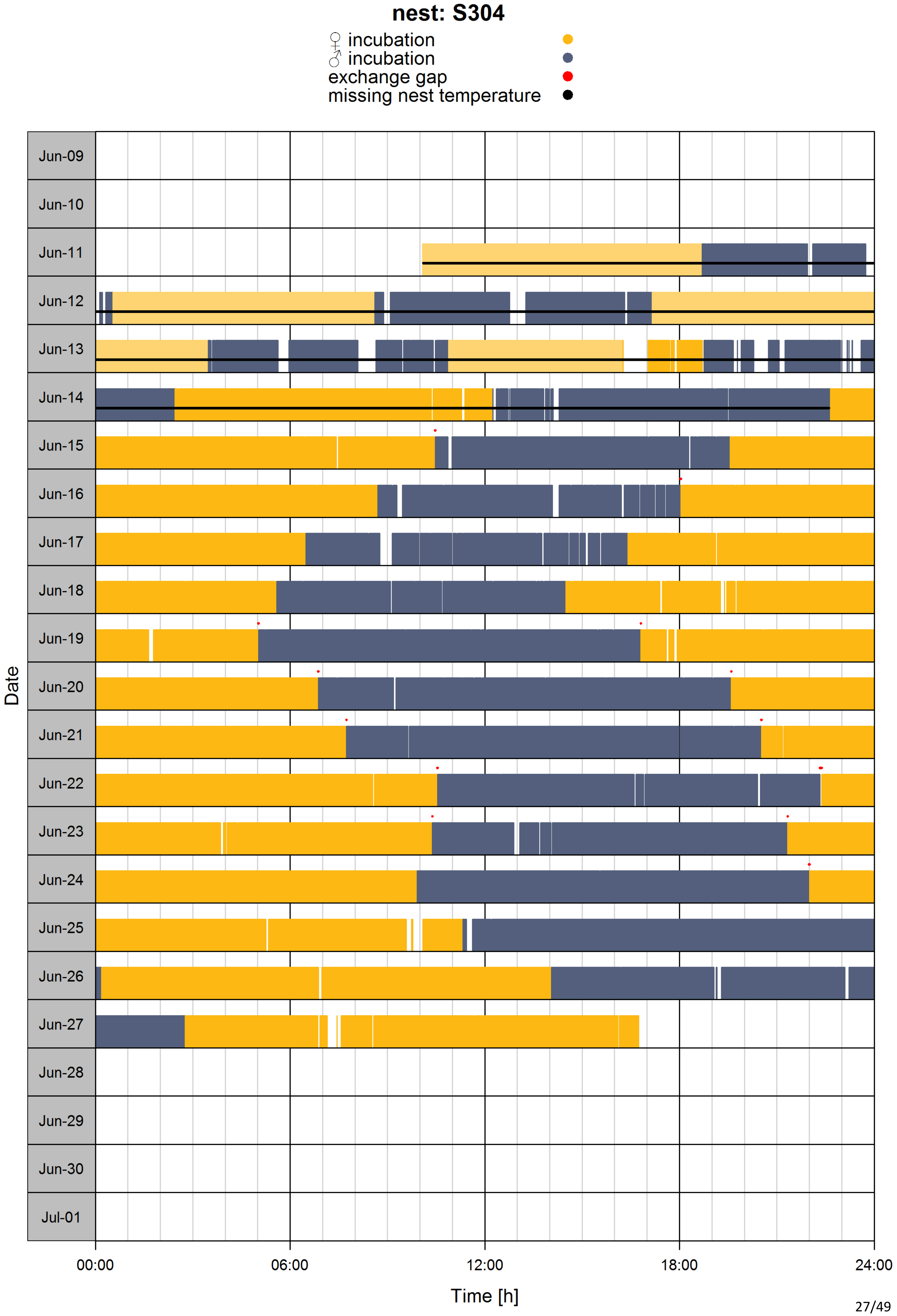
nest: S304
♀ incubation
♂ incubation
exchange gap
missing nest temperature
Date
Jun-09
Jun-10
Jun-11
Jun-12
Jun-13
Jun-14
Jun-15
Jun-16
Jun-17
Jun-18
Jun-19
Jun-20
Jun-21
Jun-22
Jun-23
Jun-24
Jun-25
Jun-26
Jun-27
Jun-28
Jun-29
Jun-30
Jul-01
00:00
06:00
12:00
18:00
24:00
Time [h]

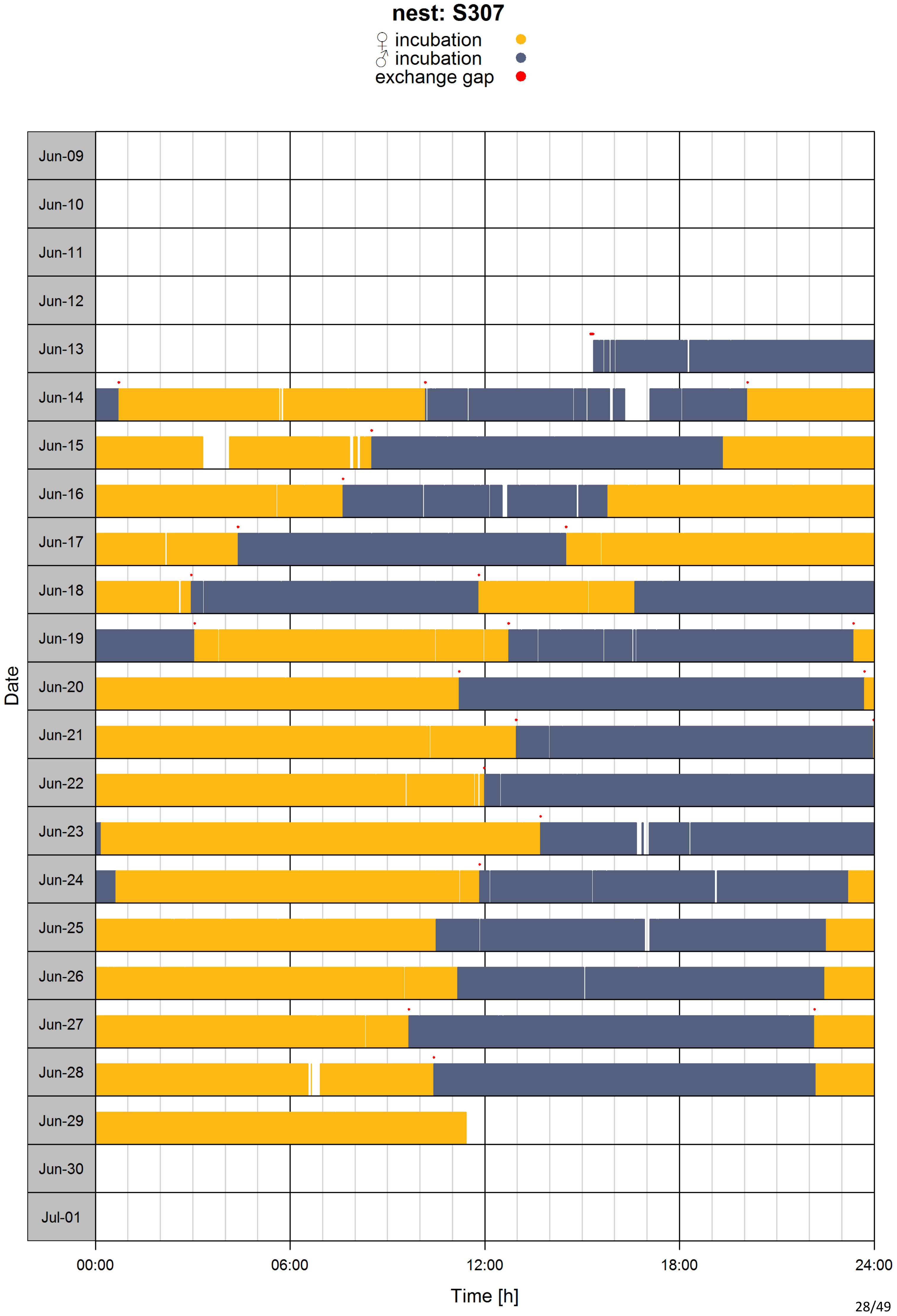
nest: S307
♀ incubation
♂ incubation
exchange gap
Date
Jun-09
Jun-10
Jun-11
Jun-12
Jun-13
Jun-14
Jun-15
Jun-16
Jun-17
Jun-18
Jun-19
Jun-20
Jun-21
Jun-22
Jun-23
Jun-24
Jun-25
Jun-26
Jun-27
Jun-28
Jun-29
Jun-30
Jul-01
00:00
06:00
12:00
18:00
24:00
Time [h]

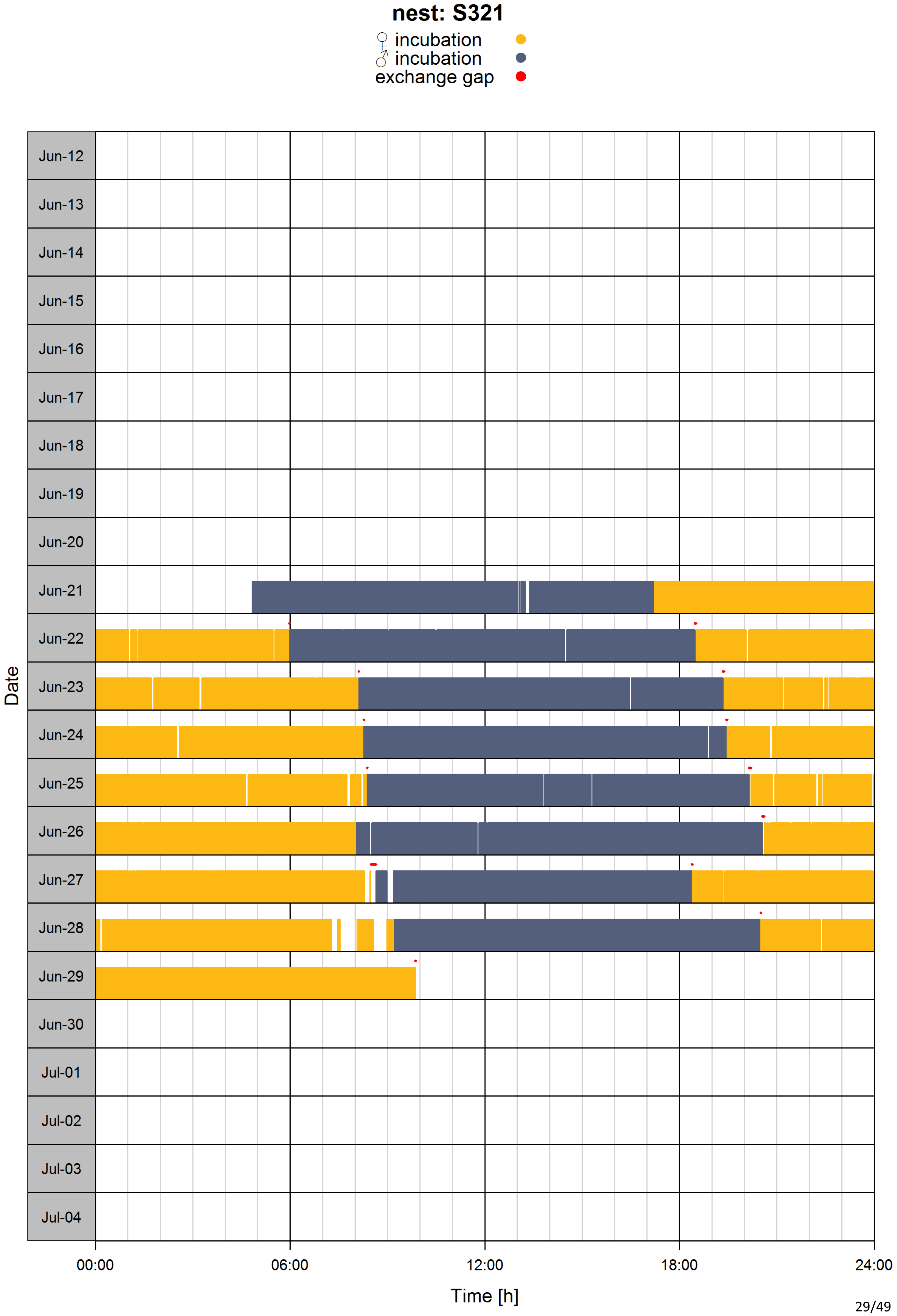
nest: S321
♀ incubation
♂ incubation
exchange gap
Date
Jun-12
Jun-13
Jun-14
Jun-15
Jun-16
Jun-17
Jun-18
Jun-19
Jun-20
Jun-21
Jun-22
Jun-23
Jun-24
Jun-25
Jun-26
Jun-27
Jun-28
Jun-29
Jun-30
Jul-01
Jul-02
Jul-03
Jul-04
00:00
06:00
12:00
18:00
24:00
Time [h]

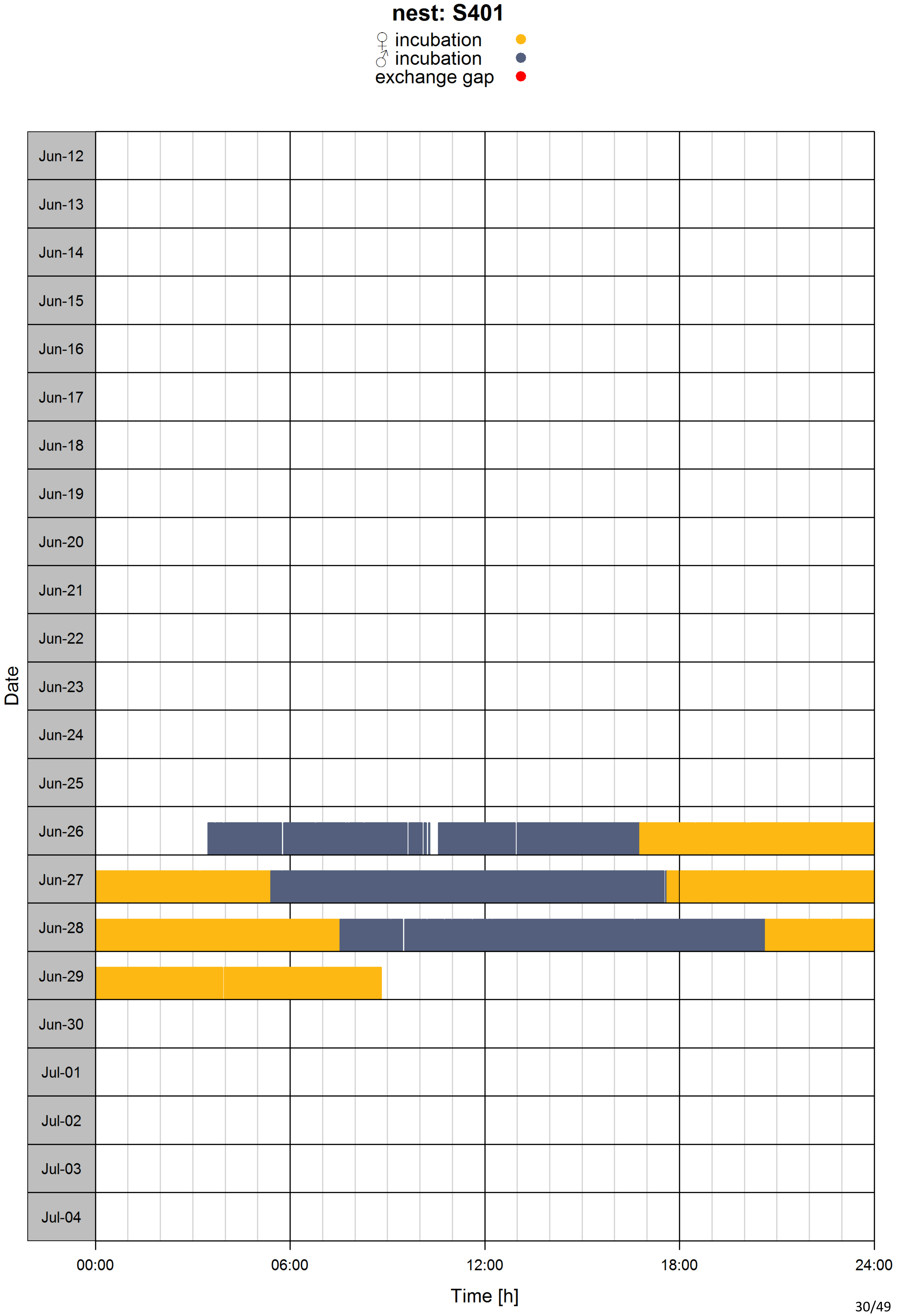
nest: S401
♀ incubation
♂ incubation
exchange gap
Date
Jun-12
Jun-13
Jun-14
Jun-15
Jun-16
Jun-17
Jun-18
Jun-19
Jun-20
Jun-21
Jun-22
Jun-23
Jun-24
Jun-25
Jun-26
Jun-27
Jun-28
Jun-29
Jun-30
Jul-01
Jul-02
Jul-03
Jul-04
00:00
06:00
12:00
18:00
24:00
Time [h]

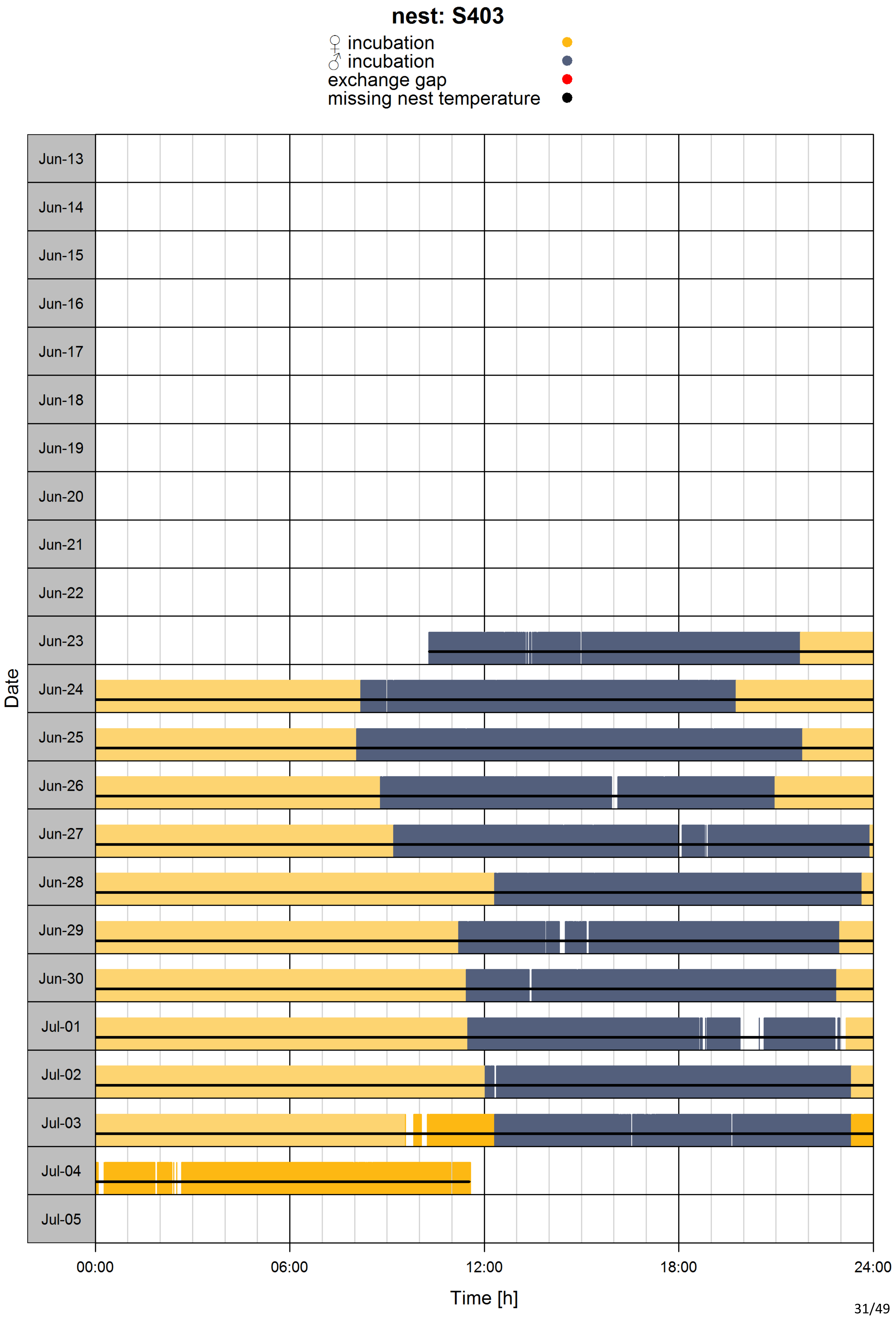
nest: S403
♀ incubation
♂ incubation
exchange gap
missing nest temperature
Date
Jun-13
Jun-14
Jun-15
Jun-16
Jun-17
Jun-18
Jun-19
Jun-20
Jun-21
Jun-22
Jun-23
Jun-24
Jun-25
Jun-26
Jun-27
Jun-28
Jun-29
Jun-30
Jul-01
Jul-02
Jul-03
Jul-04
Jul-05
00:00
06:00
12:00
18:00
24:00
Time [h]

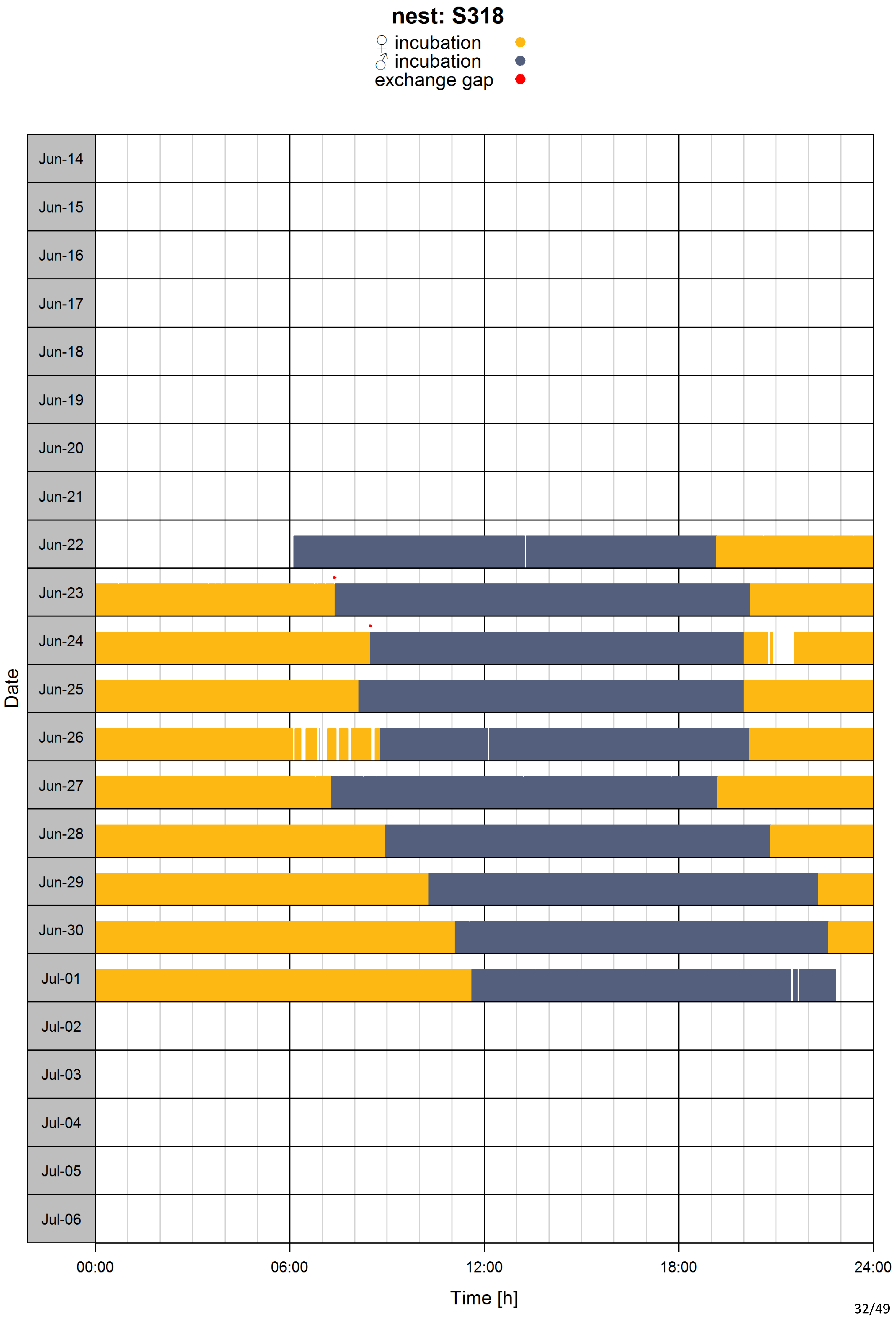
nest: S318
♀ incubation
♂ incubation
exchange gap
Date
Jun-14
Jun-15
Jun-16
Jun-17
Jun-18
Jun-19
Jun-20
Jun-21
Jun-22
Jun-23
Jun-24
Jun-25
Jun-26
Jun-27
Jun-28
Jun-29
Jun-30
Jul-01
Jul-02
Jul-03
Jul-04
Jul-05
Jul-06
00:00
06:00
12:00
18:00
24:00
Time [h]

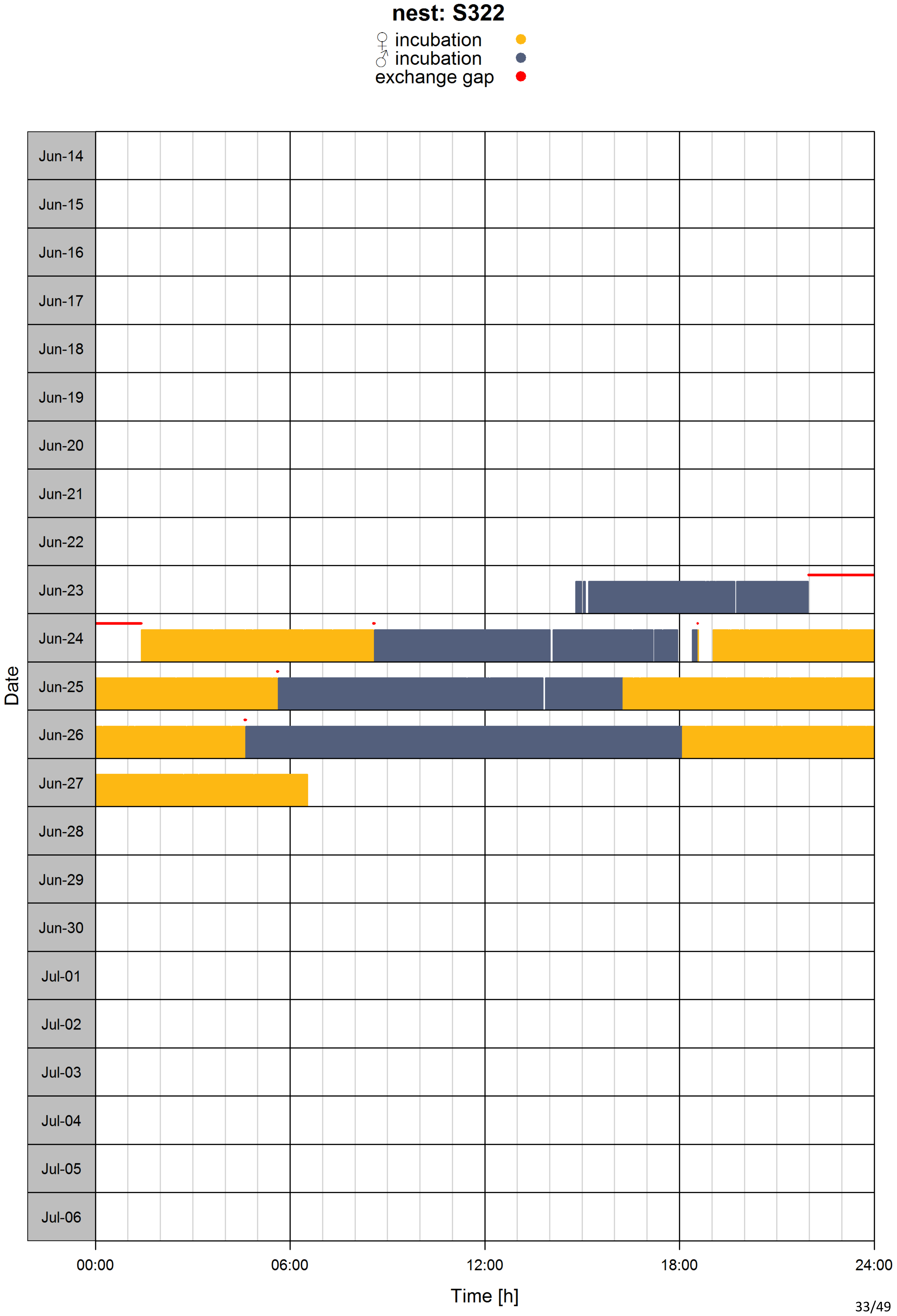

nest: S322
♀ incubation
♂ incubation
exchange gap
Date
Jun-14
Jun-15
Jun-16
Jun-17
Jun-18
Jun-19
Jun-20
Jun-21
Jun-22
Jun-23
Jun-24
Jun-25
Jun-26
Jun-27
Jun-28
Jun-29
Jun-30
Jul-01
Jul-02
Jul-03
Jul-04
Jul-05
Jul-06
00:00
06:00
12:00
18:00
24:00
Time [h]

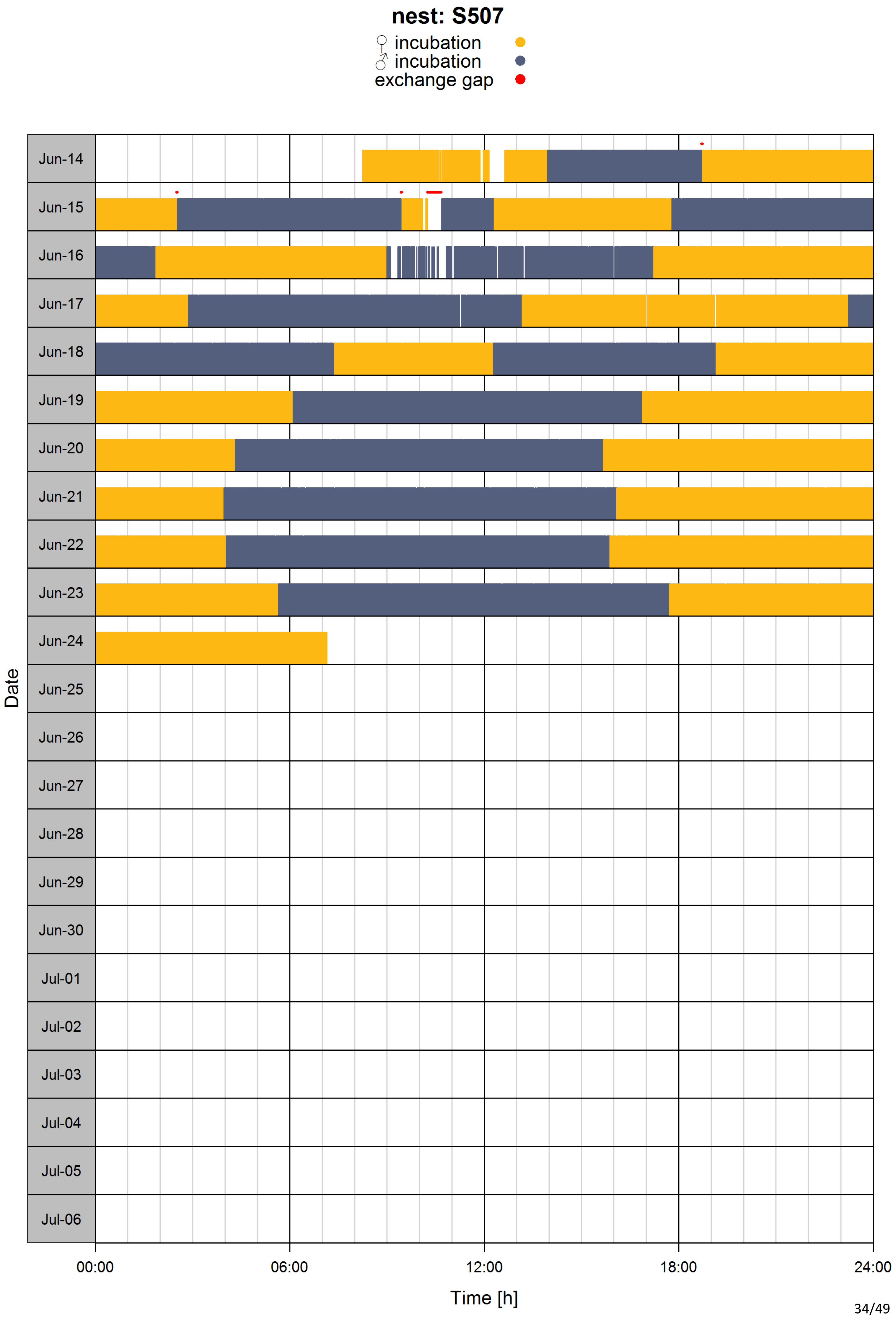

nest: S507
♀ incubation
♂ incubation
exchange gap
Date
Jun-14
Jun-15
Jun-16
Jun-17
Jun-18
Jun-19
Jun-20
Jun-21
Jun-22
Jun-23
Jun-24
Jun-25
Jun-26
Jun-27
Jun-28
Jun-29
Jun-30
Jul-01
Jul-02
Jul-03
Jul-04
Jul-05
Jul-06
00:00
06:00
12:00
18:00
24:00
Time [h]

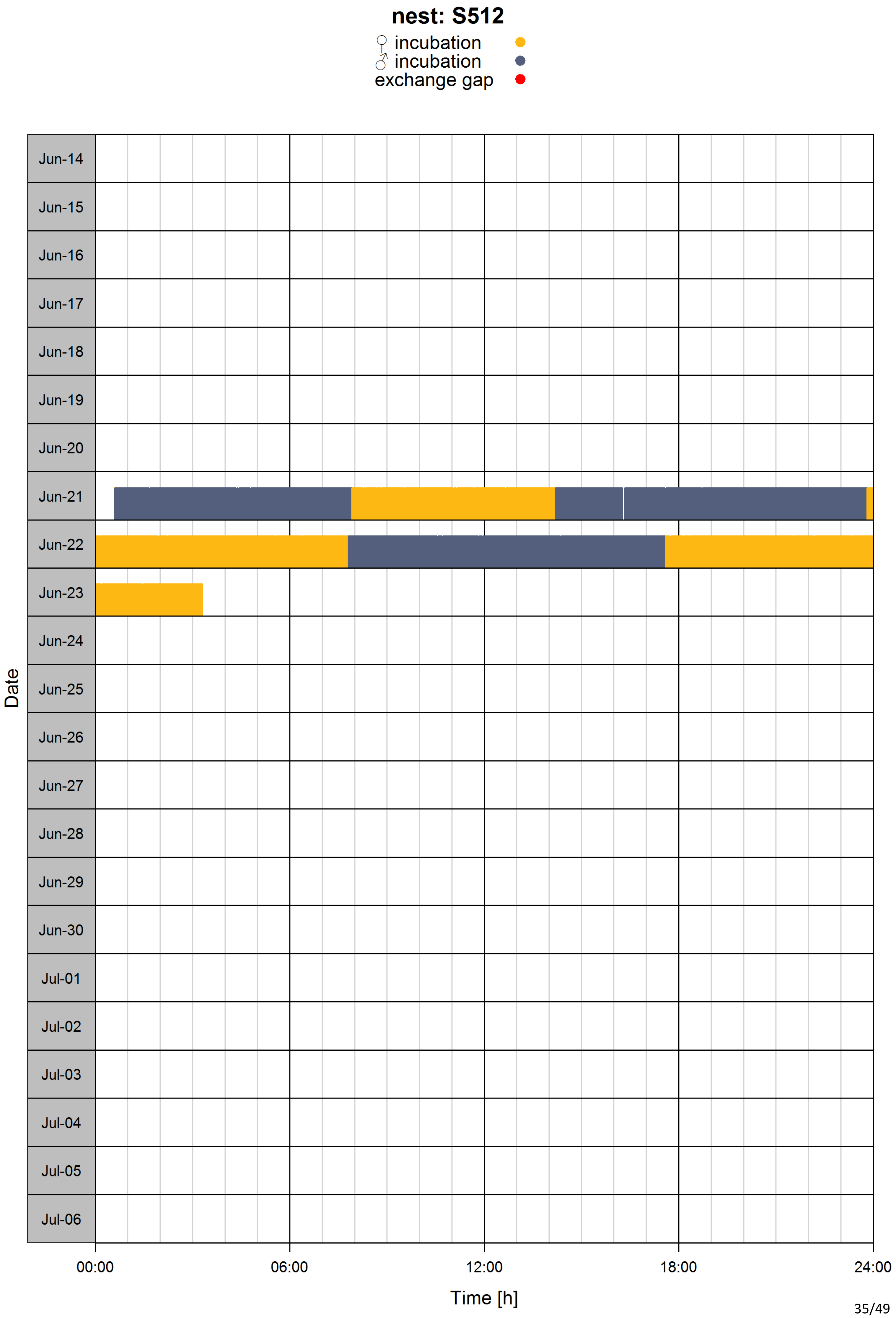
nest: S512
♀ incubation
♂ incubation
exchange gap
Date
Jun-14
Jun-15
Jun-16
Jun-17
Jun-18
Jun-19
Jun-20
Jun-21
Jun-22
Jun-23
Jun-24
Jun-25
Jun-26
Jun-27
Jun-28
Jun-29
Jun-30
Jul-01
Jul-02
Jul-03
Jul-04
Jul-05
Jul-06
00:00
06:00
12:00
18:00
24:00
Time [h]

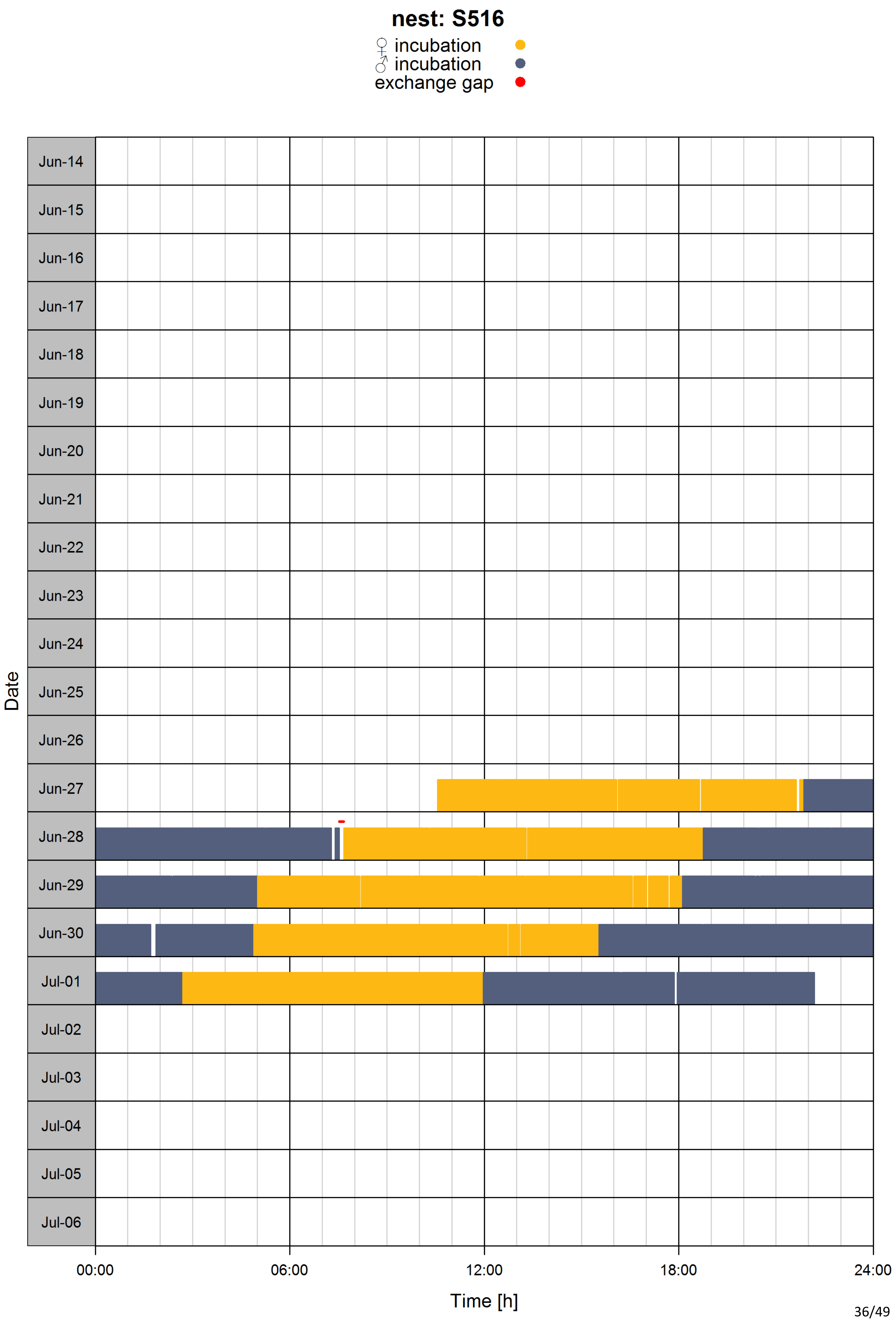
nest: S516
♀ incubation
♂ incubation
exchange gap
Date
Jun-14
Jun-15
Jun-16
Jun-17
Jun-18
Jun-19
Jun-20
Jun-21
Jun-22
Jun-23
Jun-24
Jun-25
Jun-26
Jun-27
Jun-28
Jun-29
Jun-30
Jul-01
Jul-02
Jul-03
Jul-04
Jul-05
Jul-06
00:00
06:00
12:00
18:00
24:00
Time [h]

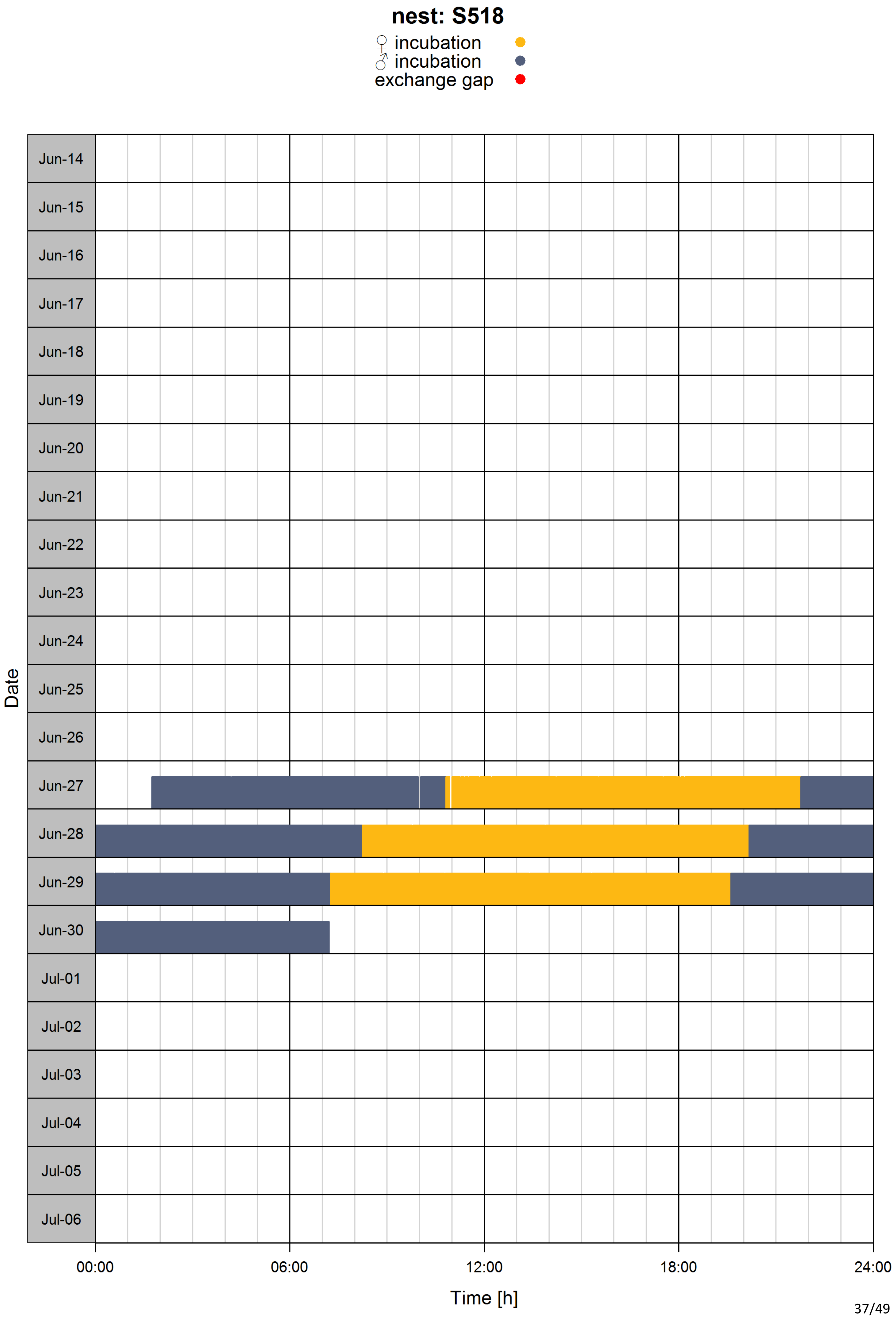
nest: S518
♀ incubation
♂ incubation
exchange gap
Date
Jun-14
Jun-15
Jun-16
Jun-17
Jun-18
Jun-19
Jun-20
Jun-21
Jun-22
Jun-23
Jun-24
Jun-25
Jun-26
Jun-27
Jun-28
Jun-29
Jun-30
Jul-01
Jul-02
Jul-03
Jul-04
Jul-05
Jul-06
00:00
06:00
12:00
18:00
24:00
Time [h]

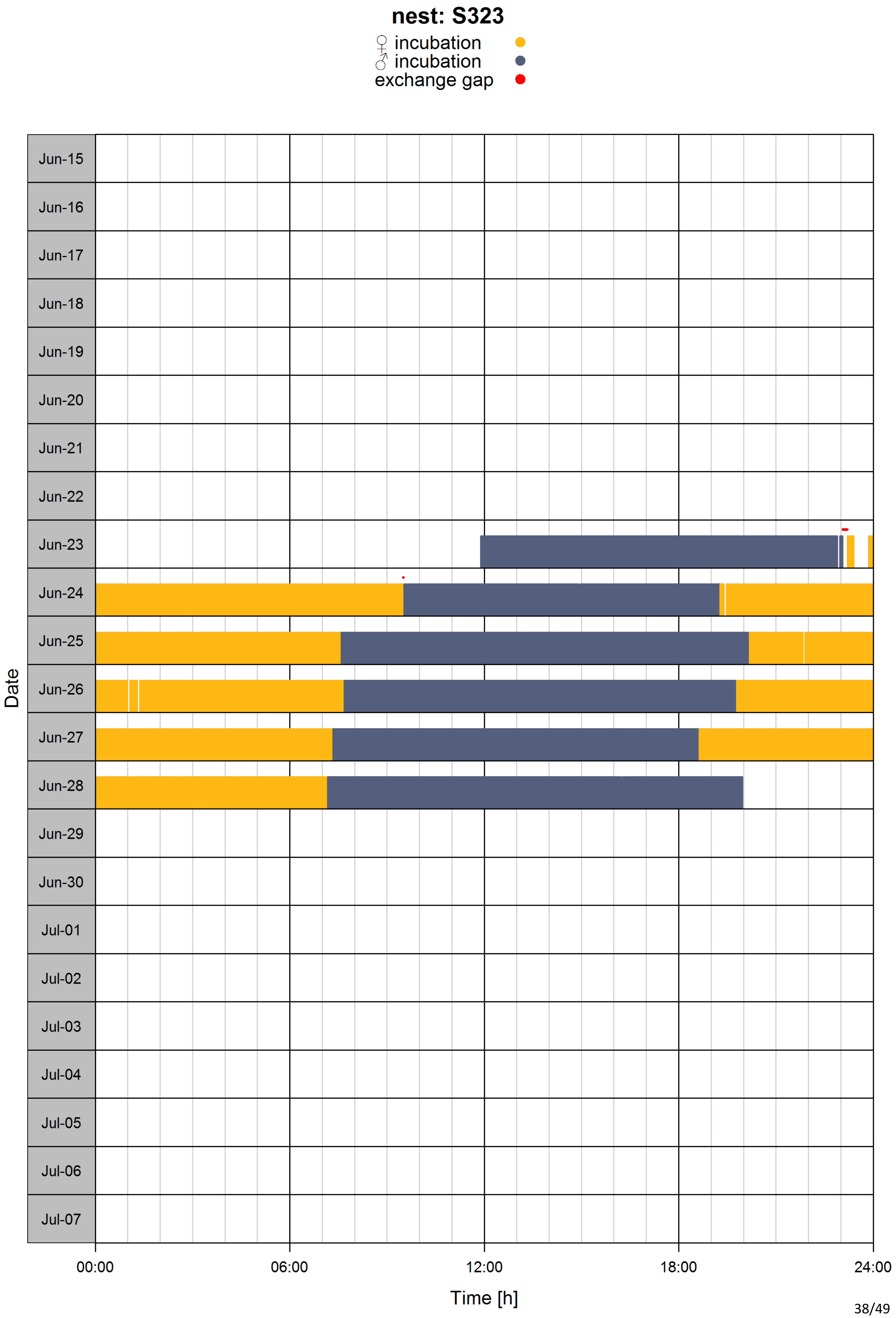
nest: S323
♀ incubation
♂ incubation
exchange gap
Date
Jun-15
Jun-16
Jun-17
Jun-18
Jun-19
Jun-20
Jun-21
Jun-22
Jun-23
Jun-24
Jun-25
Jun-26
Jun-27
Jun-28
Jun-29
Jun-30
Jul-01
Jul-02
Jul-03
Jul-04
Jul-05
Jul-06
Jul-07
00:00
06:00
12:00
18:00
24:00
Time [h]

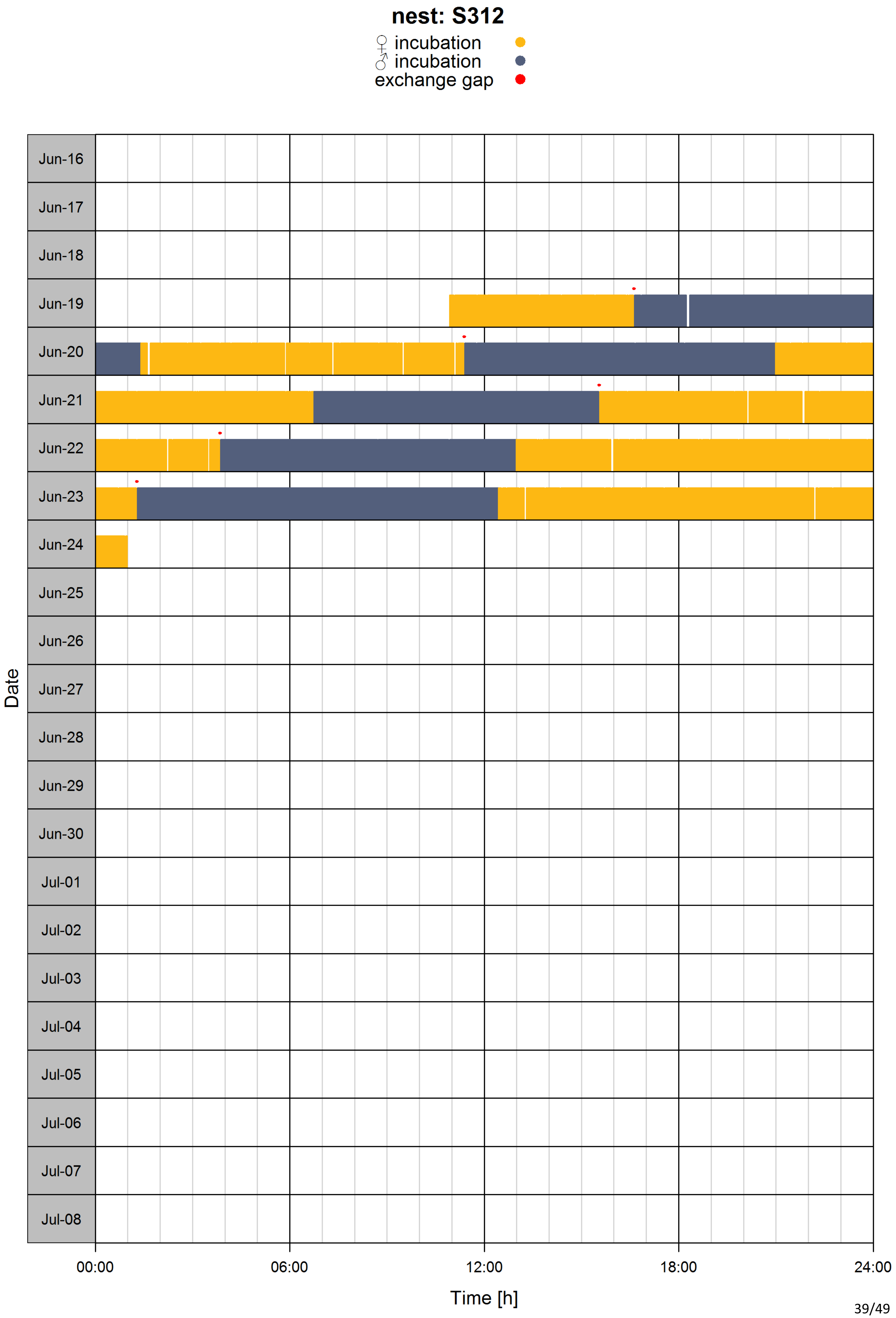
nest: S312
♀ incubation
♂ incubation
exchange gap
Date
Jun-16
Jun-17
Jun-18
Jun-19
Jun-20
Jun-21
Jun-22
Jun-23
Jun-24
Jun-25
Jun-26
Jun-27
Jun-28
Jun-29
Jun-30
Jul-01
Jul-02
Jul-03
Jul-04
Jul-05
Jul-06
Jul-07
Jul-08
00:00
06:00
12:00
18:00
24:00
Time [h]

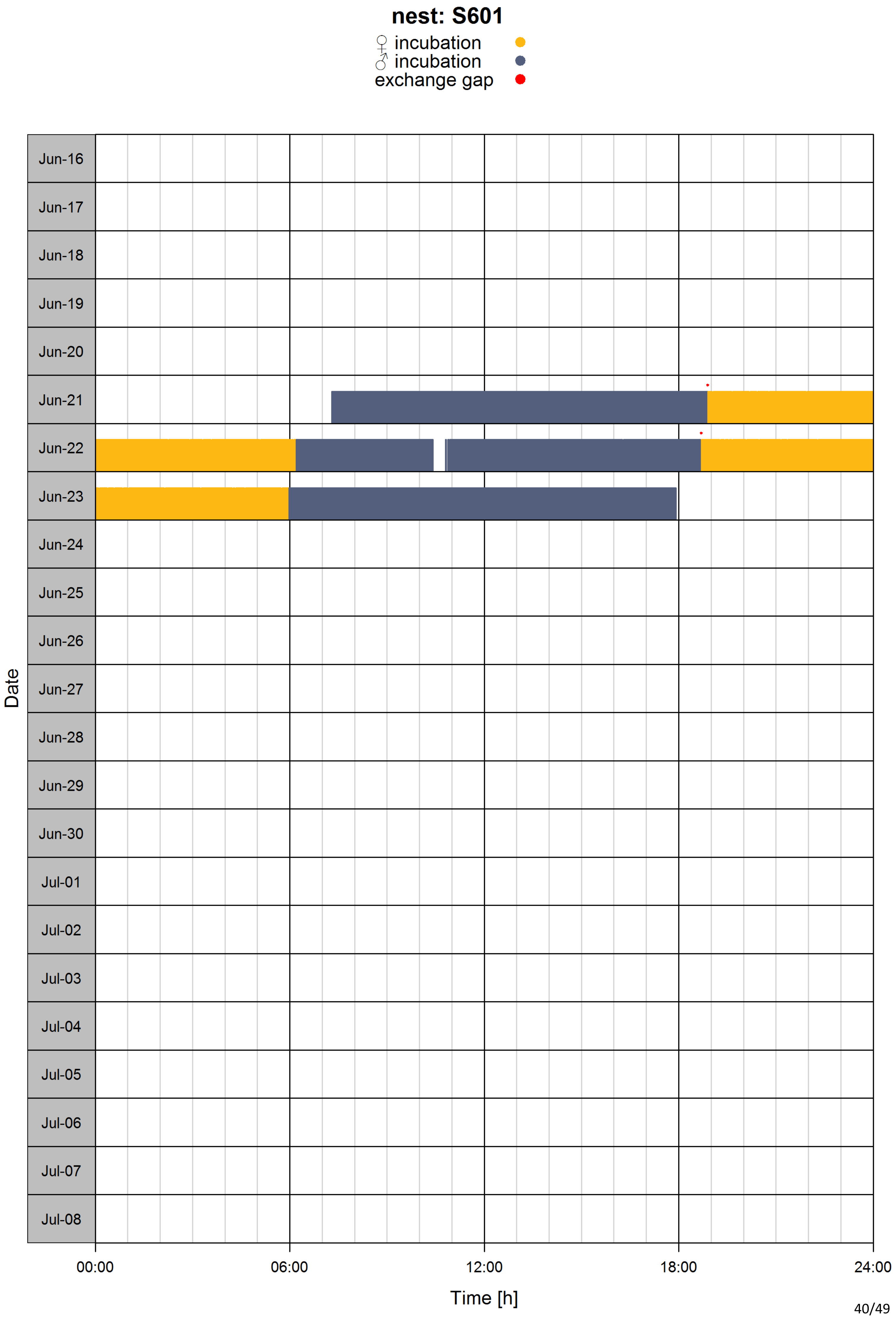

nest: S601
♀ incubation
♂ incubation
exchange gap
Date
Jun-16
Jun-17
Jun-18
Jun-19
Jun-20
Jun-21
Jun-22
Jun-23
Jun-24
Jun-25
Jun-26
Jun-27
Jun-28
Jun-29
Jun-30
Jul-01
Jul-02
Jul-03
Jul-04
Jul-05
Jul-06
Jul-07
Jul-08
00:00
06:00
12:00
18:00
24:00
Time [h]

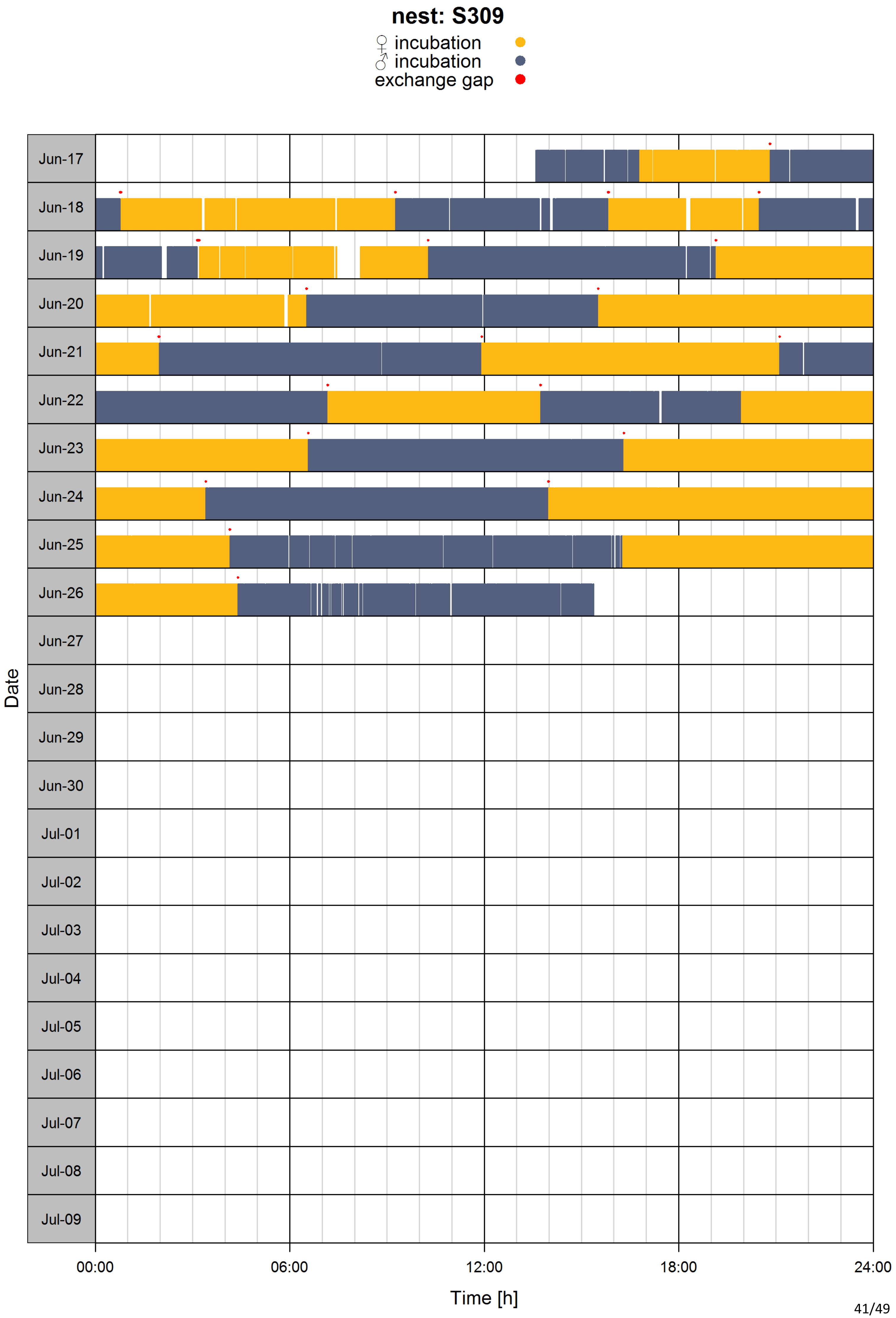
nest: S309
♀ incubation
♂ incubation
exchange gap
Date
Jun-17
Jun-18
Jun-19
Jun-20
Jun-21
Jun-22
Jun-23
Jun-24
Jun-25
Jun-26
Jun-27
Jun-28
Jun-29
Jun-30
Jul-01
Jul-02
Jul-03
Jul-04
Jul-05
Jul-06
Jul-07
Jul-08
Jul-09
00:00
06:00
12:00
18:00
24:00
Time [h]

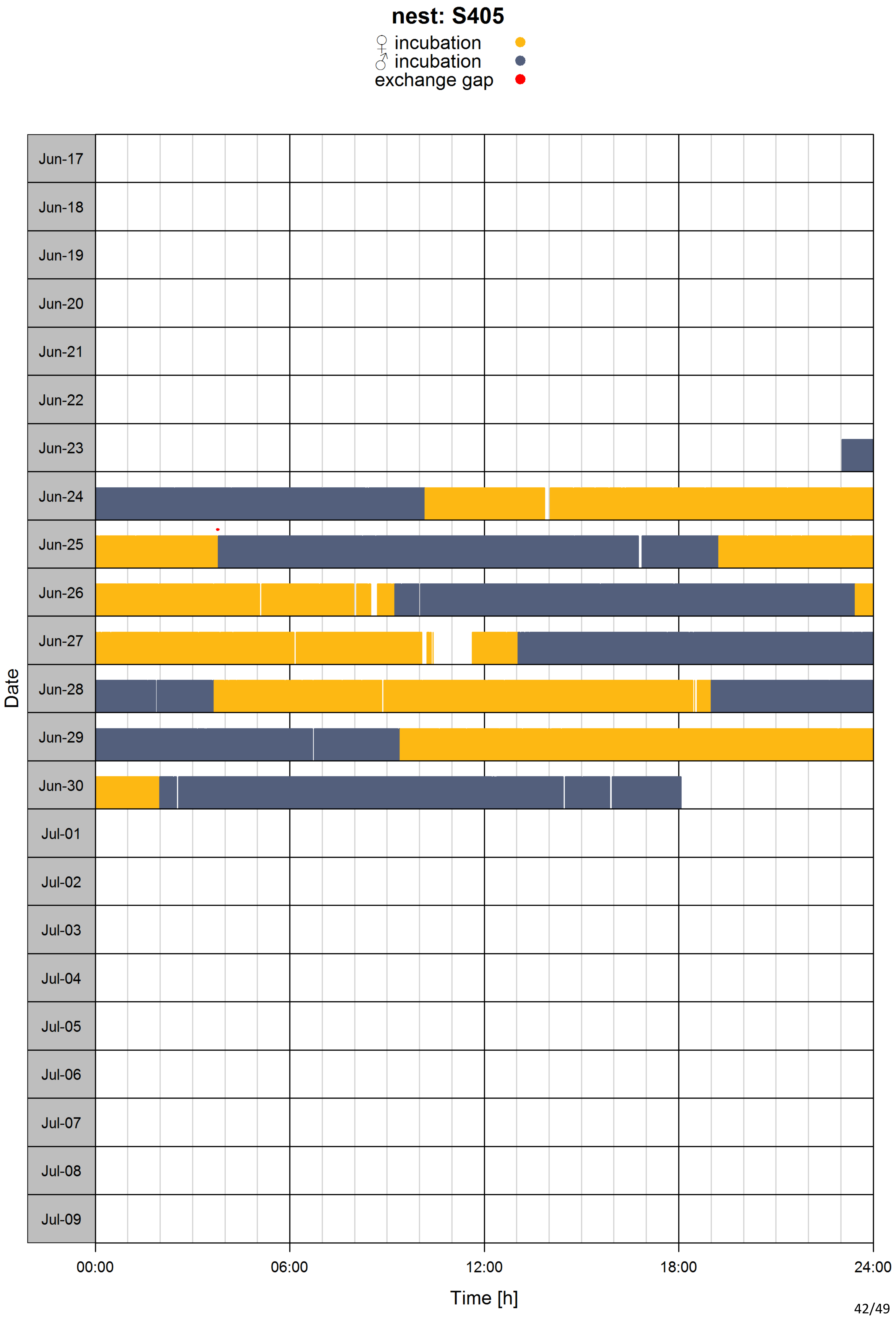
nest: S405
♀ incubation
♂ incubation
exchange gap
Jun-17
Jun-18
Jun-19
Jun-20
Jun-21
Jun-22
Jun-23
Jun-24
Jun-25
Jun-26
Jun-27
Jun-28
Jun-29
Jun-30
Jul-01
Jul-02
Jul-03
Jul-04
Jul-05
Jul-06
Jul-07
Jul-08
Jul-09
Date
00:00
06:00
12:00
18:00
24:00
Time [h]

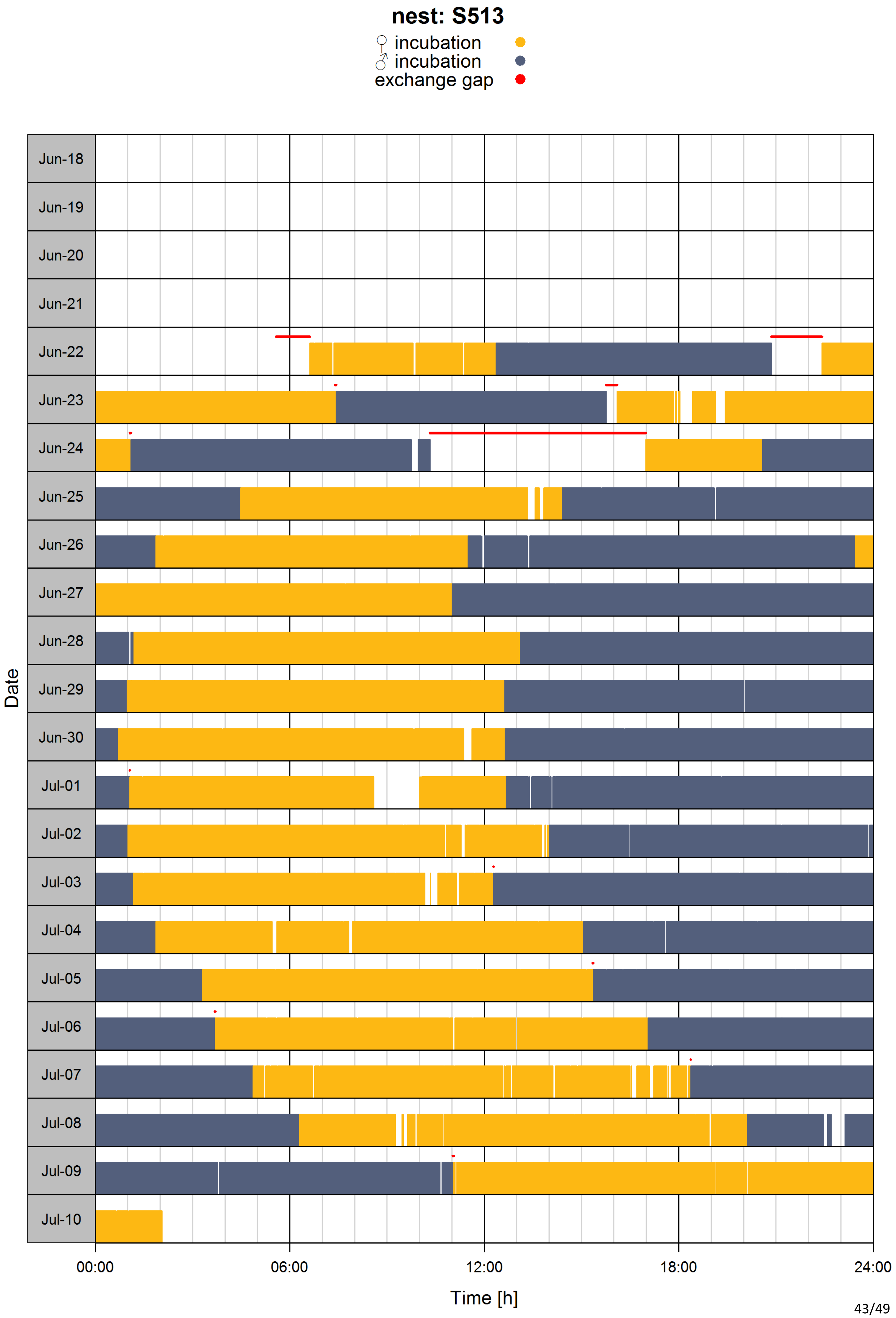
nest: S513
♀ incubation
♂ incubation
exchange gap
Jun-18
Jun-19
Jun-20
Jun-21
Jun-22
Jun-23
Jun-24
Jun-25
Jun-26
Jun-27
Jun-28
Jun-29
Jun-30
Jul-01
Jul-02
Jul-03
Jul-04
Jul-05
Jul-06
Jul-07
Jul-08
Jul-09
Jul-10
Date
00:00
06:00
12:00
18:00
24:00
Time [h]

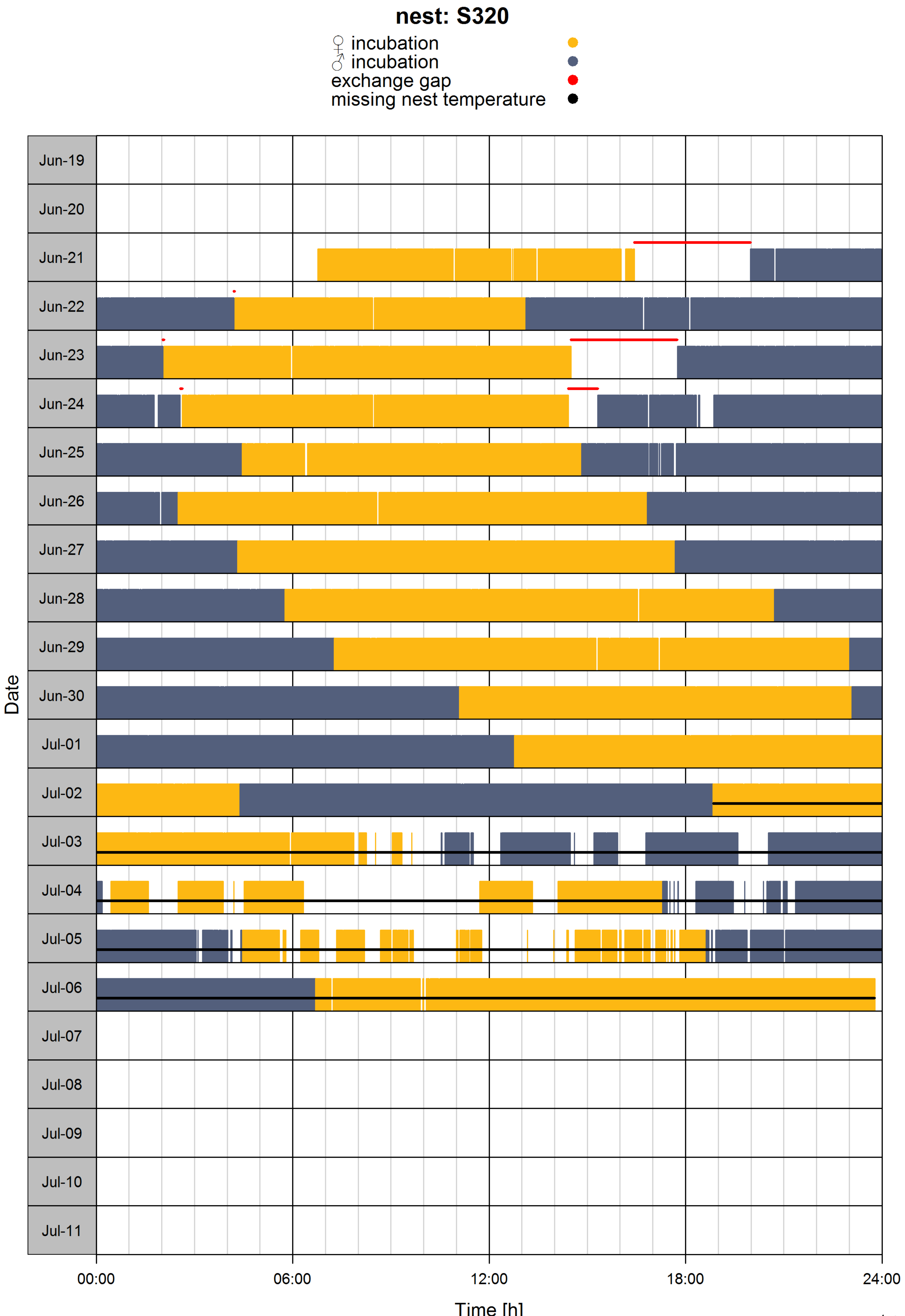

nest: S320
♀ incubation
♂ incubation
exchange gap
missing nest temperature
Jun-19
Jun-20
Jun-21
Jun-22
Jun-23
Jun-24
Jun-25
Jun-26
Jun-27
Jun-28
Jun-29
Jun-30
Jul-01
Jul-02
Jul-03
Jul-04
Jul-05
Jul-06
Jul-07
Jul-08
Jul-09
Jul-10
Jul-11
Date
00:00
06:00
12:00
18:00
24:00
Time [h]

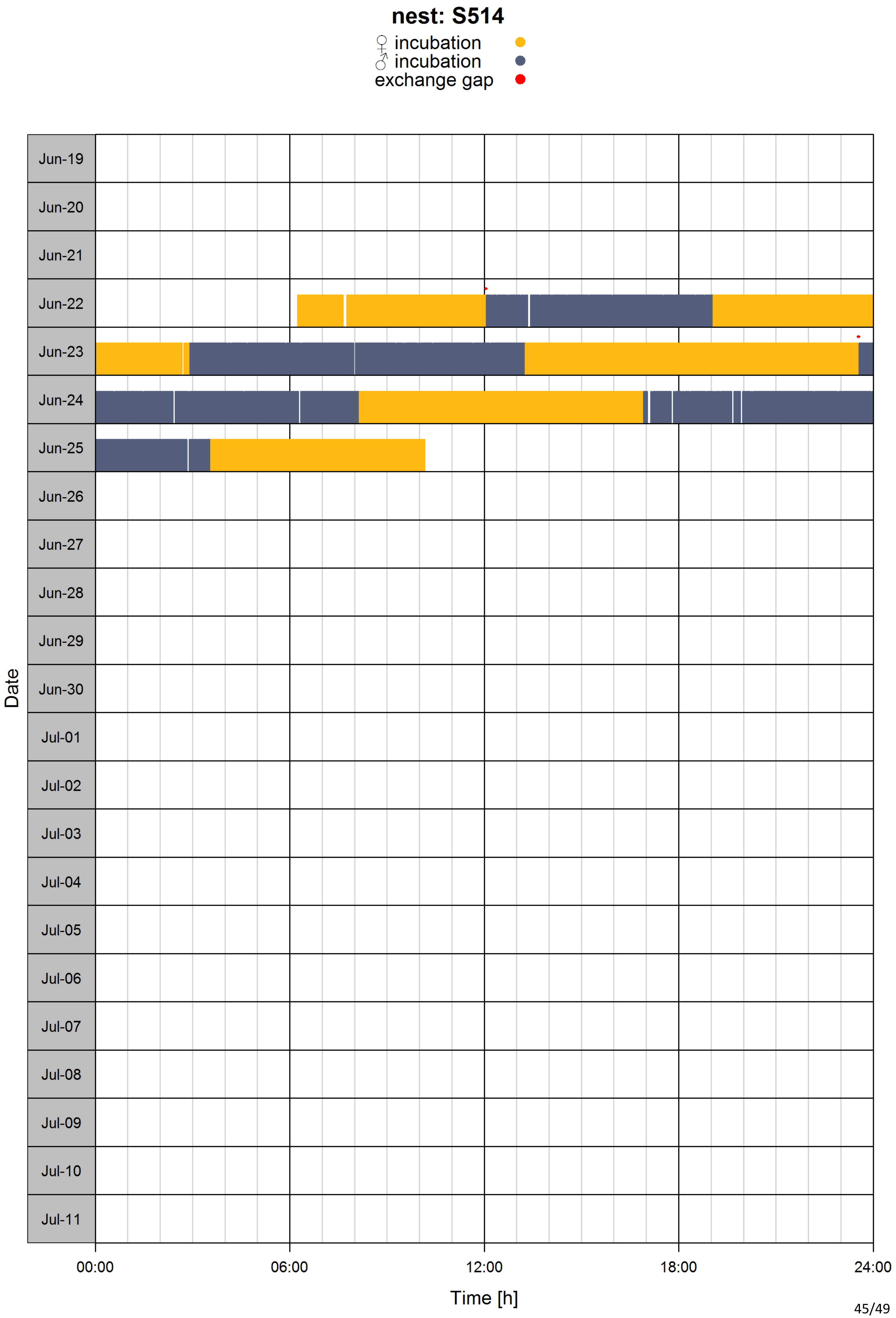
nest: S514
♀ incubation
♂ incubation
exchange gap
Date
Jun-19
Jun-20
Jun-21
Jun-22
Jun-23
Jun-24
Jun-25
Jun-26
Jun-27
Jun-28
Jun-29
Jun-30
Jul-01
Jul-02
Jul-03
Jul-04
Jul-05
Jul-06
Jul-07
Jul-08
Jul-09
Jul-10
Jul-11
00:00
06:00
12:00
18:00
24:00
Time [h]

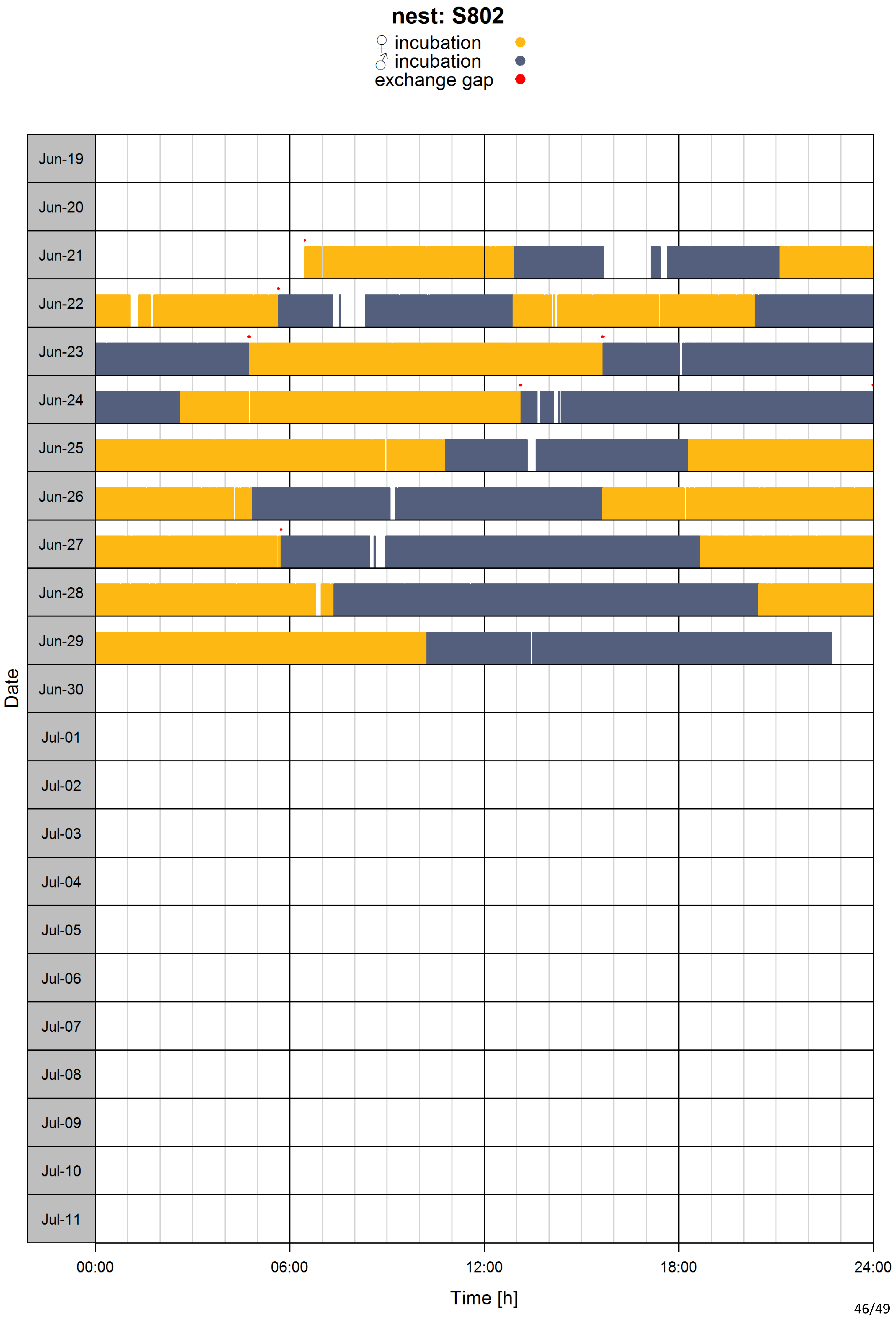

nest: S802
♀ incubation
♂ incubation
exchange gap
Date
Jun-19
Jun-20
Jun-21
Jun-22
Jun-23
Jun-24
Jun-25
Jun-26
Jun-27
Jun-28
Jun-29
Jun-30
Jul-01
Jul-02
Jul-03
Jul-04
Jul-05
Jul-06
Jul-07
Jul-08
Jul-09
Jul-10
Jul-11
00:00
06:00
12:00
18:00
24:00
Time [h]

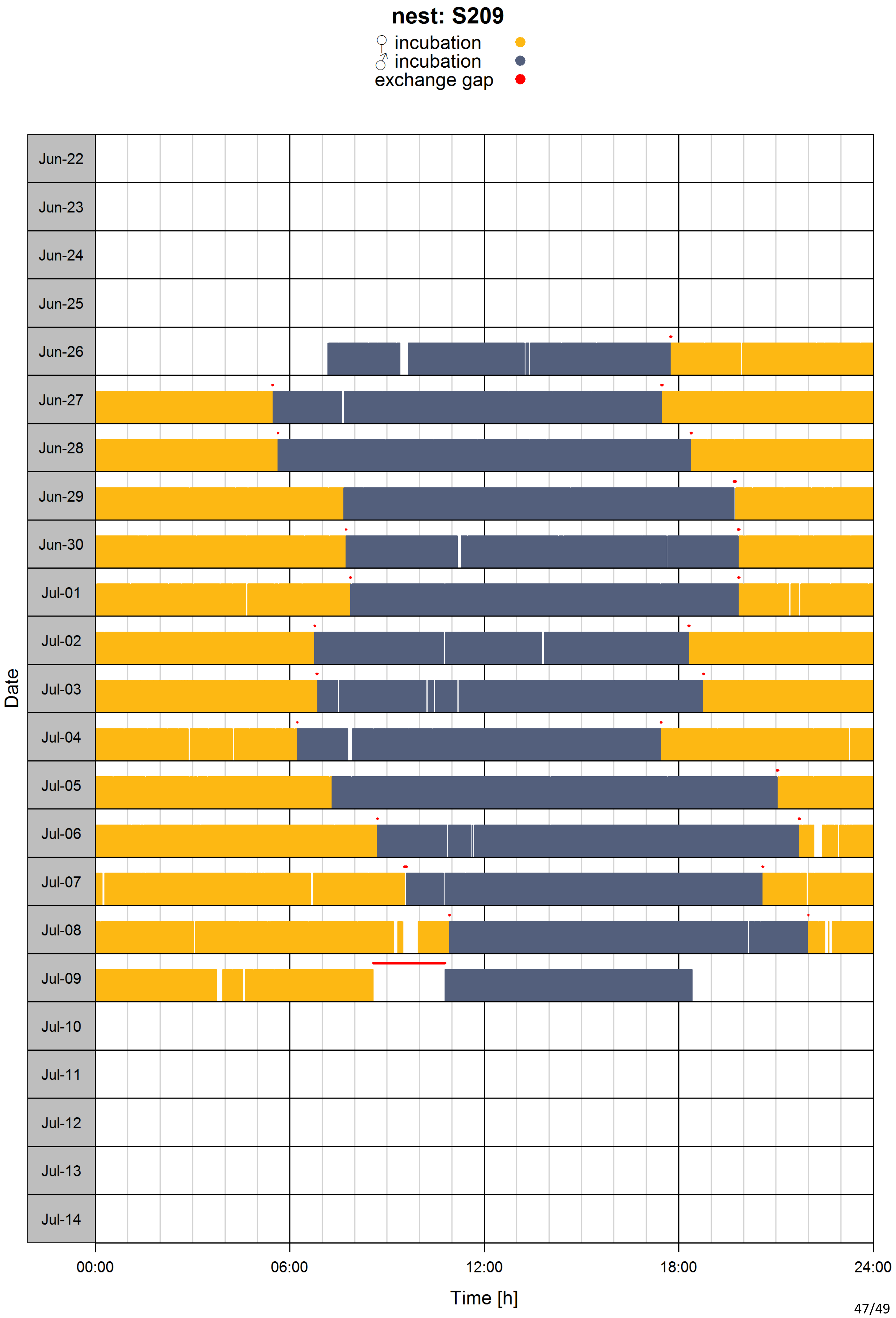

nest: S209
♀ incubation
♂ incubation
exchange gap
Date
Jun-22
Jun-23
Jun-24
Jun-25
Jun-26
Jun-27
Jun-28
Jun-29
Jun-30
Jul-01
Jul-02
Jul-03
Jul-04
Jul-05
Jul-06
Jul-07
Jul-08
Jul-09
Jul-10
Jul-11
Jul-12
Jul-13
Jul-14
00:00
06:00
12:00
18:00
24:00
Time [h]

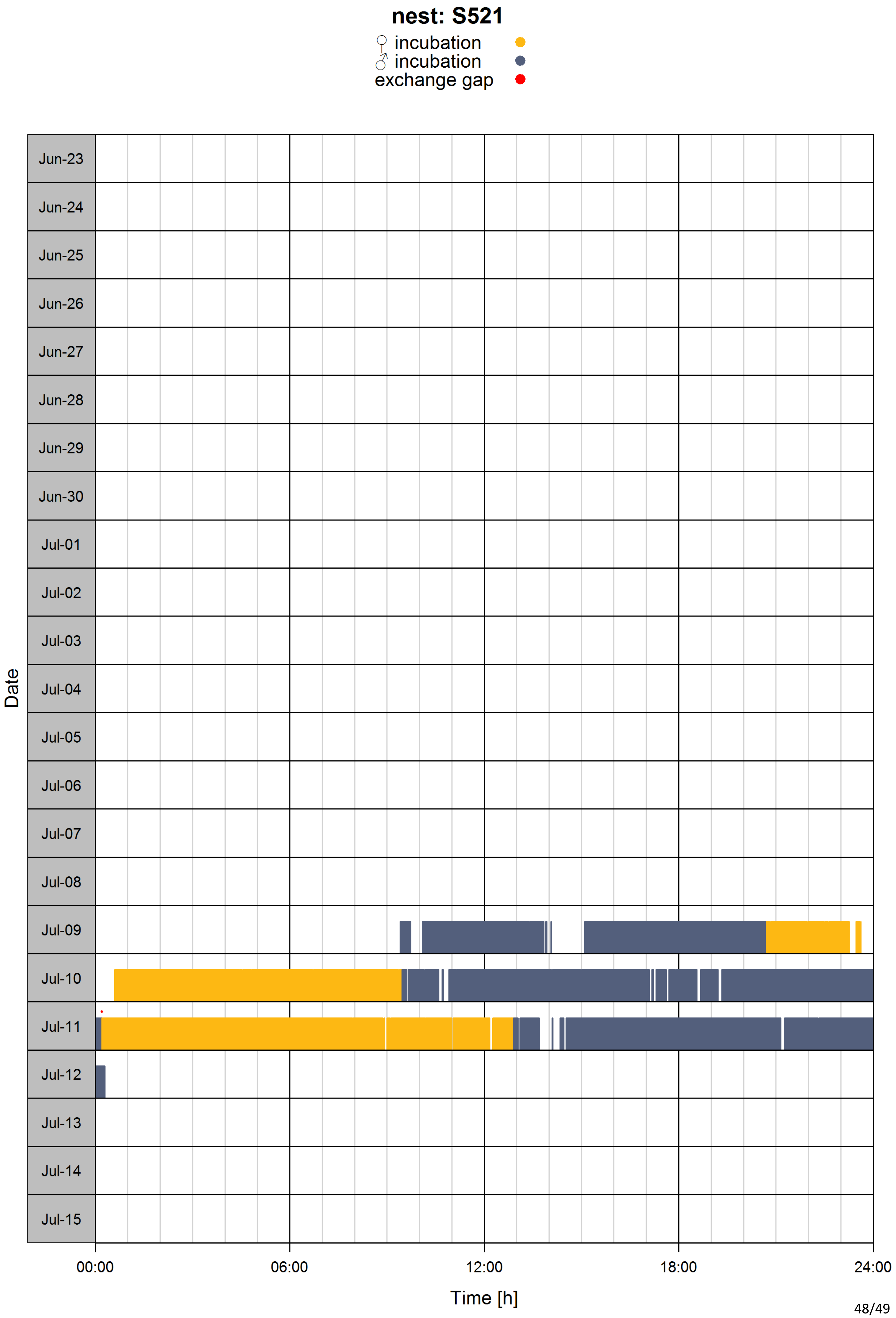
nest: S521
♀ incubation
♂ incubation
exchange gap
Date
Jun-23
Jun-24
Jun-25
Jun-26
Jun-27
Jun-28
Jun-29
Jun-30
Jul-01
Jul-02
Jul-03
Jul-04
Jul-05
Jul-06
Jul-07
Jul-08
Jul-09
Jul-10
Jul-11
Jul-12
Jul-13
Jul-14
Jul-15
00:00
06:00
12:00
18:00
24:00
Time [h]

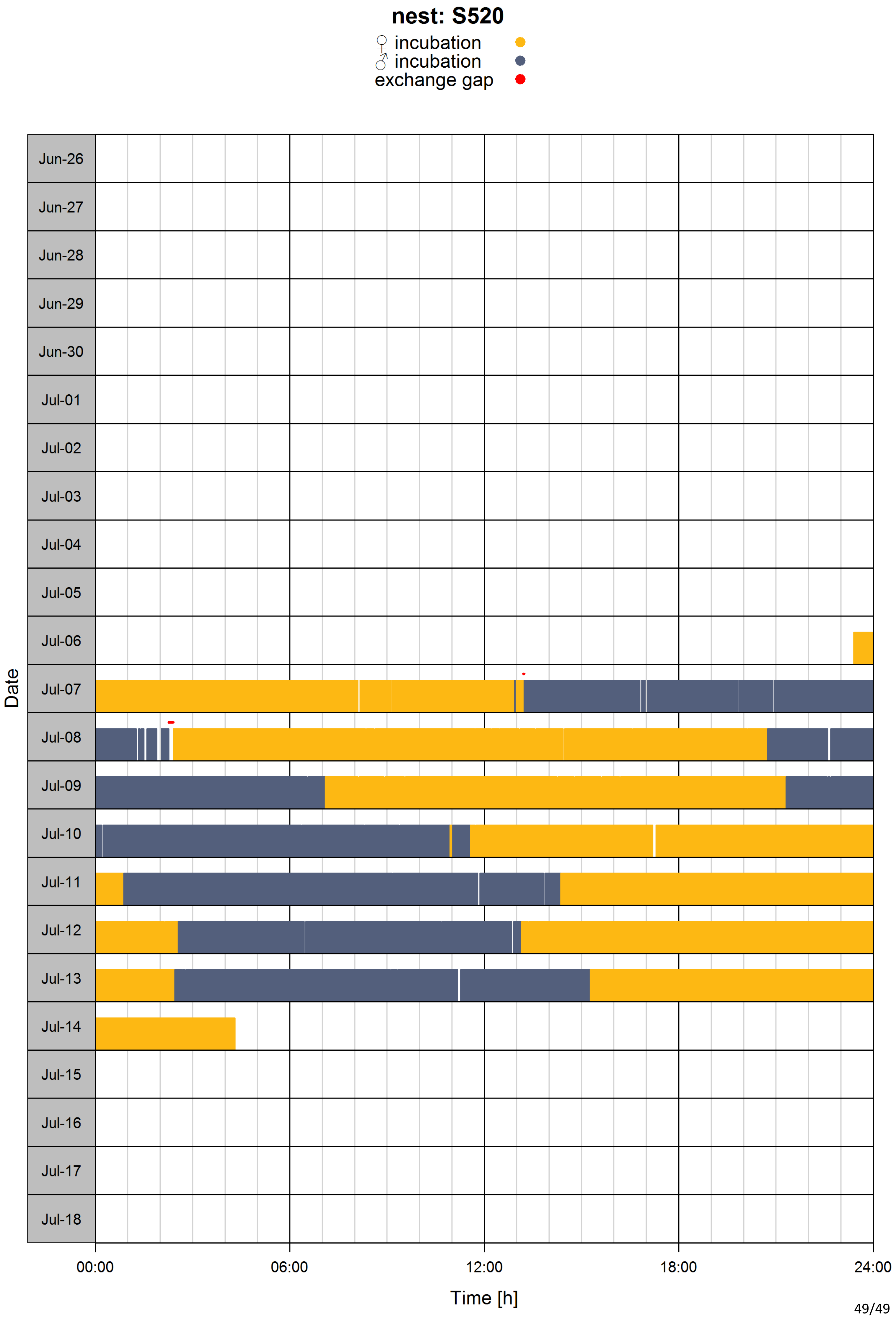
nest: S520
♀ incubation
♂ incubation
exchange gap
Date
Jun-26
Jun-27
Jun-28
Jun-29
Jun-30
Jul-01
Jul-02
Jul-03
Jul-04
Jul-05
Jul-06
Jul-07
Jul-08
Jul-09
Jul-10
Jul-11
Jul-12
Jul-13
Jul-14
Jul-15
Jul-16
Jul-17
Jul-18
00:00
06:00
12:00
18:00
24:00
Time [h]
