# Supplementary material for: Biparental incubation patterns in a high-Arctic breeding shorebird: how do pairs divide their duties?
Source: Behav Ecol. 2013 Oct 29;25(1):152–64. doi: 10.1093/beheco/art098 (PMC3860833; doi:10.1093/beheco/art098)
Supplement: Supplementary Data [file supp_art098_El.Supplement_PICTURES.pdf]

ELECTRONIC SUPPLEMENT - Pictures

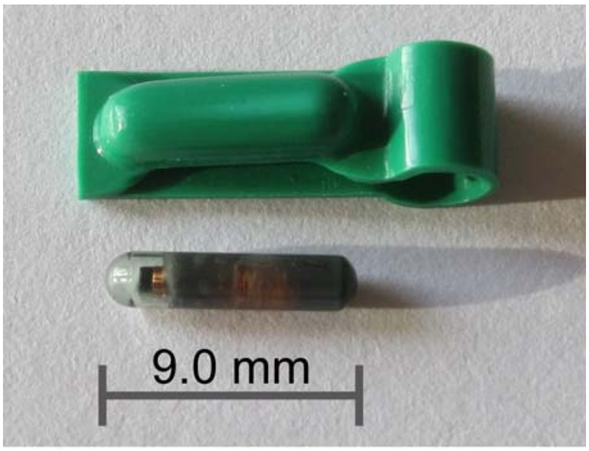

Picture S1. Glass passive-integrated RFID tag embedded in a flag. The flag was put on the tarsus and the part that sticks out was closed using an AA battery powered soldering device (Weller BP645).

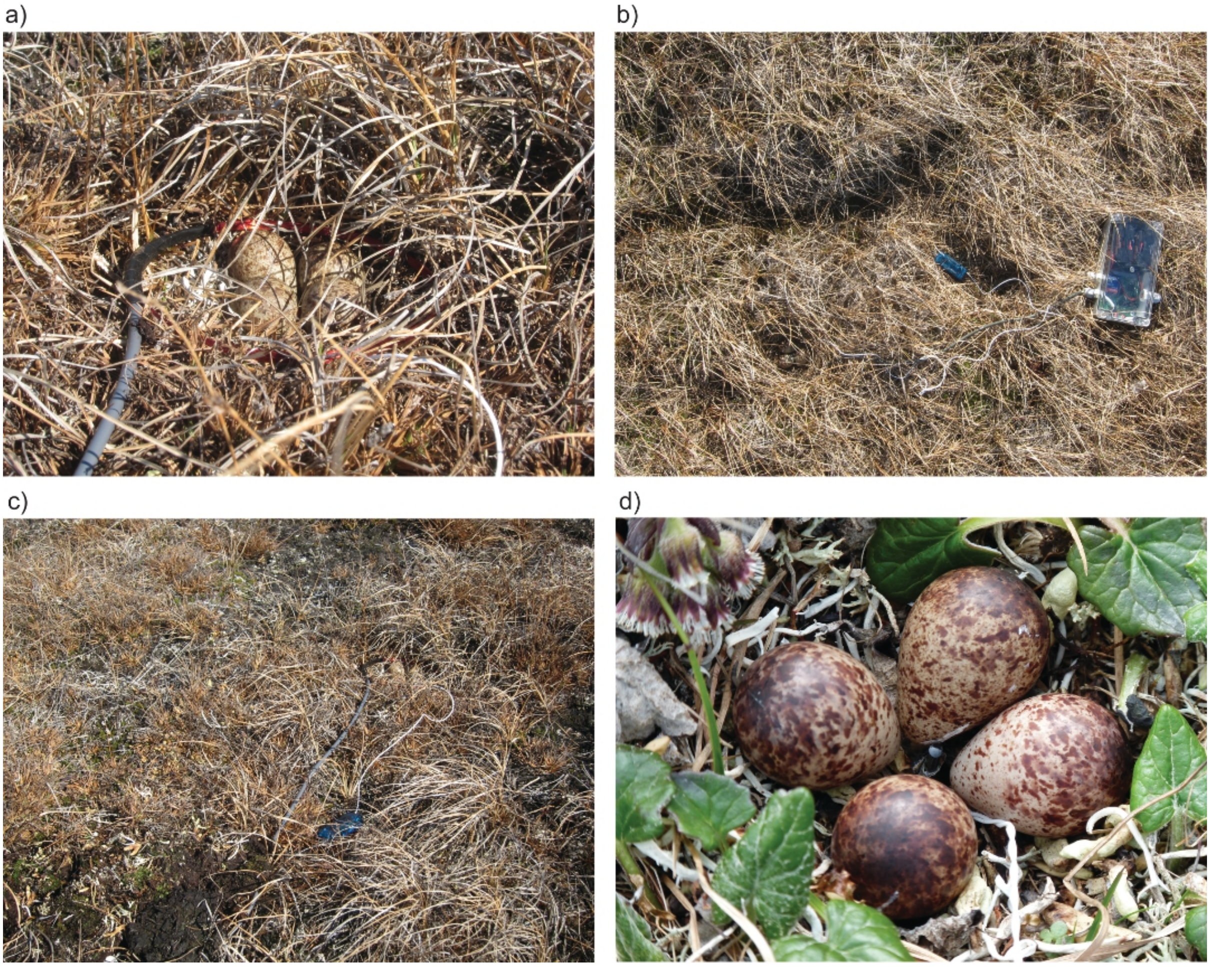

Picture S2. Incubation monitoring system. (a) The red RFID-coil detected the bird's identity. The coil was connected to the RFID reader (plastic box), placed either next to the nest in the open (b) or hidden in the vegetation (c). Data were processed by a custom board (Calima Engineering, http://www.calima.de) equipped with an EFM32-Gecko microcontroller (Energy-Micro, http://www.energymicro.com/) and stored on a 2 GB SanDisk Standard SD card. Electricity to run the system came from 6 or 12 standard AA batteries. (d) Tiny-Tag temperature probe, positioned between the eggs. This probe registered temperature in the nest (in 2 min intervals), and therefore allowed to differentiate between incubation and non-incubation. Alternatively, we used an MSR fake-egg temperature probe, connected to a data-logger (blue device in b, c). This device registered both incubation temperature and tundra temperate (every 5 s).

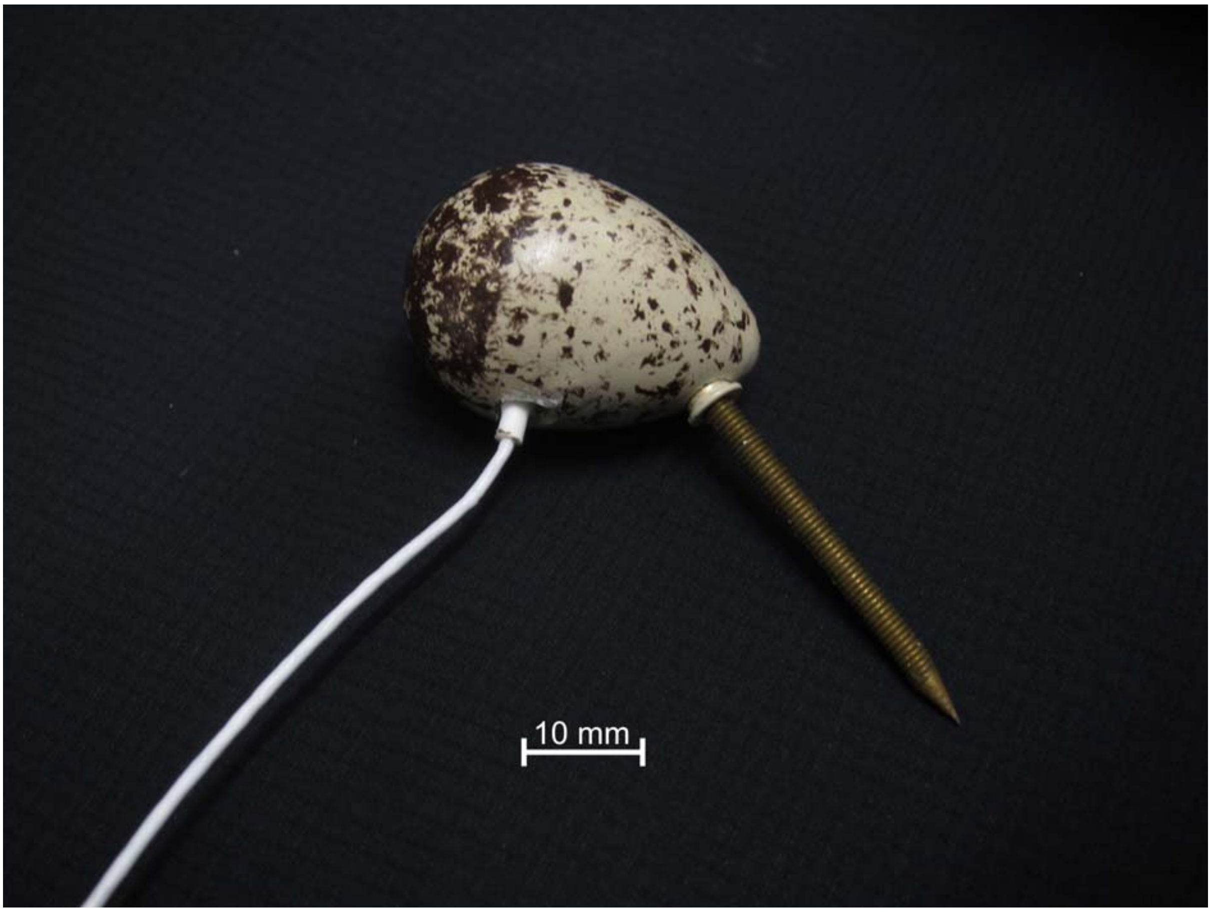

Picture S3. Fake-egg with inserted MSR temperature-probe and with attachment pin to secure it in the nest. For higher conductance the hole in the egg, which contains the probe, is filled with heat-sink grease. The probe is secured with silicon.
