# Supplementary material for: Biparental incubation patterns in a high-Arctic breeding shorebird: how do pairs divide their duties?
Source: Behav Ecol. 2013 Oct 29;25(1):152–64. doi: 10.1093/beheco/art098 (PMC3860833; doi:10.1093/beheco/art098)
Supplement: Supplementary Data [file supp_art098_El.Supplement_TABLES.pdf]

ELECTRONIC SUPPLEMENT - Tables

Table S1

Model (GLMM) estimates of median *z*-transformed incubation temperature per incubation bout in relation to sex and incubation period.

Fixed effects	*Estimate*	95% *CI*	*P*
(Intercept)	0.191	(0.103, 0.279)	< 0.0001
Day of incubation	0.008	(-0.011, 0.026)	0.70
Sex (male)°	0.039	(-0.085, 0.162)	0.86
Sex*Day of incubation	-0.001	(-0.026, 0.024)	1
Random effects	*Variance*		
Nest (intercept)	0		
z-trans (Day of incubation)	0.0004		
Residual	0.1966		

N = 307 median *z*-transformed incubation temperatures per incubation bout from 14 nests. Fixed effects, except sex, were mean-centred. Median incubation temperatures were calculated from raw incubation temperature-values *z*-transformed within each nest.

°relative to female

Table S2

Model (GLMM) estimates of incubation constancy per incubation bout (arcsine-transformed) in relation to sex and incubation period.

Fixed effects	*Estimate*	95% *CI*	*P*
(Intercept)	1.343	(1.332, 1.355)	< 0.0001
Day of incubation	0	(-0.004, 0.003)	1
Sex (male)°	-0.023	(-0.037, -0.009)	0.0002
Sex*Day of incubation	0.003	(0, 0.005)	0.098
Random effects	*Variance*		
Nest (intercept)	0		
z-trans (Day of incubation)	0.0015		
Residual	0.0058		

N = 762 incubation constancies per incubation bout from 47 nests. Day of incubation (fixed effect) was mean-centred.

°relative to female

Table S3

Model (GLMM) estimates of incubation bout length (in minutes) in relation to sex, incubation period and length of previous.

Fixed effects	*Estimate*	95% *CI*	*P*
(Intercept)	688.9	(662.9, 714.9)	<0.0001
Previous-bout length	0.4	(0.3, 0.5)	<0.0001
Day of incubation	8	(3.6, 12.5)	<0.0001
Sex (male)°	-47.5	(-70.4, -24.5)	<0.0001
Sex*Previous-bout	-0.1	(-0.3, 0)	0.20
Sex*Day of incubation	2.1	(-3.2, 7.5)	0.83
Random effects	*Variance*		
Nest (intercept)	2 142		
z-trans (Day of incubation)	140		
Residual	14 000		

N = 729 incubation bouts from 39 nests. Fixed effects, except sex, were mean-centered.

°relative to female

Table S4

Model (binomial GLMM) estimates of the probability of a detectable exchange gap in relation to sex and incubation period.

Fixed effects	*Estimate*	95% *CI*	*P*
(Intercept)	-0.357	(-1.132, 0.417)	0.65
Day of incubation	-0.167	(-0.277, -0.058)	0.0006
Sex (male)°	0.03	(-0.422, 0.481)	1
Sex*Day of incubation	-0.04	(-0.134, 0.054)	0.71
Random effects	*Variance*		
Nest (intercept)	3.21		
z-trans (Day of incubation)	0.67		

N = 762 exchange gaps from 47 nests. Day of incubation (fixed effect) was mean-centred.

°relative to female

Table S5

Model (GLMM) estimates of detectable exchange gap duration (in seconds, log-transformed) in relation to sex and incubation period.

Fixed effects	*Estimate*	95% *CI*	*P*
(Intercept)	3.953	(3.56, 4.345)	<0.0001
Day of incubation	-0.12	(-0.185, -0.055)	<0.0001
Sex (male)°	-0.11	(-0.456, 0.237)	0.87
Sex*Day of incubation	0.017	(-0.051, 0.085)	0.94
Random effects	*Variance*		
Nest (intercept)	0.46		
z-trans (Day of incubation)	0.135		
Residual	1.806		

N = 385 exchange gaps from 44 nests. Day of incubation (fixed effect) was mean-centred.

°relative to female

Table S6

Model (GLMM) estimates of incubation constancy per incubation bout (arcsine-transformed) in relation to sex and incubation period with disturbance, type of temperature probe, presence of radio-tag, length of incubation bout (h), time of a day – sin(rad), cos(rad) –, body mass and culmen length as confounding variables.

Fixed effects	*Estimate*	95% *CI*	*P*
(Intercept)	1.339	(1.325, 1.353)	<0.0001
Disturbance	-0.026	(-0.046, -0.006)	0.0029
Temperature probe type	0.006	(-0.024, 0.035)	1
Radio-tag	-0.006	(-0.033, 0.021)	1
Culmen	-0.004	(-0.015, 0.007)	0.98
Body mass	-0.001	(-0.006, 0.005)	1
Bout length	0.005	(0.001, 0.008)	0.0035
Start of incubation	-0.001	(-0.003, 0.001)	0.52
Day of incubation	-0.002	(-0.006, 0.003)	0.99
Sin (rad)	-0.011	(-0.023, 0.001)	0.12
Cos (rad)	-0.006	(-0.018, 0.006)	0.85
Sex (male)°	-0.016	(-0.033, 0.001)	0.087
Sex*Day of incubation	0.002	(-0.001, 0.006)	0.49
Random effects	*Variance*		
Nest (intercept)	0		
z-trans (Day of incubation)	0.0016		
Residual	0.0055		

N = 762 incubation constancies per incubation bout from 47 nests. Fixed effects, except sex, sin and cos, were mean-centered (culmen and body mass were centered within each sex).

°relative to female
