# Supplementary material for: Biparental incubation patterns in a high-Arctic breeding shorebird: how do pairs divide their duties?
Source: Behav Ecol. 2013 Oct 29;25(1):152–64. doi: 10.1093/beheco/art098 (PMC3860833; doi:10.1093/beheco/art098)
Supplement: Supplementary Data [file supp_art098_El.Supplement_FIGURES.pdf]

ELECTRONIC SUPPLEMENT - Figures

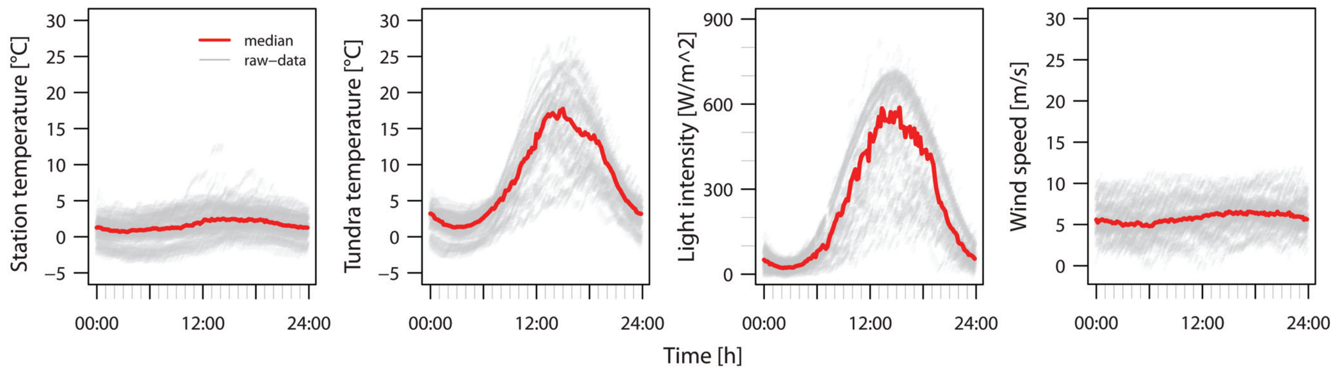

Figure S1: Daily fluctuations in (a) meteorological-station temperature (measured 2 m above the ground, NOAA Earth System Research Laboratory (http://www.esrl.noaa.gov/), (b) average tundra temperature (our measurements; details are in Methods, section *Tundra temperatures*), (c) light intensity (NOAA), and (d) wind speed (10 m above the ground; NOAA; wind > 32 m/s is hurricane) between 1 June and 16 July 2011 in Barrow, Alaska.

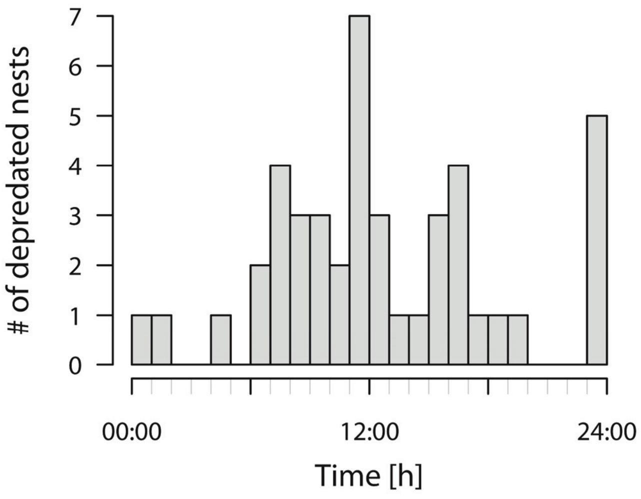

Figure S2. Daily fluctuations in predation events on semipalmated sandpiper nests. The date and time of predation corresponds to an unexpected and sudden end in incubation based on the visualised raw data from the 'incubation monitoring system'.

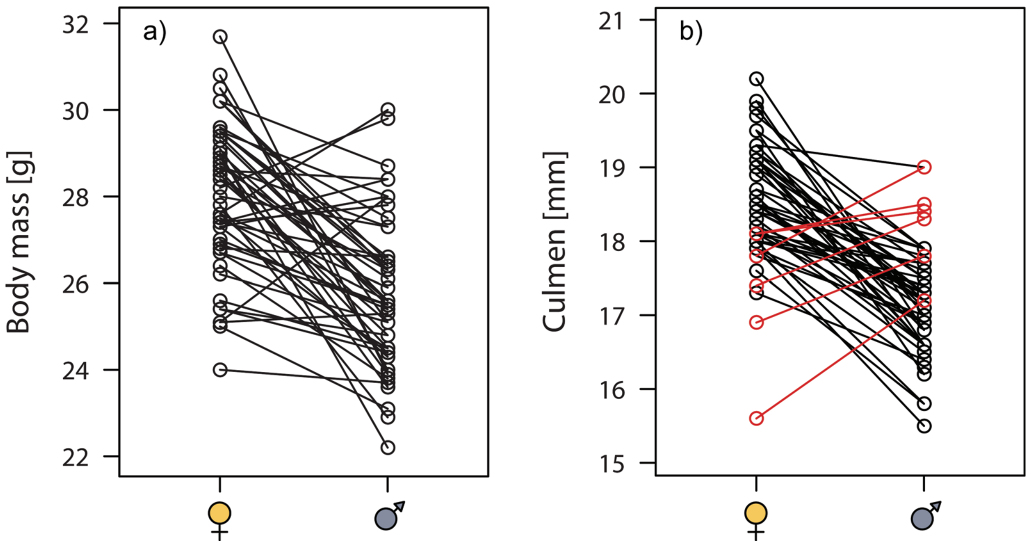

Figure S3. Variation in (a) body-mass and (b) culmen length of female and male semipalmated sandpipers. Lines connect individuals from the same nest. Red lines represent pairs with reversed dimorphism in culmen. Females were 2.2 (*95% CI*: 1.6 - 2.8) g heavier than males (Paired *t* test: t_{50} = 7, P < 0.0001), and had 1.3 (*95% CI*: 0.9 - 1.6) mm longer culmen (Paired *t* test: t_{49} = 8, P < 0.0001).

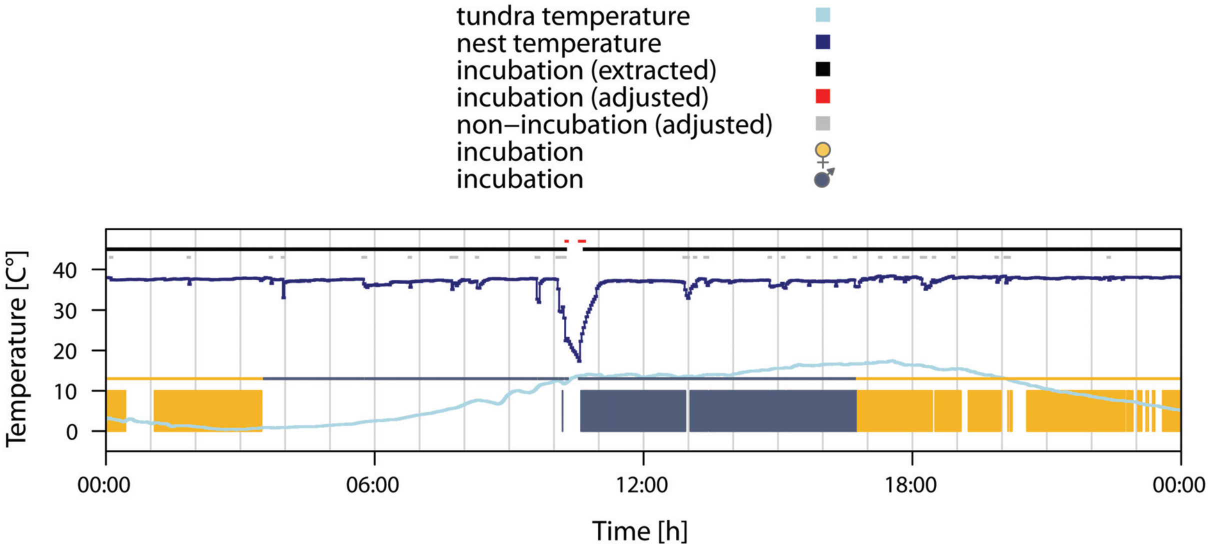

Figure S4. Example illustrating the extraction of incubation/non-incubation data.

To define periods of incubation/non-incubation from the nest temperatures, we used the following automated procedure. We assigned a time as 'incubation' (thick black line) whenever the nest temperature was above a threshold temperature, set as the running median nest temperature (with running time-window of 24h) minus 3°C (whenever the running median nest temperature was equal or above 17°C) or plus 3°C (whenever the running median nest temperature was below 17°C). Even if these conditions were not fulfilled, we assigned 'incubation' whenever the nest temperature was 12.5°C above the tundra temperature. This method missed the start of incubation after an incubation break (usually below the threshold) and the end of incubation (usually above the threshold). To correct for this, we calculated a difference in nest temperature between two consecutive readings and created a running mean of these differences with a time window of 2 minutes. If the 'running mean of the difference' was larger than a threshold (0.02°C) we considered a rise in nest temperature as incubation (red dots) and a drop in nest temperature as non-incubation (grey dots). This adjusting procedure was not applied if the nest temperature fulfilled all the following conditions: being (a) below 17°C, (b) below the running median nest temperature plus 3°C, and (c) 3°C or less above the tundra temperature. Threshold values are based on trial and error, visual assessment of the plotted data, and behaviour of birds as observed in the video recordings.

The temperature-based incubation data were overlaid with the RFID data (grey-blue and yellow vertical lines), which allowed us to assign each incubation bout to a parent. This method allowed also assignment of incubation/non-incubation to both partners even if only one bird was tagged (grey-blue line in the graph between ca. 03:30 and capture of the male at 10:30) or when the tag was not detected despite a bird's presence. The grey-blue and yellow lines represent the final assignment of incubation/non-incubation to each sex (note that some short non-incubation periods may not be visible in the graph, but are present in the data). At some nests the temperature logger malfunctioned, partially (N = 4) or completely (N = 1). If the RFID readings were continuous, incubation bouts were assigned based on the RFID data only. All automatic incubation/non-incubation assignments at all nests were checked visually and cases of obvious mis-assignment were corrected manually.

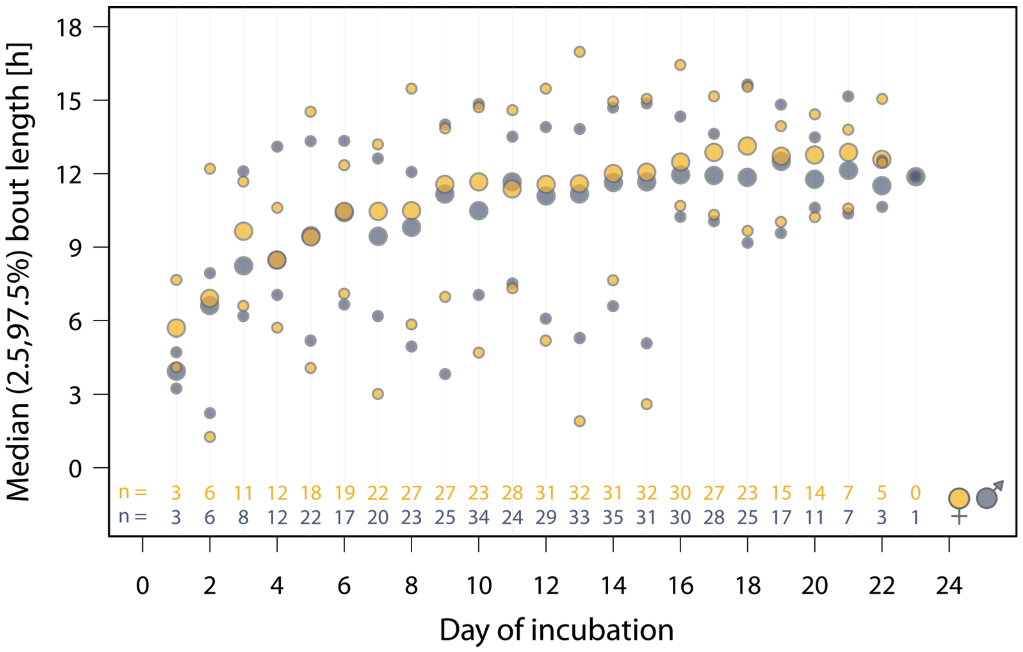

Figure S5. Increase in the length of incubation bouts across the incubation period. Large dots represent median, small dots the 2.5 and 97.5 percentile (*N* = 887 incubation bouts from 48 nests).

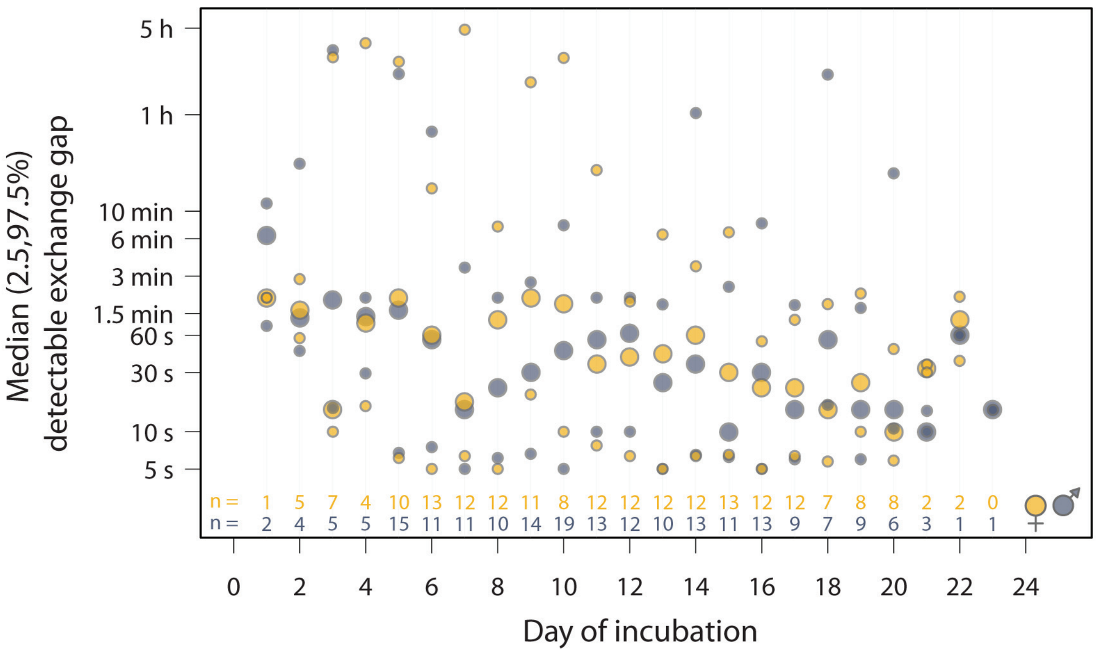

Figure S6. Decrease in the length of detectable exchange gaps across the incubation period (yellow = female is exchanging parent; grey-blue = male is exchanging parent). Large dots represent median, small dots the 2.5 and 97.5 percentile (*N* = 399 detectable exchange gaps from 44 nests). Y-axis is log-scaled.

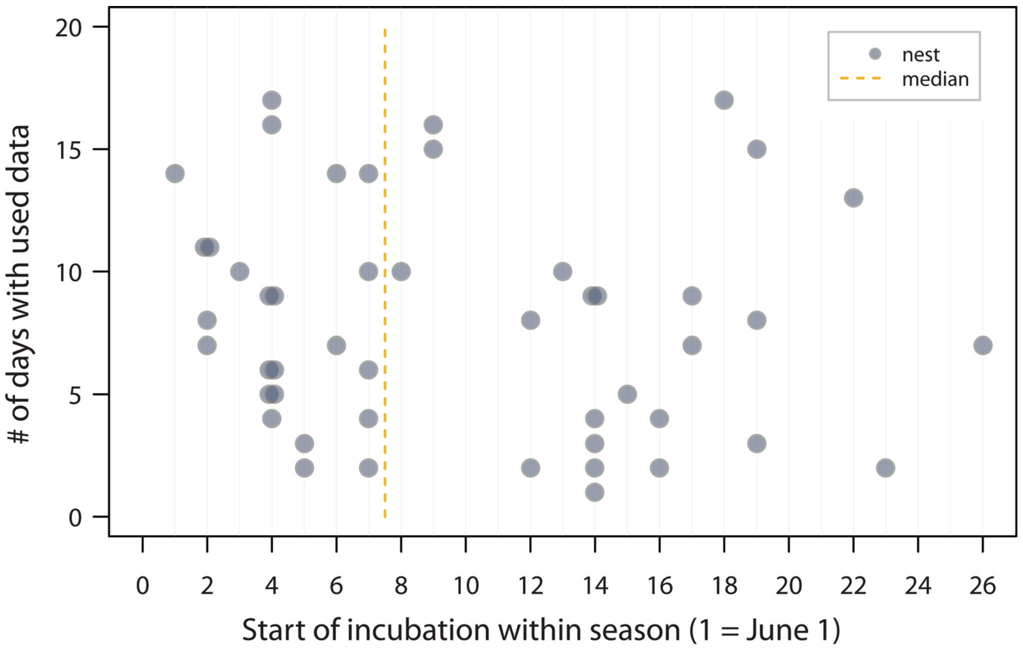

Figure S7. Distribution of the nests used in the models according to start date of incubation and the number of days for which data were included.
